# *Mycobacterium tuberculosis *complex genetic diversity: mining the fourth international spoligotyping database (SpolDB4) for classification, population genetics and epidemiology

**DOI:** 10.1186/1471-2180-6-23

**Published:** 2006-03-06

**Authors:** Karine Brudey, Jeffrey R Driscoll, Leen Rigouts, Wolfgang M Prodinger, Andrea Gori, Sahal A Al-Hajoj, Caroline Allix, Liselotte Aristimuño, Jyoti Arora, Viesturs Baumanis, Lothar Binder, Patricia Cafrune, Angel Cataldi, Soonfatt Cheong, Roland Diel, Christopher Ellermeier, Jason T Evans, Maryse Fauville-Dufaux, Séverine Ferdinand, Dario Garcia de Viedma, Carlo Garzelli, Lidia Gazzola, Harrison M Gomes, M Cristina Guttierez, Peter M Hawkey, Paul D van Helden, Gurujaj V Kadival, Barry N Kreiswirth, Kristin Kremer, Milan Kubin, Savita P Kulkarni, Benjamin Liens, Troels Lillebaek, Ho Minh Ly, Carlos Martin, Christian Martin, Igor Mokrousov, Olga Narvskaïa, Yun Fong Ngeow, Ludmilla Naumann, Stefan Niemann, Ida Parwati, Zeaur Rahim, Voahangy Rasolofo-Razanamparany, Tiana Rasolonavalona, M Lucia Rossetti, Sabine Rüsch-Gerdes, Anna Sajduda, Sofia Samper, Igor G Shemyakin, Urvashi B Singh, Akos Somoskovi, Robin A Skuce, Dick van Soolingen, Elisabeth M Streicher, Philip N Suffys, Enrico Tortoli, Tatjana Tracevska, Véronique Vincent, Tommie C Victor, Robin M Warren, Sook Fan Yap, Khadiza Zaman, Françoise Portaels, Nalin Rastogi, Christophe Sola

**Affiliations:** 1 Unité de la Tuberculose et des Mycobactéries, Institut Pasteur de Guadeloupe, Guadeloupe; 2 Wadsworth Center, New York State Dept. of Health, Albany, NY, USA; 3 Mycobacteriology Unit, Prince Leopold Institute of Tropical Medicine, Antwerp, Belgium; 4 Dept. Hygiene Microbiology and Social Medicine, Innsbruck Medical University, Innsbruck, Austria; 5 Dept of Infectious Diseases, Institut of Infectious Diseases, Milano, Italy; 6 Department of Comparative Medicine, King Faisal specialist Hospital and Research Center, Riyadh, Saudi Arabia; 7 Laboratoire de la Tuberculose, Institut Pasteur de Bruxelles, Belgique; 8 Universidad Centrooccidental Lisandro Alvarado, Barquisimeto, Venezuela and Universidad de Zaragoza, Spain; 9 All India Institute of Medical Sciences, New Delhi, India; 10 Biomedical Research and Study Center, Riga, Latvia; 11 Institut for Hygiene, Microbiologie and Tropical Medicine, Austria; 12 Universidade Federal do Rio Grande de Soul, Brazil; 13 Instituto de Biotecnologia INTA, Castelar, Argentina; 14 Dept of Medical Microbiology and Pathology, faculty of Medicine, University of Malaya, Kuala Lumpur, Malaysia, School of Public Health; 15 University of Düsseldorf, Heinrich-Heine-University, Düsseldorf; 16 Dept of Internal Medicine II, University of Regensbourg, Germany; 17 Public Health Laboratory, Hearltlands Hospital, Birmingham, UK; 18 Dept of Clinical Microbiology and Infectious Diseases, Hospital Gregorio Marañon, Madrid, Spain; 19 Dept. of Experimental Pathology, Medical Biotechnology, Infection and Epidemiology, Pisa University, Pisa, Italy; 20 Laboratory of Molecular Biology applied to Mycobacteria, Dept. Mycobacteriosis, Oswaldo Cruz Institute, Rio de Janeiro, Brazil; 21 Centre National de Référence des Mycobactéries, Institut Pasteur, Paris, France; 22 MRC Centre for Molecular and Cellular Biology, Dept of medical Biochemistry, University of Stellenbosch, Tygerberg, South Africa; 23 Laboratory Nuclear Medicine Section, Isotope group, Bhabha Atomic Research Centre c/T.M.H. Annexe, Parel, Mumbai-400012, India; 24 Public Health Research Institute, Newark, NJ, USA; 25 Mycobacteria reference unit, Diagnostic Laboratory for Infectious Diseases and Perinatal Screening, National Institute of Public Health and the Environment, Bilthoven, The Netherlands; 26 Municipal Institute of Hygiene, Prague, Czech Republic; 27 Statens Serum Institute, Int. Ref. lab. for Mycobacteriology, Copenhagen Denmark; 28 Institute of Hygiene and Epidemiology, Hanoi, Vietnam; 29 Universidad de Zaragoza, Zaragoza, Spain; 30 Laboratoire de Bactério-virologie-hygiène, CHU Dupuytren, Limoges, France; 31 Institut Pasteur de Saint-Petersbourg, Saint Petersbourg, Russia; 32 Bavarian Health and Food Safety Authority, Oberschleissheim, Germany; 33 Forschungszentrum, National Reference Center for Mycobacteria, Borstel, Germany; 34 Dept of Clinical Pathology, Padjadjaran University, Dr. Hasan Sadikin Hospital, Bandung, Indonesia; 35 Tuberculosis Laboratory, International Centre for Diarrhoeal Research, Dhaka, Bangladesh; 36 Institut Pasteur de Madagascar, Tananarive, Madagascar; 37 Dept of Genetics of Microorganisms, University of Lódz, Lodz, Poland; 38 Servicio Microbiología, Hospital Universitario Miguel Servet, Zaragoza, Spain; 39 State Research Center for Applied Microbiology, Obolensk, Russian Federation; 40 Dept. of Respiratory Medicine School of Medicine Semmelweis University, Budapest, Hungary; 41 Veterinary Sciences Division, Department of agriculture for Northern Ireland, Belfast, UK; 42 Centro regionale di Riferimento per i Micobatteri, Laboratorio de Microbiologia e Virologia, Ospedale Careggi, Firenze, Italy

## Abstract

**Background:**

The Direct Repeat locus of the *Mycobacterium tuberculosis *complex (MTC) is a member of the CRISPR (Clustered regularly interspaced short palindromic repeats) sequences family. Spoligotyping is the widely used PCR-based reverse-hybridization blotting technique that assays the genetic diversity of this locus and is useful both for clinical laboratory, molecular epidemiology, evolutionary and population genetics. It is easy, robust, cheap, and produces highly diverse portable numerical results, as the result of the combination of (1) Unique Events Polymorphism (UEP) (2) Insertion-Sequence-mediated genetic recombination. Genetic convergence, although rare, was also previously demonstrated. Three previous international spoligotype databases had partly revealed the global and local geographical structures of MTC bacilli populations, however, there was a need for the release of a new, more representative and extended, international spoligotyping database.

**Results:**

The fourth international spoligotyping database, SpolDB4, describes 1939 shared-types (STs) representative of a total of 39,295 strains from 122 countries, which are tentatively classified into 62 clades/lineages using a mixed expert-based and bioinformatical approach. The SpolDB4 update adds 26 new potentially phylogeographically-specific MTC genotype families. It provides a clearer picture of the current MTC genomes diversity as well as on the relationships between the genetic attributes investigated (spoligotypes) and the infra-species classification and evolutionary history of the species. Indeed, an independent Naïve-Bayes mixture-model analysis has validated main of the previous supervised SpolDB3 classification results, confirming the usefulness of both supervised and unsupervised models as an approach to understand MTC population structure. Updated results on the epidemiological status of spoligotypes, as well as genetic prevalence maps on six main lineages are also shown. Our results suggests the existence of fine geographical genetic clines within MTC populations, that could mirror the passed and present *Homo sapiens sapiens *demographical and mycobacterial co-evolutionary history whose structure could be further reconstructed and modelled, thereby providing a large-scale conceptual framework of the global TB Epidemiologic Network.

**Conclusion:**

Our results broaden the knowledge of the global phylogeography of the MTC complex. SpolDB4 should be a very useful tool to better define the identity of a given MTC clinical isolate, and to better analyze the links between its current spreading and previous evolutionary history. The building and mining of extended MTC polymorphic genetic databases is in progress.

## Background

Each year, 9 million new cases of tuberculosis (TB) are recorded, of which 2 million result in fatality. Diagnostics, chemotherapy and vaccination are available, however, the disease is far from being eradicated [1]. Many genetic loci within the *Mycobacterium tuberculosis *complex (MTC) genomes are polymorphic and may be used for molecular evolutionary studies [2]. Among these, the Direct Repeat locus (DR), which consists of alternating identical DRs and variable spacers can be assessed using the "Spoligotyping" fingerprinting method thousands of different patterns [3]. DR loci are members of a universal family of sequences, designated as CRISPR [4], whose physiological role is poorly known [5,6]. Spoligotyping was previously shown to be useful for both clinical management and molecular epidemiology of MTC [7]. When used in association with variable-number of DNA tandem-repeat (VNTR) [8] or Mycobacterial-interspersed-repetitive-units (MIRU) [9], spoligotyping is a fast, robust, and cost effective genotyping technique, alternative to traditional IS*6110*-RFLP fingerprinting. These methods are also designated as MLVA (Multiple-locus variable number tandem repeats analysis) [10]. Since 1999, we have built and released genetic diversity databases of the MTC DR locus as an attempt to analyze MTC population structure, and to assess the complexity of global TB transmission and of the underlying spatial and temporal evolution of the TB genetic landscape. Indeed, previous studies have shown that the host's geographical origin is predictive of the clinical isolate of tuberculosis being carried, since there is an apparent stable association of TB bacilli populations with their human hosts in various environments [11], hence a strong phylogeographical clustering of TB bacilli population. We hypothesize that co-evolution between human beings and bacilli, and vertical transmission (in the household), must have been the main mode of tuberculosis transmission throughout centuries and even millenniums [12].

MTC organisms were also shown to evolve clonally [13]. Hence, the reconstruction of the population structure of this species may be an indirect way of assessing its main host's (*Homo sapiens sapiens*) migratory and demographic history [14]. Indeed, tuberculosis may have affected early hominids and it is tempting to speculate that the MTC originated in East-Africa [15]. Its expansion to the rest of the world may have coincided with the waves of human migration out of Africa, with potential back migration from Asia to Sub-Saharan Africa [16]. If the past phylogeny of MTC is likely to have involved horizontal gene transfer events, however, these events are no longer observed [17]. All these clues suggests the pioneer roles of geography, demography and human migration history in shaping today's MTC population structure [11]. Consequently, the current concepts of "natural evolving communities" or "clusters of bacilli", are important: (1) for TB epidemiology – as the global pandemia should be considered as a network of outbreaks of more or less circumscribed clones [18,19] – (2) for molecular ecology, evolutionary and population genetics and phylogeography, – as today's MTC genomes are likely to include cryptic information on their passed and present history in their changing environments [20,21] – and last but not least (3) for systematics and infra-species taxonomy [22]. The two first databases were poorly representative of the worldwide MTC diversity [23,24], whereas the third update was already more representative [25,26]. Although these studies did not allow for a definitive rebuilding of the MTC limb and twig history of the TB tree, their use combined to bioinformatical data-mining methods, allowed to previously classify MTC in eight to ten main genetic lineages [27], a classification that has now been validated using SNPs [28,29].

In this new study, we data-mined an updated SpolDB4 version, which contains 1939 STs representing a total of 39,295 clinical isolates originating from 122 countries. Considering the known diversity of the origin of patients, which was documented in some cases, SpolDB4 is representative of a total of 141 countries. The SpolDB4 update adds 1126 newly defined STs and provides a higher resolution picture of the worldwide MTC genome diversity assessed by spoligotyping. However, the new challenge is to link the genetic variability of MTC with the clinical variability of TB, whatever the setting, whether in high burden countries or in densely populated areas such as India or China.

## Results

### Classification of spoligotypes

#### Classification of SpolDB4 spoligotype patterns into meaningful lineages

**Figure 1:**
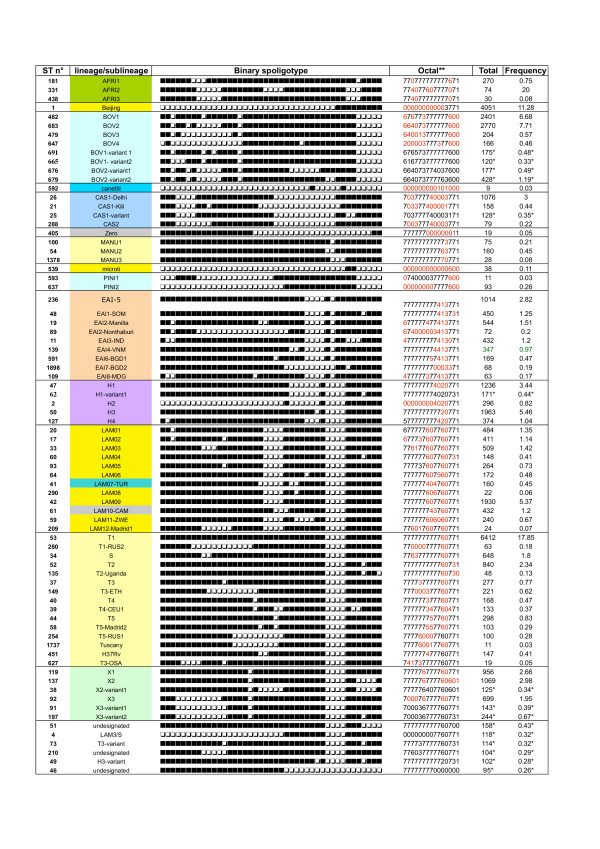
Bioinformatical (62 lineages/sub lineages prototype patterns) and statistical (50 most frequent) classification analysis of SpolDB4. First column ST n°: Shared-type (ST) number of prototype pattern for the linage/sub lineage. Second column: lineage/sub lineage name. Third column: Binary spoligo display with black-white squares for respectively hybridizing-non-hybridizing spacers. Fourth column: Octal code (in red: defining octal rule). Fifth column: total absolute number of isolates of the subclass when variant ST spoligos are included (using SpolNet). Sixth column: same but expressed as percentage of total clustered isolates. * Total number and Frequency for these types are already included in their mother clade if known. Undesignated types are counted within the T1-ill-defined lineage. ** in red: octal rule defining the genotype.

The listing of ST alleles with their distribution by country of location and a presumed sub lineage/lineage label is provided in additional file 1. 51 new countries are represented in SpolDB4. Out of 39,295 spoligotypes patterns, n = 35,925 are found in 1939 STs (91.4% of the isolates,) and 3370 (8.6%) are orphan patterns, totalling 5,309 individual alleles. Two approaches, a statistical, and a mixed expert-based/bioinformatical one were used to data-mine SpolDB4 to classify spoligotypes. Results are summarized in Figure 1.

#### Results obtained by the statistical approach

The 20 most frequent STs totalled 17,701 isolates (49.3% of the clustered isolates). The 50 most frequent STs increased clustering to 61.8% (n = 22,219). These 50 most frequent types are shown in figure 1. Three types did not receive a lineage label, ST46, ST51 and ST210. ST46 and ST51 are patterns prone to genetic convergence, similarly as reported for ST4, whose ancestors can either be ST33 or ST34 [30]. ST210 (also designated as HN24, a Principal Genetic Group (PGG) 3 strain) was first described in a study done in Texas and is almost restricted to the USA [31]. The other 47 most prevalent spoligotypes belong to known genetic lineages or are defined variants.

#### Results obtained by the mixed expert-based/bioinformatical approach

Figure 1 also describes a total of 62 remarkable lineages/sub lineages. This classification was obtained with the use of a dedicated software that search for similarities between patterns (SpolNet, P. Abdoul *et al*. unpublished, see material and methods section). Since SpolDB4 is a mixed *M. tuberculosis *(human) and *M. bovis *(human or bovine) isolates database, 237 STs were found to belong to the *M. bovis *subspecies (n = 5710), whereas 1702 STs (n = 33,585) were not *M. bovis*. The calculation of the genetic diversity index "H" -defined as H = 2n (1 - Σx_i_^2^)/2n-1 where n = number of individuals and x_i _is the frequency of the i^th ^allele-gives a value of 0,98. H only slightly improved (+0.6%) compared to SpolDB3. This shows that the exponential increase of data was not reflected by the increase in bacterial diversity description, as most data were already known (over fitting phenomenon, a limitation of this study). Indeed, a quantitatively updated SpolDB3 (on 817 STs) would cluster 31,292 isolates, whereas the new 1122 STs aggregate only 4633 additional clustered isolates. Further improvement of H would require new data entries, possibly from yet unrepresented settings and/or high TB-burden countries such as India and China.

#### New genetic lineages within *M. tuberculosis *complex

SpolDB4 defines 62 genetic lineages/sub lineages (figure 1). *M. bovis *strains were divided in 3 sub lineages corresponding to ST prototypes 482, 683 and 479. A new signature for *M. microti *is suggested (presence of spacers sp37-38, ST539) [32], instead of ST641 in SpolDB3. *M. caprae *and 2 sub lineages of *M. pinipedii*, a new member of MTC [33], were added. Within the *M. africanum *subspecies, more is known today on its taxonomical status, thanks to improvement of spoligotyping through the 68 spacers format and thanks to the discovery of other lineage-specific genetic markers [34,35]. Using a dedicated software (*structure *version 2, [36,37]) to infer the population structure of the *M. africanum *spoligotyping dataset of SpolDB4, one would suggest the existence of at least 4 populations in SpolDB4 (results not shown), however more data on the genetic diversity of *M. africanum *will be required to be able to get a clearer picture of the global population structure of this pathogen.

#### New genetic lineages within *M. tuberculosis *stricto sensu

Among *M. tuberculosis *stricto sensu, new visual rules defining 22 lineages/sub lineages are described. The previously defined Central-Asian (CAS) lineage was split into CAS1-Delhi type (ST26) found mainly in India and in the Indian subcontinent [38,39], and CAS1-Kilimanjaro (ST21) found in Tanzania [40]. Within the East-African-Indian (EAI) lineage, new prototypic spoligotyping-signatures for 4 sub lineages are presented (EAI2-Nonthaburi, EAI6-Bangladesh/1, EAI7-Bangladesh/2 and EAI8-Madagascar). Douglas *et al. *designated the EAI2 clade as the "Manila family" [41]. We further linked the Nonthaburi group of strains from Thailand [42] to this lineage (results not shown). EAI3 and EAI4, are now being shown as phylogeographically specific from India and Vietnam respectively, with suggested designations of EAI3-IND and EAI4-VNM. Two new lineages from Bangladesh are found, designated as EAI6-Bangladesh/1 (58.1% of isolates from Bangladesh) and EAI7-Bangladesh/2 (91.2% of isolates from Bangladesh). EAI6-BGD1 harbours specificity for the eastern part of the South Asian region since it is also found in neighbouring Myanmar (results not shown).

Within the Haarlem (H) lineage, a 4th sub lineage (H4) is tentatively added. It is characterized by the absence of spacers 29–31 and 33–36 (prototypes ST127 and/or ST777). More than 60% of ST127 isolates are localized in Armenia, Austria, Finland, Georgia, Iran, and Russia. A likely related pattern (ST777) is found in Saudi Arabia. An hypothesis is that these strains could represent an intermediate genetic link between the previously defined Haarlem-1 (H1, ST 47) and Haarlem-3 (H3, ST50) genotypes. Type ST777, which shows a single spacer difference from ST 127, is also found in Kazakhstan, Russia, and Georgia (n = 26). These isolates are likely to be identical to the recently described "Ural" family of strains [43]. They suggest the prominent role of Central Asia as a hub of migratory routes of *Homo sapiens sapiens *and its role in the history of infectious diseases.

Within the Latin-American-Mediterranean (LAM) lineage, we rebaptised the LAM7 sub lineage as LAM7-Turkey since recent results suggested that ST41 is predominant in Asia Minor [44]. Similarly, the LAM10 sub lineage was renamed as LAM10-Cameroun [45,46]. Two sub lineages are new, LAM11-ZWE (ST 59) with 57.8% of isolates originating from Zimbabwe [47], and LAM12-Madrid1 (ST209) [48]. The LAM11-ZWE is likely to be identical to the recently described Meru family found in Tanzania [40]. The "Manu" family, a new family from India, which could be an ancestral clone of principal genetic group 1 strains [39], is tentatively sub-divided into Manu1 (deletion of spacer 34), Manu2 (deletion of spacers 33–34), and Manu3 (deletion of spacers 34–36). The central role of India, and more generally of Asia in tuberculosis evolutionary history is more and more evident. The S lineage which is highly prevalent in Sicily and Sardinia, could be identical to the F28 clade in South Africa [30,49]; the existence of this genotype family was confirmed in SpolDB4, however its origin remains unknown. The "T" families (modern TB strains) stayed ill-defined with more than 600 unclassified STs. They were stratified into 5 subclades (T1-T5) based on single-spacer differences. 8 nested clades, with robust spoligotyping-signatures were extracted; with the exception of "Tuscany", their names were built using their proximate upper-clade designation (T1 to T5), followed by their presumed geographical specificity: T3-Ethiopia (ST149); T5-Russia/1 (ST254), T1-Russia/2 (ST280), T3-Osaka (ST627), T5-Madrid/2 (ST58), T4-Central Europe/1 (ST39), T2-Uganda (ST135), and "Tuscany" (ST1737). ST149 was previously shown to be frequent in Ethiopia and in Denmark among Ethiopian immigrants. This low-banding IS*6110 *clone had been identified based on IS*6110*-RFLP as early as 1995 and represented 36.2% of isolates from this country [50]. ST254 and ST280 were repeatedly isolated from clinical isolates in Russia, in former Russian soviet republics and in Northern and Eastern European countries (Estonia, Finland, Georgia, Latvia, Poland, Russia). ST1737, with a single spacer difference from ST254, was recently found in Italy and designated as "Tuscany" [51]. ST627 was identified for the first time in Finland and repeatedly found in the Okayama district and elsewhere in Japan [52]. T5-Madrid/2 isolates were previously found to be characteristic of Spanish-related settings [48]. T2-Uganda, which was first described by Niemann *et al*., was repeatedly found in East Africa, and at least 7 STs with a prototypic signature are linked to East-Africa. Last but not least, the T4-CE1 (for Central-Europe/1) was tentatively identified based on similarity between ST39 found in Europe and South Africa, and some likely derived genotypes found both in South and North America (ST94, ST430, ST1258). Among those, ST1258 represents the most prevalent spoligotype detected by the Inuit's community in Nunavik, Canada. Whether this type has been introduced by a casual European contact into this community, or has been endemic among the Inuits is currently under investigation [53].

The X genotype family (X1-X3 sub lineages) was initially described thanks to data-mining [27]. This family is today a well-characterized IS*6110 *low-banding family, duly characterized by IS*6110*-RFLP as well as by high-throughput genetic methods [54-57]; it is prevalent in UK, in USA and in former British colonies. Lastly, a Vietnamese genotype family characterized by the absence of IS*6110 *insertion elements, was shown to bear a specific spoligotyping signature which is characterized by the deletion of spacers 19–41 (ST 405), and was designated as the "Zero copy" clade [58].

#### Comparison between the supervised and an unsupervised Naïve Bayes statistical approach of classification of spoligotypes

Data-mining and clustering techniques are the focus of intense research in Information and Bio Sciences [59]. Classification of spoligotypes, given the almost infinite dimension of theoretical allelic number -n = 2^43 ^in the current format that does not detect the complete set of known spacers-, is not a trivial task. A recent attempt to use a statistical approach of classification of spoligotypes through the utilization of a Naïve Bayes algorithm and a mixture model was suggested [60]. A good correlation was found between the two techniques for a large number of genotype families defined, with strong support of the stability value. However, the mixture model also suggested the existence of new spoligotype signatures and a total of 48 families (N1 to N48) [60]. Some of these signatures are confirmed by this study, others are not. A similar approach using a Markov model is currently under development in our laboratory.

#### Unclassified spoligotypes

454 STs (23.4%) did not correspond to any pattern recognition rule when data were mined automatically using SpolNet. Nonetheless, we attributed a family label to these spoligotypes too by extending the rules defined initially, and subsequently by a visual assessment on each unclassified ST. Thus, 314 more STs were tentatively classified with a family label, and only 131 STs (7% of the total) remained unclassified. These STs harboured patterns with either important blocks of deletion, disseminated deletions, mixed signatures, or patterns, which at this time did not correspond to any known spoligotype-signatures.

## Results

### Population genetics

The worldwide distribution of data points was assessed within eight regions. Isolates from Europe and North America represented 65.5% of all entries. Africa, Far-East Asia, Middle East and Central Asia, and South America were equally represented (6.5% to 8.3% of entries) whereas Central America and Oceania were underrepresented (3.6% and 1.1% of entries). The overrepresentation of *M. bovis *from Europe and South America (about 30 and 25% respectively) limits the interpretation on global *M. bovis *genetic diversity, but also reflects the reality of the importance of beef cattle economy in South America (Brazil and Argentina) and Europe. Orphan spoligotypes, represented 35.7% and 16.9% of the isolates in Europe and North America respectively, and ranged from 1.2% to 11.5% in the other regions.

**Figure 2:**
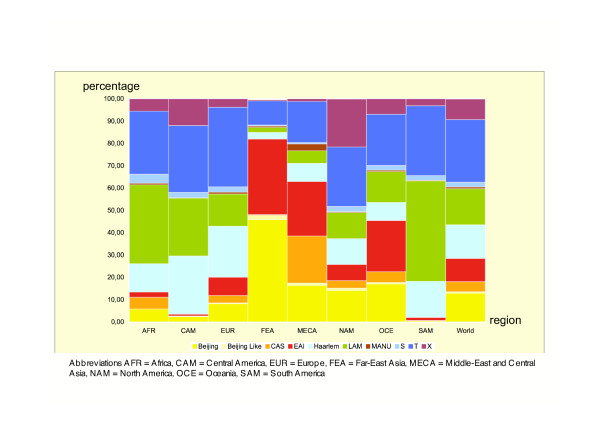
Percentage of main spoligotyping-defined MTC genotype families within SpolDB4 (Beijing, Beijing-like, CAS, EAI, Haarlem, LAM, Manu, X, T), by studied continents and worldwide. Abbreviations : AFR = Africa, CAM = Central America, EUR = Europe, FEA = Far-East Asia, MECA = Middle-East and Central Asia, NAM = North America, OCE = Oceania, SAM = South America.

**Figure 3:**
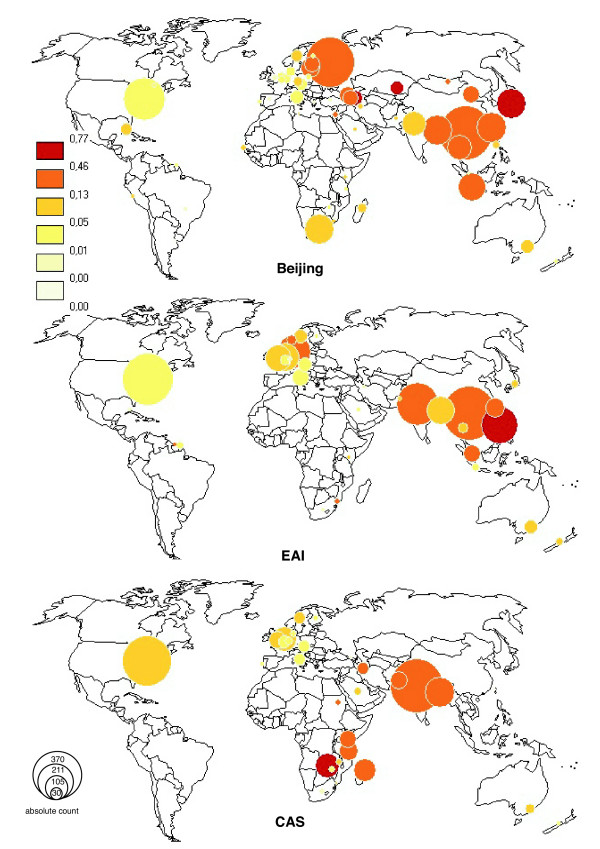
Synthesizing World Maps showing absolute (diameter) and percentage (colour) numbers of 3 genotype families within each country: Beijing; EAI (East-African Indian) CAS (Central Asia). These maps were built on an updated SpolDB4 on 2005 September 14^th^, on clusters of the 50 most frequent shared types as shown in Table 1, for a total of n = 17212 isolates (Beijing n = 4042, EAI n = 1684, CAS n = 1022).

Figure 2 is a synthetic histogram of the distribution of 10 main lineages in the studied continents. In brief, Beijing and Beijing-like strains represent about 50% of the strains in Far East-Asia and 13% of isolates globally. In Europe, the Haarlem lineage represents about 25% of the isolates. In South America, about 50% of the strains belong to the LAM family. Three major genotypic families (Haarlem, LAM, and T) are the most frequent in Africa, Central America, Europe and South America. Outside Europe, The Haarlem strains were mainly found in Central America and Caribbean (about 25%), suggesting a link of Haarlem to the post-Columbus European colonization [61] (Figure 3). The presence of the LAM family is highest in Venezuela (65%) [62], in the Mediterranean basin (e.g. 34% in Algeria, 55% in Morocco, 30% in Spain), and in the Caribbean region (30% in Cuba and Haiti, 17.4% in French Guiana, 15% in French Caribbean islands) (Figure 3A). The "ill-defined" T genetic family, was found in all continents, and corresponded to about 30% of all entries in the database. Undoubtedly, MLVA and/or SNPs data will improve the knowledge of the identity of these isolates designated as "T" by default [26].

The Beijing family of strains is prevalent in Far-East-Asia, but also in Middle-East-Central Asia and Oceania (45.9%, 16.5% and 17.2% respectively) (Figure 3). The Beijing genotype which may have been endemic in China for a long time [63] is emerging in some parts of the world, especially in countries of the former Soviet Union, and to a lesser extent in the Western world [64]. The East-African-Indian (EAI) family is also highly prevalent in these areas (33.8% in Far-East-Asia, 24.3% in the Middle East and Central Asia, and 22.9% in Oceania). The EAI lineage is more prevalent in South-East Asia, particularly in the Philippines (73%; [41]), in Myanmar and Malaysia (53% ; [65]), in Vietnam and Thailand (32% ; [62]) (Figure 3).

The CAS1-Delhi family is essentially localized in the Middle-East and Central Asia, more specifically in South-Asia, (21.2%), and preferentially in India (75%; [38,66]). It is also found in other countries of this region such as Iran, and Pakistan [67,68]. It has also been found in several others regions (Africa, 5.3%; Central America, 0.1%; Europe, 3.3%; Far-East-Asia, 0.4%; North-America, 3.3%; Oceania, 4.8%). In Europe and Australia, these strains were frequently found to be linked with immigrants from South Asia [24].

Lastly, the X family is highly prevalent in North America (21.5%) and Central American (11.9%) regions. It could be linked to an Anglo-Saxon ancestry, as it has been encountered in English-colonized areas such as in the United Kingdom, United States, Australia, South Africa and in the Caribbean [27]. However, according to other investigators, this group of strains is currently correlated with African-Americans, a fact however that may not represent the ancestry of this genotype [69]. More studies should be done to clarify this issue.

#### Analysis of the spreading and epidemiological status of MTC clones

We further analyzed the epidemic status of each spoligotyping-defined clone as shown by the index C1 and C2 [26] as well as the inter-continental match between STs. Results are shown in Table 1 and Table 2. Briefly, the Spreading Index (SI) represents a mean number of occurrences of a clone, independent of the setting. Fourteen types were defined as "epidemic" (SI>25), 65 as "common"(10<SI<25), 669 as "recurrent" (SI<10) and 1090 as "rare" (SI ≤ 2). The Table 1 shows the results based on combination of C1 and C2 which provides 12 classes ranging from the highly localized (endemic) and numerous (epidemic), to the highly spread (ubiquitous) and infrequent (rare) genotypes. When analyzed geographically using the Matching Code (MC), a total of 824 types are found within a single macro region ("Endemic", 42,5%), and 564 types are present in exactly two settings ("localized", 29%). Types present in three macro regions but found in five or less areas are also defined as localized and totalled 246 types. The Intercontinental match of these types was not analyzed further. All other types, being present in at least three continents and in at least six areas, or present in four or more continents, (n = 551), were declared as "ubiquitous". The Table 2 shows the number of endemic types per continent and presents the matching results between "localized" types. Endemic types are likely to represent local clones, current end points of evolution, either because of extinction (for ancient clones) or because epidemiological transmission was not yet followed by mutation for emerging ones. Independently of recruitment, the number of endemic types is minimal in Oceania and Central America (0.005 endemic type/occurrence), two regions that have experienced a negative migratory balance for centuries. In all other continents, with a 0.015 to 0.03 endemic type/occurrence, and slightly more in Middle-East and Central Asia (0.037), rates of endemism appear to be similar, a feature resulting from a combination of the intrinsic molecular clock of the DR locus, the age of the tubercle bacilli, and the historical tuberculosis transmission waves.

#### Toward an interactive system of geographic atlas of genotype frequency data of *Mycobacterium tuberculosis*

**Figure 4:**
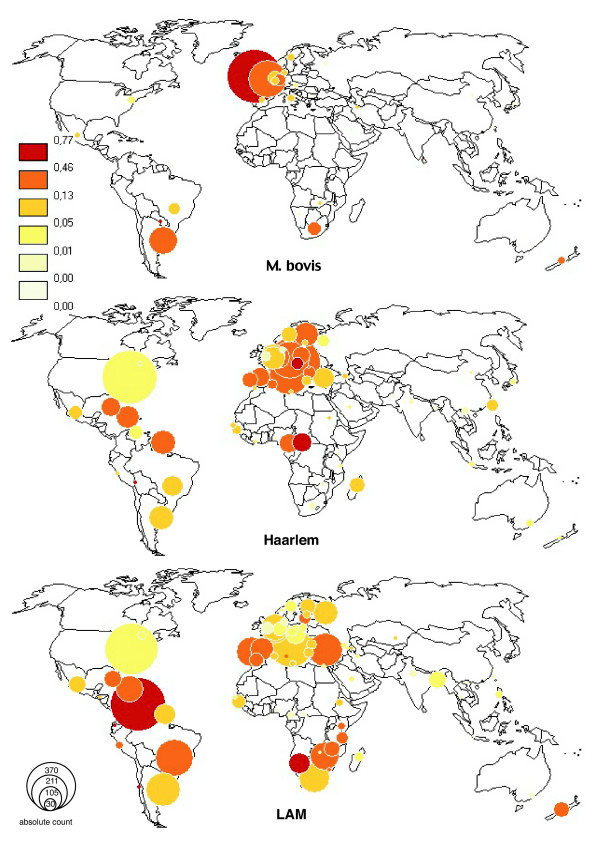
Synthesizing World Maps showing absolute (diameter) and percentage (colour) numbers of 3 genotype families within each country: *M. bovis*; Haarlem; Latin-American and Mediterranean (LAM). These maps were built on an updated SpolDB4 on 2005 September 14^th^, on clusters of the 50 most frequent shared types as shown in figure 1, for a total of n = 17212 isolates (*M. bovis *n = 3888, LAM n = 3400, Haarlem n = 3176). Maps were built using Philcarto (P. Waniez, version 4.38).

Figure 3 and 4 illustrate a mixed representation (by absolute case number and by frequency) of the distribution of the most frequent STs shown in Figure 1, and grouped by genetic lineage, for the following six MTC lineages: Beijing, *M. bovis*, Central-Asia, East-African-Indian, Haarlem and Latin-American-Mediterranean. These Figures provides the best display of the global phylogeographical structure of the MTC population. Similar results focused in Europe (data not shown) and using SpolDB4 suggests the existence of fine geographic genetic clines between four prevalent genotypes belonging to the modern MTC types, i.e. ST53-T1, ST50-Haarlem3, ST47-Haarlem1, ST42-LAM9 [70].

## Discussion

In this study, we data-mined an updated international spoligotype database of the *M. tuberculosis *complex, SpolDB4, both for improving classification of MTC genomes, and for presenting a more reliable snapshot picture of the global and local population genetics of tubercle bacilli. Considering the known diversity of the origin of patients, SpolDB4 represents clinical isolates from a total of 141 countries. This is to our knowledge the largest collaborative effort to describe the worldwide genetic structure of MTC.

The scaling-up that represents SpolDB4 relatively to SpolDB3 (4×), allowed new sub lineages to be discovered. However, it also showed the limit of the approach of using spoligotyping only to define the precise identity of a given MTC clone, since over fitting was observed. Combined DR, MLVA, SNPs, Region of Differences polymorphic datasets are now required to improve our knowledge of MTC genomes diversity.

Today's observed pattern of phylogeographical diversity of MTC is undoubtedly the result of both a deep ecological differentiation and of a more recent demographic and epidemic history. It is tempting to speculate, especially since the publication of the recent studies done on *M. canettii *[15,71], that TB is as old as humanity. It is also tempting to hypothesize that the EAI ancestral strains spread back from Asia to Africa through India concomitantly to human migrations [72], and that evolution gave rise to the CAS lineage, and possibly to all "modern" TB lineages. Recently, Mokrousov *et al. *used the Beijing lineage as a model to compare its phylogeography with human demography and Y chromosome-based phylogeography [73]. Further work using other genetic markers will help to better define the retrospective demographical history of the various sub lineages described here.

Such an endeavour as the building of SpolDB4, and more generally the building of a representative genetic diversity database, should however assume limitations concerning both the quality and representativity of data. We partially eliminated the first problems by carefully double checking many datasets visually, reinterpreting other datasets by asking for the autoradiography results sent as electronic files, or by simply excluding datasets harbouring systematic genotyping errors. The procedure of examining only STs improves data quality by minimizing artefacts analysis. Some internationally agreed upon recommendations to improve the quality of spoligotyping are also in progress and will be published elsewhere. The second problem (representativity) is more chronic and difficult to solve. More financial means should be devoted to improving both mycobacteriology diagnostic and genotyping facilities in countries where the outbreaks prevail. Another limitation using spoligotyping is the study of mixed populations of bacilli [74]. Indeed, MLVA is appropriate, contrary to spoligotyping, to reveal the existence of dual infections, an issue which, especially in high prevalence countries, was probably underestimated and that may sometimes jeopardize spoligotyping results. Future studies should attempt to evaluate whether admixture models can in fact explain some yet undefined mixed spoligotyping signatures.

One of the major lessons from this collaborative effort is that new markers such as MLVA, SNPs or others are needed to improve our knowledge on the population structure of MTC. MLVA provides an improved knowledge of MTC clonal complexes, a strategy for the MTC which could even be more effective than the Multi-Locus-Sequence-Typing (MLST) approach [55,75]. MLVA allows the investigator to limit the quantity of required genotyping to only type epidemiologically or phylogenetically informative markers, depending on the branch depth (regarding time) to which a given data set should to be analyzed [76]. Rapid changes in MTC genotyping methodologies have lead to numerous ongoing debates about the choice of the best genotyping strategy [77-79].

Our efforts to characterize the classification of the tuberculosis strains present within populations have also led to an increased understanding of their global distribution. Our results do not fully agree with recently published results [11] suggesting that *M. tuberculosis *did not possess a finer geographic structure than the one defined within broad regions (East Asia, Africa, Europe, Philippines and Americas). On the contrary, our results demonstrate that the genetic diversity of *M. tuberculosis *genomes and hence their population structure, is strongly linked to geography at a fine geographical scale, thus reinforcing the importance of localized effort to control tuberculosis and to consider the global tuberculosis pandemia as the sum of very different and genetically separate individual outbreaks. Future work should focus on an adequate modelization of the database according to demography and tuberculosis prevalence, to present a more realistic quantitative TB genetic landscape.

Linking these results for a clinical benefit for individual patients remains another challenge. In particular, we need to better understand why in certain areas a small number of strains are causing a disproportionate number of cases of the disease and we need to better understand the effects of host's genetic and environmental variability on the presentation of the disease [80]. In the Western world TB occurs mainly due to reactivation of disease in the older age class and immigration in younger ones. Consequently the characteristics of the bacterial population are those of an old outbreak under extinction, with the superimposition of new characteristics due to cases of importation and recent transmission. The more homogeneous population structure in high-burden countries is more likely to reflect the ongoing transmission in all age classes. Selection of particular genotypes (e.g. due to vaccination with *M. bovis *BCG) and clonal selection [81] has also been suggested [50,82]. Previous colonization histories, and of course more deeply rooted anthropological structures as well as geographic isolation, may have also contributed to the complex tuberculosis genetic landscape.

## Conclusion

The SpolDB4 database is by far one of the largest publicly available database on *M. tuberculosis *complex genetic polymorphism with a universal nomenclature system of spoligotyping (octal or arbitrary ST number) as well as a global epidemiological information system. Our results suggest the existence of fine geographical genetic clines that may correlate to the passed *Homo sapiens sapiens *demographical history. Further combined (multimarkers) genetic databases, as well as local finer scale analysis are in progress to further analyze the complexity of the evolutionary history of the MTC.

## Methods

### Database

A total of 39,295 entries were collected in an Access^® ^database. The Previous STs numbered 1 to 817 were previously described in SpolDB3. ST633, 714, 729, 770, 797, which were missing in the former version were replaced by new genotypes. SpolDB4 is available [see additional file 1] and can also be downloaded (sorted/unsorted versions) at: [83]. A dedicated interactive website version is available [93].

The distribution by continent and country of isolation is shown below. Macro region name, defining acronym, identification number and total number of patterns (into brackets) are respectively: Africa AFR -1 (n = 3121), Central-America CAM -2 (n = 1353), Europe EUR -3 (n = 13624), Far-East Asia FEA -4 (n = 2624), Middle East and Central Asia MECA -5 (n = 2639), North-America NAM -6 (n = 9153), Oceania OCE -7 (n = 235), South-America SAM -8 (n = 3176).

### Origin of spoligotypes

The spoligotypes were either obtained at the Pasteur Institute of Guadeloupe using the 43-spacers format and home-made membranes [84,85], received from co-investigators and collaborating laboratories, or retrieved from local molecular epidemiological studies or diversity-driven published articles. The sampling is still far from being representative for all countries, and in many cases, limited epidemiological and patient information on the isolates were available. However, we assume that this " convenience sample " is representative of world-wide TB genotypes and that, with spoligotypes from almost 40.000 *M. tuberculosis *isolates, this 4^th ^version allows for new and robust inferences in phylogeography, population genetics, and global epidemiology of *M. tuberculosis *to be drawn.

### Description

The description of SpolDB4, per country of isolation of the clinical isolates, or in very rare cases, by country of origin of the patients, is as follows.

AFR, (n = 3121): Angola (n = 4), Burundi (n = 18), Benin (n = 4), Botswana (n = 1), Burkina Faso (n = 1), Centrafrican republic (n = 154), Ivory Coast (n = 84), Cameroon (n = 498), Congo Democratic (former Zaire) (n = 7), Congo (n = 20), Djibouti (n = 5), Algeria (n = 137), Egypt (n = 60), Eritrea (n = 8), Ethiopia (n = 153), Gabon (n = 2), Ghana (n = 1), Guinea (n = 7), Gambia (n = 1), Guinea-Bissau (n = 217), Kenya (n = 71), Libya (n = 54), Morocco (n = 127), Mali (n = 5), Mozambique (n = 28), Mauritania (n = 4), Malawi (n = 122), Namibia (n = 79), Niger (n = 1), Nigeria (n = 6), Rwanda (n = 8), Sudan (n = 41), Senegal (n = 69), Sierra Leone (n = 3), Somalia (n = 226), Tunisia (n = 10), Tanzania (n = 9), Uganda (n = 11), South Africa (n = 564), Zambia (n = 43), Zimbabwe (n = 247), East-Africa (not specified, n = 15).

CAM, (n = 1353): Netherlands Antilles (n = 1), Barbados (n = 6), Costa Rica (n = 1) Cuba (n = 239), Commonwealth of Dominica (n = 1), Guadeloupe (n = 232), Honduras (n = 7), Haiti (n = 375), Mexico (n = 386), Martinique (n = 105).

EUR, (n = 13624): Albania (n = 5), Austria (n = 1456), Belgium (n = 517), Bulgaria (n = 2), Bosnia and Herzegovina (n = 3), Czech Republic (n = 393), Germany (n = 574), Denmark (n = 281), Spain (n = 374), Estonia (n = 115), Finland (n = 347), France (n = 2265), United-Kingdom (n = 1523), Greece (n = 1), Hungary (n = 62), Ireland (n = 1559*, mainly *M. bovis*), Italy (n = 934), Sicily (n = 125), Latvia (n = 138), Liechtenstein (n = 1), Lithuania (n = 3), Luxembourg (n = 1), Moldova (n = 1), Macedonia (n = 7), Netherlands (n = 973), Norway (n = 31), Poland (n = 227), Portugal (n = 336), Romania (n = 26), Russia (n = 986), Sweden (n = 328), Swiss (n = 1), Ukraine (n = 4), Yugoslavia (n = 28).

FEA, (n = 2624): China (n = 145), Indonesia (n = 344), Japan (n = 138), Korea (n = 4), Myanmar (n = 20), Mongolia (n = 19), Malaysia (n = 598), Philippines (n = 237), Singapore (n = 4), Thailand (n = 302), Vietnam (n = 789), Far-east-Asia unspecified (n = 24).

MECA, (n = 2639): Afghanistan (n = 3), Armenia (n = 119) Azerbaijan (n = 71), Bangladesh (n = 676), Comoro Islands (n = 14), Georgia (n = 272), India (n = 483), Iran (n = 110), Iraq (n = 5), Israel (n = 15), Kazakhstan (n = 55), Lebanon (n = 2), Sri Lanka (n = 16), Madagascar (n = 395), Mauritius (n = 21), Nepal (n = 6), Pakistan (n = 90), Reunion Island (n = 16), Saudi Arabia (n = 99), Turkey (n = 170), Yemen (n = 1).

NAM, (n = 9153): Canada (n = 266), Greenland (n = 4), USA unspecified (n = 1690), USA (Alabama, n = 3), USA (New-York, n = 5948), USA (Texas, n = 1242).

OCE, (n = 235): Australia (n = 36), New Zealand (n = 151), French Polynesia (n = 2), USA (Hawaii, n = 46).

SAM, (n = 3176): Argentina (n = 1150*, mainly *M. bovis*), Bolivia (n = 4), Brazil (n = 842), Chile (n = 2), Colombia (n = 1), Ecuador (n = 12), French Guiana (n = 375), Guiana (n = 3), Peru (n = 96), Paraguay (n = 6), Suriname (n = 8), Uruguay (n = 5), Venezuela (n = 672).

### Data format

All spoligotypes were converted into the octal format within Excel spreadsheets [86]. The database is maintained under an Access^® ^format, whereas a Bionumerics^® ^version is also regularly updated (Applied Maths, Sint-Marteen-Latem, Belgium). An updated mySQL-Java-based version is in development. The Information system automatically attributes the shared-type (ST) number to all the entries that correspond to an identical spoligotype found in two or more individual patient isolates, whereas, the entries occurring only once are considered as orphan.

### Combined automatized-expert based classification of spoligotypes

To be classified by SpolNet, spoligotypes must be expressed under the form of binary vectors of 43 bits. Using clade and Principal Genetic Groups (PGG) clustering [87], which was previously established or collected by our laboratory and others [88], computerized rules have been generated to sort spoligotypes into clades. Each computerized rule is a translation of a global visual recognition rule. This rule may be defined using a combination of four criteria: (a) presence of a block of one or many consecutive bits (b) absence of a block of one or many consecutive bits (c) presence of at least one bit in a given bit interval (d) absence of at least one bit in a given bit interval. The computer science translation of these four criteria required the creation of a positive and a negative rule. The positive rule translates the fact that a bit is absent or present in the vector. The negative rule translates the fact that there must be at least one bit present or absent in a given interval. Three values can be attributed for each bit, "n" = present, "o" = absent, "x" = variable. The software generates a text file, which contains all spoligotypes sorted by family and PGG, and a specific file for each rule containing the spoligotypes which fulfilled the selection criteria. Rules are hierarchical, i.e. some rules are smooth and almost each spoligotype fulfils it (ex: T1 rule), whereas others are tight (ex: LAM12-Madrid1), hence a final multilevel classification scheme for each shared-type from the most precise to the less precise sub lineage/lineage label (ex: LAM3/LAM9/T1).

Secondly, an algorithm establishes the hierarchic links from the entry data file. This algorithm is based on a comparison of vector. The model (assumption) relies on an evolution that proceeds by deletion of a unique block of one to *n *consecutive bits on the DR locus. For a given spoligotype, the algorithm finds all the potential offspring spoligotypes. The result is transferred into a file whose format is directly used by the BioLayout software [89]. Building a file for BioLayout looks like a simple task but it turns out to be a tedious and time-consuming task for file with hundreds of spoligotypes. This file cannot be built without software for thousands of spoligotypes, as it is the case for the SpolDB4 database project. The two developed algorithms are exponential, i.e. the time to sort data files and to generate Biolayout files increases with the size of the entry data file. As an example, a file with 1000 spoligotypes may take approximately 20 minutes to be sorted and 20 minutes to generate the Biolayout file. New spoligotype-recognition rules may be introduced as a dynamic process, either when new genotypes are discovered, to introduce new hypothesis, or to modify pre-existing rules.

### Definition of indices

The global distribution of spoligotyping patterns was assessed within and between the eight studied continental regions: Africa, Central America, Europe, Far East Asia, Middle East and Central Asia, North America, Oceania, and South America. A slight modification in definition of C1 and C2 was introduced, i.e. a spreading Index SI ≥ 25 instead of SI ≥ 30 is now required to define a clone as "epidemic". Briefly, the Spreading Index (SI) represents a mean number of occurences of a clone, independent of the setting (total n° of occurrences for this ST divided by the number of geographical areas where it is found). The reader should refer to Figure 2 of [26], or to Table 1 for full definition of C1 and C2.

### Availability

SpolDB4 listing [see additional file 1] is available as supplemental material. A dedicated interactive website, SITVIT1 which will allow SpolDB4 (39525 genotypes) to be queried online is available [93]. In its research format SITVIT1 allows one to enter MLVA data and to automatically detect MLVA clusters [90]. The full list of investigators having contributed to SpolDBs since the origin of the project will also appear on the website. SpotClust results were extracted from [91].

### Quality control (QC)

During the SpolDB4 project, we faced increased quality control problems due to an increased recruitment rate and had to exclude some datasets. A common problem was the systematic absence of one spacer on some membranes. These data sets were systematically excluded. Most of these problems were linked to manufacturing defaults in the commercialized membranes. All data sets were checked individually and sometimes audited on source results (autoradiography). Most investigators, to check their procedures, completed a QC form. International Guidelines to increase spoligotyping quality are in progress and will be reported elsewhere. As in all databases, we assume a reasonable (2 to 5% maximum) error rate in data points. However, the problem of QC in high throughput genotyping technique and database science technology is an emerging issue [92]. A list of STs whose distribution did not change between SpolDB3 and 4 is available upon request. These genotypes represent: (1) ongoing genotypes not detected because of no follow-up in a given area, (2) potentially extinct genotypes, (3) potential typing artefacts. Similarly as in SpolDB3, where 5 STs had been suppressed, 22 STs in SpolDB4 could be artefacts (ST422, 424, 425, 454, 456, 540, 547, 551, 553, 556, 571, 870, 886, 887, 900, 901, 908, 1270, 1575, 1608, 1625, 1896).

## Authors' contributions

All authors contributed to the spoligotyping data contained in the database at various levels, by locally isolating, identifying clinical isolates, preparing DNA and genotyping clinically isolates of MTC and sending their results to the Pasteur Institute in Guadeloupe. Their relative contribution would be to tedious to mention here, however, they are all part of the paper because such population-based studies could not be done without adequate mycobacteriological diagnostics, i.e., isolation of clinical isolates, identification, drug-susceptibility testing, DNA extraction, genotyping and ultimately by sharing of data, hence without adequate reward of the numerous persons and labs involved in such analysis.

KB managed the SpolDB4 Information System, controlled data quality, did part of the supervised analysis, used SpolNet for Bioinformatical classification, compiled data synthesis. CS initiated the SpolDB project, established many of the contacts, recruited many of the investigators with NR, writing e-mails, checked the Information System developments helped by numerous Computer scientists (Philippe Leremon, Christel Delfino, Philippe Abdoul, Georges Valétudie, are warmly acknowledged). CS did part of the mining with KB and wrote the paper. JRD managed the PHRI New-York database and sent his data regularly to CS and KB. LR and WP were the two most "big account" data providers, together with AG. BL produced the maps (P. Waniez, Philcarto^®^, IRD, France) and contributed to the statistical analysis.

## Supplementary Material

Additional file 1 **Supplemental Table: **SpolDB4 listing of all STs, binary description, octal description, distribution per country of isolation and/or of origin when available, clade/subclade label. Country names were chosen according to the ISO3166-three-letter format. "U" = unknown. Clade/subclade label using spoligotyping only should be taken as presumptive or indicative of a likely clade/subclade belonging but may in some case be misleading and requires in most cases further investigations to confirm the identity of a given isolate. In some instances, mixed patterns (unrecognized) did not unambiguously allow spoligotyping classification, hence an ambiguous final label in this table. Click here for file

## Figures and Tables

**Table 1:** **A**. Definition of the variables (MC, Ar, SI) used in SpolDB4 to define : (1) the geographic index C1 (Endemic, Localized, Ubiquitous) (2) the quantitative index C2 (Epidemic, Common, Recurrent, Rare). **B**. Distribution of the 1939 Shared-types in 12 classes.

Name	Abbreviation		Type of data, Def.	Rules for Definition of Qualifiers (C1 and C2)
**A Definitions**				
Matching Code	MC		1–8 digits, built by linking region codes	If 1 digit, then C1 = Endemic (genotype found in one macroregion only) If 2 digits, then C1 = Localized (genotype found in two macroregions) If ≥ 3 digits, go to Area section below for further interpretation
Area	Ar		numeric, n° of countries in which a given SIT is found	If MC ≥ 3 digits and Areas ≤ 5; C1 = Localized If MC ≥ 3 digits and Areas ≥ 6; C1 = Ubiquitous (genotype found in more than three macroregions)
Spreading Index	SI		numeric, mean indicator of spreading independent of geography SI = n/Ar where n is the absolute value for a given shared-type	If SI ≥ 25; C2 = Epidemic If 10 < SI < 24; C2 = Common If 3 < SI < 9; C2 = Recurrent If SI ≤ 2; C2 = Rare
**B Distribution of the 1939 genotypes in 12 class**				
Endemic Epidemic		6	Endemic Recurrent	286
Localized Epidemic		2	Localized Recurrent	207
Ubiquitous Epidemic		6	Ubiquitous Recurrent	176
Endemic Common		31	Endemic Rare	501
Localized Common		14	Localized Rare	587
Ubiquitous Common		20	Ubiquitous Rare	82
NA*		21	Total	1939
*not applicable				

**Table 2:** Analysis of the geographical Inter and Intra-continental matches between the shared-types found within 1 or 2 settings (endemic types n = 824) and localized types (n = 564), within and between the 8 macro-regions. The geographical analysis of the matches between localized types found in 3 macroregions and 5 settings or more (n = 246) and between the ubiquitous types (found in more than 3 regions (n = 284) was not done. Number of intra and intercontinental matches between STs detected between the 8 macro regions previously defined.

**Macroregion (code)**	**Africa**	**Americas**			**Europe**	**Asia**		**Oceania**
					
		**North America**	**Central America/Caribbean**	**South America**		**Middle-East and Central-Asia**	**Far-East Asia**	
**Africa (1)**	73	29	2	7	79	7	3	0
**North America (2)**		138	8	35	131	34	45	4
**Central America (3)**			7	0	9	1	2	0
**South America (4)**				94	69	ND	0	3
**Europe (5)**					351	56	25	3
**Middle-East Asia (6)**						99	8	0
**Far-East Asia (7)**							61	4
**Oceania (8)**								1

ND = not done
