# Supplementary material for: Mycobacterium tuberculosis complex genetic diversity: mining the fourth international spoligotyping database (SpolDB4) for classification, population genetics and epidemiology
Source: BMC Microbiol. 2006 Mar 6;6:23. doi: 10.1186/1471-2180-6-23 (PMC1468417; doi:10.1186/1471-2180-6-23)
Supplement: Additional file 1 — Supplemental Table: SpolDB4 listing of all STs, binary description, octal description, distribution per country of isolation and/or of origin when available, clade/subclade label. Country names were chosen according to the ISO3166-three-letter format. "U" = unknown. Clade/subclade label using spoligotyping only should be taken as presumptive or indicative of a likely clade/subclade belonging but may in some case be misleading and requires in most cases further investigations to confirm the identity of a given isolate. In some instances, mixed patterns (unrecognized) did not unambiguously allow spoligotyping classification, hence an ambiguous final label in this table. [file 1471-2180-6-23-S1.pdf]

Type	Spoligotype Description	Total	Geographical Distribution	label
1	□□□□□□□□□□□□□□□□□□□□□□□□□□□□□□□□□□■■■■■■■■■	3758	?(1) ARG(3) ARM(49) AUS(41) AUT(25) AZE(52) BEL(13) BGD(121) CAN(4) CHN(46) CUB(26) CZE(14) DEU(19) DNK(18) DZA(1) EST(50) FIN(4) FXX(35) GBR(10) GEO(58) GLP(1) GNB(1) GUF(6) HTI(1) IDN(147) IND(31) IRN(10) ISR(10) ITA(21) JPN(100) KAZ(38) KEN(6) KOR(1) LBY(3) LVA(76) MAR(1) MDG(20) MEX(1) MNG(10) MOZ(2) MUS(10) MWI(1) MYS(240) NLD(22) NZL(4) PAK(5) PER(4) PHL(12) POL(7) PRT(4) REU(6) RUS(426) SAU(5) SEN(8) SGP(2) SUR(1) SWE(32) THA(119) USA(1277) VNM(387) ZAF(106) ZWE(4)	BEIJING
2	□□□□□□□□□□□□□□□□□□□□□□□□■□□□□□□■□□□□■■■■■■■	283	ARG(36) AUT(15) BEL(5) BRA(4) CUB(21) CZE(7) DEU(2) DNK(3) DZA(2) ESP(2) FIN(6) FXX(11) GLP(12) GUF(19) HTI(37) IND(1) ITA(1) LBY(1) MEX(9) MTQ(2) NLD(3) POL(4) RUS(2) TUR(3) USA(74) ZAF(1)	H2
3	□□□□□□□□□□□□□□□□□□□□□□□□■■■■■■□■□□□□■■■■■■■	66	ARG(2) AUT(6) BRA(2) CUB(3) FXX(3) GLP(3) HTI(1) ITA(1) MEX(9) NLD(3) POL(1) SAU(1) SUR(1) USA(29) VEN(1)	H3
4	□□□□□□□□□□□□□□□□□□□□□□□□■■■■■■■■□□□□■■■■■■■	118	ARG(6) AUT(7) BEL(4) CAN(11) DEU(1) FIN(1) FXX(6) GBR(1) GLP(1) GNB(1) GUF(2) HTI(3) ITA(13) ITAS(2) KEN(1) LBY(2) MAR(1) MTQ(1) MWI(1) MYS(2) NLD(7) NOR(1) POL(1) PRT(3) RUS(2) SAU(1) SWE(2) TUR(4) USA (21) VEN(4) ZAF(5)	LAM3 and S /convergent
5	□□□□□□□□□■■□■■■■■■■■■■■■■■■■■■■■□□□□■■■■■■■	25	GLP(1) GUF(2) HTI(7) MTQ(2) USA (13)	T1
6	□□□■■■■■■■■■■■■■■■■■■■■■■■■■□□□□■□■■■■■□■■■	39	AUT(3) DNK(13) GBR(5) MYS(1) NLD(7) NZL(2) SWE(4) USA (4)	EAI1_SOM
7	□■■■■■■■■■■■■■■■■■■■■■■■■■■■■■■■□□□□■■■■■■■	32	AUT(2) CMR(1) DEU(1) ESP(1) FXX(1) GBR(2) GLP(3) GUF(3) HTI(2) JPN(1) MEX(1) MYS(1) NLD(3) PER(1) TUR(1) USA (8)	T1
8	■□□□□□□□□□□□□■■■■■■■■■■■■■■■□□□□■□■■■■■■■■■	63	COM(1) DNK(16) GBR(10) IND(2) MYS(1) NLD(2) NOR(2) SWE(1) TUN(2) USA(21) ZMB(5)	EAI5 or EAI3
9	■□□□□□□□■■■■■■■■■□■■■■■■■■■■■■■■□□□□■■□□□□■	4	GBR(1) NLD(2) USA (1)	X2
10	■□□■■■■■■■■■■■■■■■□■□■■■■■■■□□□□■□■■■■■■■■■	61	BEL(4) DNK(18) FXX(3) GBR(11) ITA(1) MDG(1) MWI(2) NLD(19) SWE(2)	EAI5 or EAI3
11	■□□■■■■■■■■■■■■■■■■■■■■■■■■■□□□□■□■■□□□■■■■	237	AUS(1) AUT(4) BEL(2) BGD(14) CUB(1) DEU(2) DNK(17) FXX(2) GBR(39) GLP(1) GUF(4) IDN(1) IND(34) IRN(1) MYS(17) NLD(20) NZL(6) PAK(2) SWE(1) USA(68)	EAI3_IND
12	■□■□□□□□□□□□□■■■■□■■■■■■■■■■■■■■□□□□■■■■■■■	3	GLP(2) USA (1)	X3
13	■□■□□□□□□□□□■■□■■□■■■■■■■■■■■■■■□□□□■■■■■■■	2	GLP(2)	X3
14	■□■□□□□□□□□□■■■■■□■■■■■■■■■■■■■■□□□□■■■■■■■	33	GLP(25) GUF(3) MTQ(1) USA(3) VEN(1)	X3
15	■□■■■■■■■■■■■■■■■■■■□□□□□□□□■■■■□□□□■■■■■■■	2	GLP(2)	T1
16	■□■■■■■■■■■■■■■■■■■■■■■■■■■■□□□□■□■■□□□■■■■	4	DNK(2) GBR(2)	EAI5 or EAI3
17	■■□■■■■■■■■■□■■■■■■■□□□□■■■■■■■■□□□□■■■■■■■	322	ARG(3) BRA(33) CMR(2) CUB(1) CZE(3) ESP(17) FXX(1) GLP(11) GUF(7) HTI(22) MEX(3) MTQ(2) MYS(1) NAM(1) NLD(2) PHL(3) PRT(6) USA(76) VEN(128)	LAM2
18	■■□■■■■■■■■■■■■■■□■■■■■■■■■■■■■■□□□□■■□□□□■	14	BRA(5) GBR(5) NLD(2) USA (2)	X2
19	■■□■■■■■■■■■■■■■■■■□□■■■■■■■□□□□■□■■■■■■■■■	430	AUS(11) AUT(13) BEL(1) FIN(1) FXX(2) GBR(1) GLP(2) IDN(1) ITA(20) JPN(1) MYS(43) NLD(3) PHL(132) SUR(1) SWE(4) THA(8) USA(183) VNM(3)	EAI2_MANILLA
20	■■□■■■■■■■■■■■■■■■■■□□□□■■■■■■■■□□□□■■■■■■■	426	ARG(2) AUS(2) AUT(1) BEL(8) BRA(26) CMR(7) CUB(5) DEU(10) ECU(1) ESP(7) EST(4) FIN(1) FXX(18) GBR(1) GEO(1) GLP(3) GNB(3) GUF(10) HTI(26) IDN(1) LBY(1) MEX(7) MOZ(1) MWI(11) NAM(62) NLD(11) PER(1) PHL(2) PRT(49) RUS(3) SWE(3) USA(108) VEN(26) VNM(1) ZAF(1) ZWE(2)	LAM1
21	■■■□□□□■■□■■■■■■■■■□□□□□□□□□□□□□□□□■■■■■■■■	145	AUT(1) BEL(1) COM(3) DNK(2) FIN(2) FXX(6) GBR(9) ITA(1) KEN(17) MDG(37) MOZ(1) MUS(1) MWI(3) NLD(12) SDN(1) SWE(3) TZA(1) USA(18)n ZAF(1) ZMB(22) ZWE(3)	CAS1_KILI
22	■■■□□□□■■■■■■■■■■■■□□□□□□□□□□□□□□□□■■■■■■■■	24	BGD(2) EGY(1) FIN(2) FXX(1) GBR(1) IRN(5) NLD(2) SAU(7) ZMB(3)	CAS
23	■■■□□□□■■■■■■■■■■■■■■■□□□□□□□□□□□□□■■□□■■■■	3	GBR(3)	CAS
24	■■■□□□□■■■■■■■■■■■■■■■□□□□□□□□□□□□■■□□□□■■■	6	GBR(1) NLD(2) SDN(2) SWE(1)	CAS1_DELHI
25	■■■□□□□■■■■■■■■■■■■■■■□□□□□□□□□□□□■■□□■■■■■	128	AUS(2) AUT(1) BEL(1) BGD(4) CAF(1) CZE(2) EGY(2) EST(1) ETH(1) FXX(5) GBR(10) GEO(1) IDN(2) IND(1) IRN(5) ITA(3) LBY(9) MUS(1) NLD(13) PAK(2) SAU(4) SDN(14) SEN(1) SWE(5) USA(35) ZAF(1) ZWE(1)	CAS1_DELHI
26	■■■□□□□■■■■■■■■■■■■■■■□□□□□□□□□□□□■■■■■■■■■	404	AUS(9) AUT(9) BEL(4) BGD(54) COM(1) DEU(6) DNK(1) FIN(1) FXX(7) GBR(15) GEO(1) GLP(1) IND(48) IRN(7) ITA(7) KEN(9) MUS(2) MYS(4) NLD(37) NZL(2)	CAS1_DELHI

			PAK(26) PRT(2) SAU(2) SDN(3) SWE(10) USA(135) ZAF(1)	
27	■■■□□□□■■■■■■■■■■■■■■■□□■■■■■■■■□□□□■■■■■■■	11	AUS(1) DEU(2) GBR(3) KEN(1) PAK(1) SAU(1) USA(2)	U(CAS_ANCESTOR?)
28	■■■□■■■■■■■■■■■■■■■■■■■■■■■■□□□□■□■■□■■■■■■	5	MYS(1) NLD(4)	EAI5
29	■■■■■□□□□□□□□□□□□■■□□□□□□□□□□□□□□□□□□■■■■■	29	ESP(1) FXX(6) GLP(5) PRT(1) USA (16)	U (likely LAM)
30	■■■■■■□■■■■■■■■■■□■■□□□□■■■■■■■■□□□□■■■■■■■	3	GLP(2) USA(1)	LAM9
31	■■■■■■■□□□□□□■■■■■■■■■■■■■■■■■■■□□□□■■■□□□□	40	AUS(1) AUT(1) CUB(1) CZE(13) FIN(1) FXX(6) GLP(2) GUF(3) ITA(4) MDG(3) MTQ(1) NLD(1) USA (3)	T1
32	■■■■■■■■□□□□□□□□□□□□□□□□□□□□□□□□□□□□□□■■■■■	65	BEL(31) BRA(1) DEU(1) FXX(1) GUF(2) IDN(10) ITA(3) MYS(2) NLD(7) NZL(2) SWE(1) USA (2) ZMB(2)	U (likely S)
33	■■■■■■■■□□□■■■■■■■■■□□□□■■■■■■■■□□□□■■■■■■■	342	ARG(60) AUT(5) BEL(6) BRA(17) CHL(1) CUB(15) ESP(18) FIN(1) FXX(27) GBR(3) GUF(3) HND(1) HTI(1) ITA(13) ITAS(5) LBY(2) MDG(3) MEX(1) MOZ(2) NLD(5) PER(2) PRT(7) SWE(1) USA(89) VEN(4) ZAF(50)	LAM3
34	■■■■■■■■□□■■■■■■■■■■■■■■■■■■■■■■□□□□■■■■■■■	362	ARG(6) AUS(2) AUT(16) BEL(1) BRA(7) CAN(75) CUB(1) DEU(3) DZA(6) EGY(5) FIN(2) FXX(13) GBR(2) GEO(3) GLP(2) GUF(5) HTI(7) IDN(3) IND(1) ITA(37) ITAS(10) KEN(3) LBY(2) MAR(1) MDG(3) MEX(1) MOZ(2) MWI(1) NLD(3) NZL(2) PRT(8) REU(1) SAU(3) SWE(3) THA(1) USA(92) VEN(8) VNM(2) ZAF(19)	S
35	■■■■■■■■■■■■□■■■■■■■■■■■■■■■□□□■□□□□■■■■■■■	74	ARG(1) ARM(3) AUT(13) AZE(3) BEL(1) CHN(1) CZE(3) DEU(2) ESP(1) EST(2) FIN(4) GBR(2) GLP(1) IRN(2) JPN(2) KAZ(3) LVA(1) MEX(1) NLD(1) POL(2) RUS(18) USA (6) ZAF(1)	H4
36	■■■■■■■■■■■■□■■■■■■■■■■■■■■■■■□■□□□□■■■■■■■	43	ARG(1) AUS(3) AUT(4) BRA(1) CZE(2) DEU(5) FIN(2) FXX(3) GUF(1) ITA(2) LBY(4) MTQ(1) NLD(4) POL(1) SAU(1) TUN(1) USA (5) VEN(1) VNM(1)	H3-T3
37	■■■■■■■■■■■■□■■■■■■■■■■■■■■■■■■■□□□□■■■■■■■	155	AUS(2) AUT(12) BEL(3) BRA(3) CAN(5) CHN(2) CIV(1) CUB(5) CZE(8) DEU(4) DNK(17) DZA(1) EST(1) ETH(1) FIN(5) FXX(8) GBR(1) GEO(1) GLP(1) GUF(1) HTI(1) IDN(1) IRN(4) ITA(2) ITAS(1) LBY(1) LVA(1) MDG(3) NLD(11) NZL(2) PAK(1) POL(4) SAU(2) SWE(6) THA(1) TUR(1) USA(26) VEN(1) ZWE(4)	T3
38	■■■■■■■■■■■■■■■■■□■□□□□□■■■■■■■■□□□□■■□□□□■	125	GBR(3) IND(3) USA (119)	X2
39	■■■■■■■■■■■■■■■■■■□■■■□□■■■■■■■■□□□□■□□■■■■	67	ARM(2) AUT(21) BEL(1) CZE(1) DEU(6) FXX(1) GBR(1) ITA(1) NLD(2) POL(5) USA (7) VNM(1) ZAF(18)	T4-CEU1
40	■■■■■■■■■■■■■■■■■■□■■■■■■■■■■■■■■□□□□■■■■■■■	87	AUT(14) BEL(1) CZE(7) DEU(5) DNK(4) ESP(1) ETH(2) FIN(4) FXX(10) GBR(2) GEO(1) GLP(4) IRN(1) ITA(1) ITAS(1) LBY(1) MDG(1) NLD(2) POL(1) RUS(4) USA(20)	T4
41	■■■■■■■■■■■■■■■■■■■□□□□□■□□■■■■■□□□□■■■■■■■	127	AUT(5) BEL(1) CZE(2) DEU(6) DNK(7) EGY(2) ETH(7) FIN(2) FXX(11) GBR(6) GUF(1) ITA(1) NLD(28) NOR(4) NZL(2) POL(1) ROM(1) SAU(1) SWE(2) TUR(33) USA(4)	LAM7_TUR
42	■■■■■■■■■■■■■■■■■■■■□□□□■■■■■■■■□□□□■■■■■■■	1227	ARG(69) ARM(4) AUS(3) AUT(25) AZE(4) BEL(25) BGD(28) BRA(54) CAF(3) CMR(1) CUB(19) CZE(4) DEU(6) DZA(11) EGY(1) ESP(14) EST(5) ETH(3) FIN(25) FXX(45) GBR(5) GEO(7) GLP(11) GNB(22) GUF(15) HTI(27) IDN(1) IND(3) IRN(2) ITA(77) ITAS(17) KAZ(4) KEN(8) LBY(2) LVA(19) MAR(27) MDG(4) MEX(25) MOZ(2) MTQ(3) MWI(5) MYS(1) NLD(32) NZL(26) PER(7) PHL(5) POL(6) PRT(44) PYF(1) RUS(68) SAU(2) SEN(2) SWE(12) THA(1) TUN(1) TUR(1) USA (259) VEN(90) VNM(1) ZAF(7) ZWE(26)	LAM9
43	■■■■■■■■■■■■■■■■■■■■■■□□■■■■□□□□■□■■■■■■■■■	17	DEU(3) GBR(3) ITA(1) MYS(1) NLD(3) SWE(2) USA(3) VNM(1)	EAI6_BGD1
44	■■■■■■■■■■■■■■■■■■■■■■□■■■■■■■■■□□□□■■■■■■■	112	ARG(3) AUS(1) AUT(28) BEL(1) CMR(1) CZE(8) DEU(2) DNK(1) EGY(1) ESP(1) EST(1) FIN(2) FXX(3) GBR(2) GLP(3) GUF(3) IDN(1) IND(1) ITA(14) ITAS(2) NLD(3) POL(3) PRT(4) USA (15) VEN(4) ZAF(3) ZWE(1)	T5
45	■■■■■■■■■■■■■■■■■■■■■■■□■□□□□□□■□□□□■■■■■■■	63	AUT(18) BRA(6) CZE(1) DEU(4) DNK(2) ESP(1) GBR(1) GLP(5) HTI(1) ITA(3) MDG(1) MNG(1) MTQ(7) MYS(1) NLD(2) PRT(1) RUS(2) USA (6)	H1
46	■■■■■■■■■■■■■■■■■■■■■■■■□□□□□□□□□□□□□□□□□□□	95	ARG(5) AUS(1) AUT(6) CMR(1) CZE(13) DEU(2) DNK(1) EGY(1) FIN(9) FXX(5) GLP(1) GUF(1) IDN(10) IND(1) ITA(1) MDG(1) MEX(3) MTQ(4) NLD(1) POL(6) SWE(1) TUR(2) USA(19)	U (likely H)
47	■■■■■■■■■■■■■■■■■■■■■■■■■□□□□□□■□□□□■■■■■■■	721	?(1) ARG(10) ARM(4) AUS(3) AUT(126) AZE(1) BEL(12) BGD(1) BRA(23) CAF(37) CAN(5) CMR(3) CUB(12) CZE(38) DEU(24) DNK(1) ESP(11) EST(4) FIN(29) FXX(33) GBR(5) GLP(3) GNB(8) GUF(3) HTI(4) HUN(1) IND(1) ITA(51) ITAS(7) MAR(3) MDG(9) MEX(10) NLD(36) POL(16) PRT(19) ROM(1) RUS(6) SAU(1) SEN(6) SWE(9)	H1

			TUR(1) USA(135) VEN(7) ZWE(1)	
48	■■■■■■■■■■■■■■■■■■■■■■■■■■■■□□□□■□■■■■■□■■■	243	AUS(2) AUT(2) BEL(1) BGD(41) DNK(59) FXX(6) GBR(20) GUF(2) IDN(6) IND(5) ITAS(1) JPN(1) KEN(2) MOZ(4) MYS(9) NLD(44) NOR(9) PAK(1) SAU(1) SWE(10) THA(3) USA(10) VNM(3) ZAF(1)	EAI1_SOM
49	■■■■■■■■■■■■■■■■■■■■■■■■■■■■■■□■□□□□■■■□■■■	102	ARG(5) AUS(1) AUT(7) BEL(1) CAF(3) CHN(1) CMR(5) DEU(1) DZA(1) FIN(23) FXX(7) HUN(4) ITA(5) MEX(1) MTQ(5) MYS(1) NLD(1) PRT(7) SWE(3) USA(19) VEN(1)	H3
50	■■■■■■■■■■■■■■■■■■■■■■■■■■■■■■□■□□□□■■■■■■■	1504	ARG(32) ARM(2) AUS(6) AUT(206) BEL(27) BOL(4) BRA(31) CAF(2) CHN(1) CIV(3) CMR(48) COM(1) CUB(22) CZE(83) DEU(49) DNK(2) DZA(9) EAFR(2) ESP(28) EST(2) FIN(28) FXX(67) GBR(7) GEO(2) GLP(17) GNB(8) GUF(58) HTI(37) HUN(17) IDN(8) IND(5) ITA(94) ITAS(8) JPN(4) KEN(1) LBY(3) MAR(1) MDG(17) MEX(12) MTQ(11) MYS(7) NLD(34) NZL(2) PAK(1) PER(4) POL(19) PRT(10) REU(1) ROM(1) RUS(10) SDN(3) SEN(3) SWE(17) THA(1) TUR(8) USA (401) VEN(10) VNM(5) ZAF(5)	H3
51	■■■■■■■■■■■■■■■■■■■■■■■■■■■■■■■■□□□□■■■□□□□	158	ARG(4) AUS(2) AUT(24) BEL(2) BRA(4) CHN(1) FXX(3) GBR(2) GLP(7) GUF(8) HTI(17) ITA(13) ITAS(2) JPN(1) MDG(1) MEX(2) MYS(19) NLD(1) PRT(5) SWE(1) TUR(1) USA(31) VEN(6) VNM(1)	T1
52	■■■■■■■■■■■■■■■■■■■■■■■■■■■■■■■■□□□□■■■□■■■	393	ARM(10) AUS(2) AUT(23) BDI(1) BEL(23) BGD(1) BRA(1) CAF(10) CAN(4) CHN(2) CIV(1) CMR(24) CUB(1) CZE(17) DEU(7) DNK(6) DZA(2) ESP(2) ETH(1) FIN(4) FXX(40) GBR(7) GLP(1) GUF(1) HTI(2) HUN(6) IDN(2) IND(3) IRN(1) ITA(14) ITAS(3) JPN(4) KEN(4) MDG(7) MEX(4) MTQ(3) MYS(7) NLD(20) NOR(3) NZL(2) PAK(2) POL(6) ROM(1) RUS(5) SAU(1) SWE(13) THA(6) TUR(1) UGA(3) USA(76) VEN(2) ZAF(1)	T2
53	■■■■■■■■■■■■■■■■■■■■■■■■■■■■■■■■□□□□■■■■■■■	2497	ANT(1) ARG(69) ARM(7) AUS(17) AUT(215) AZE(1) BEL(48) BGD(24) BRA(74) BRB(2) CAF(3) CAN(16) CHN(11) CIV(48) CMR(21) COM(1) CUB(23) CZE(30) DEU(55) DNK(41) DZA(4) EAFR(1) EGY(13) ESP(24) EST(9) ETH(37) FIN(49) FXX(150) GBR(58) GEO(12) GLP(25) GNB(8) GUF(49) HTI(27) HUN(8) IDN(16) IND(24) IRN(5) ITA(125) ITAS(13) JPN(6) KAZ(2) KEN(2) LBY(5) LVA(5) MAR(12) MDG(23) MEX(54) MNG(1) MOZ(2) MTQ(15) MWI(12) MYS(14) NAM(7) NLD(84) NOR(4) NZL(14) PAK(4) PER(6) POL(34) PRT(27) REU(1) ROM(1) RUS(26) SAU(4) SDN(1) SEN(21) SUR(2) SWE(30) THA(11) TUR(31) USA(1089) VEN(42) VNM(14) ZAF(14) ZMB(2) ZWE(27)	T1
54	■■■■■■■■■■■■■■■■■■■■■■■■■■■■■■■■□□■■■■■■■■■	78	ARM(1) AUS(1) BRA(2) CUB(1) EGY(1) FXX(2) GBR(3) GEO(2) GNB(2) IND(18) IRN(1) ITA(1) LVA(1) MDG(3) NLD(2) POL(2) RUS(10) SDN(2) SEN(1) THA(1) USA(18) ZAF(3)	MANU2
55	■■■■■■■■□□■■■■■■■■■■■■■■■□■■□■■□□□□□■■■■■■■	2	FXX(2)	U (likely S)
56	■■■■■■■■■■■■□■■■■■■■■■■□□□□□□□□□□□□□□□□□□□	13	BEL(1) FIN(3) FXX(7) GLP(1) USA(1)	U (likely T3)
57	■■■■■■■■■■■■■■■■■■□□■■□□□■■■■■■■□□□□■■■■■■■	4	FXX(2) USA(2)	LAM10_CAM
58	■■■■■■■■■■■■■■■■■■■□■■□■■■■■■■■■■□□□□■■■■■■■	95	ARG(8) AUT(2) BEL(1) BRA(9) CUB(5) DZA(1) ESP(11) FXX(4) GUF(1) ITA(1) MEX(1) NLD(4) PRT(2) USA(41) VEN(4)	T5_MAD2
59	■■■■■■■■■■■■■■■■■■■■□□□□■■□□□□■■□□□□■■■■■■■	133	AUT(1) BEL(2) BRA(1) COM(1) DNK(3) FXX(2) GBR(1) GLP(1) IRN(1) KEN(3) MDG(7) MUS(2) MWI(16) NLD(1) NZL(2) PRT(1) REU(1) RUS(1) SWE(1) USA (8) ZWE(77)	LAM11_ZWE
60	■■■■■■■■■■■■■■■■■■■■□□□□■■■■■■■■□□□□■■■□■■■	83	ARG(2) AUT(3) BEL(2) BRA(10) CUB(4) DEU(2) DZA(2) ESP(1) FXX(8) GBR(1) GNB(5) GUF(2) ITA(2) ITAS(1) MAR(3) MDG(1) MEX(3) MOZ(1) NLD(1) PRT(2) RUS(1) SEN(2) USA(11) VEN(11) ZAF(1) ZWE(1)	LAM4
61	■■■■■■■■■■■■■■■■■■■■■■□□□■■■■■■■□□□□■■■■■■■	331	AUT(1) BEL(10) BRB(1) CAF(7) CIV(8) CMR(156) CZE(14) DEU(5) ESP(2) FXX(40) GBR(2) GLP(1) GUF(1) ITA(4) LBY(4) MAR(2) MDG(9) MTQ(1) MYS(1) NLD(5) PRT(1) RUS(1) SAU(1) SEN(7) USA(53) VNM(1)	LAM10_CAM
62	■■■■■■■■■■■■■■■■■■■■■■■■■□□□□□□■□□□□■■■□■■■	171	ARG(2) AUS(2) AUT(11) BEL(5) CAF(13) CUB(1) CZE(4) DEU(1) DZA(3) ESP(1) FIN(4) FXX(18) GLP(2) GUF(2) HTI(1) HUN(3) ITA(8) LBY(1) MDG(8) MTQ(6) NLD(3) RUS(1) SWE(4) TUR(6) USA(54) VEN(4) ZAF(1) ZWE(2)	H1
63	□■■■■■■■■■■■■■■■■■■■■□■■■■■■■■■■□□□□■■■■■■■	5	ESP(1) GLP(2) GUF(1) MTQ(1)	T1
64	■■■■■■■■■■■■■■■■■■■■□□□□■■■■□■■■□□□□■■■■■■■	157	ARG(5) AUS(1) AUT(5) BEL(4) BRA(26) COM(1) CZE(2) DZA(1) FXX(1) GBR(1) GUF(13) IND(1) IRN(1) ITA(1) MDG(1) MEX(2) MTQ(1) MWI(2) MYS(2) PRT(14) SEN(1) USA(67) VEN(4)	LAM6

65	■■■■■■■■■■■■■■■■■■■■■■■■■■■■■■■■□□□□■□□■■■■	65	AUT(4) BRA(23) CMR(3) CZE(1) DEU(2) DZA(1) ESP(2) FXX(5) GLP(1) GUF(1) HTI(12) ITA(1) KAZ(1) MTQ(1) SWE(1) USA (6)	T1 (T4-CE1 ancestor?)
66	■■■■■■■■■■■■■■■■■■□□□□■■■■■■■■□■□□□□■■□■■■■	2	GUF(2)	H3-LAM
67	■■■■■■■■■■■■■■■■■■□□□□■■■■■■■■□■□□□□■■■■■■■	25	GLP(2) GUF(4) HTI(1) MEX(1) USA (17)	H3-LAM
68	■■■■■■■□■■■■■■■■■■■■■■□■■■■■■■■■□□□□■■■■■■■	50	ARG(26) AUT(4) BRB(1) CZE(5) DEU(3) FXX(1) GBR(1) IND(1) ITA(2) MTQ(1) NLD(2) SWE(2) USA (1)	T5
69	suppressed	2	SUPPRESSED SHARED-TYPE	
70	■■■□□□□□□□□□■■■■■□■■■■■■■■■■■■■■□□□□■■□■■■■	72	GBR(2) GLP(2) GUF(6) HTI(8) MEX(3) PHL(2) USA(49)	X3
71	■■■■■■■■□□■■□■■■■■■■■■■■■■■■■■■■□□□□■■■■■■■	33	ARG(1) CAN(2) CUB(5) FXX(4) IND(1) ITA(9) ITAS(3) NLD(1) USA(2) VEN(2) ZAF(3)	S
72	■■■■■■■■■■□■■■■■■■■■■■■■■■■□□□□□□□■■■■■□■■■	16	GLP(1) GUF(7) USA (8)	EAI undefined
73	■■■■■■■■■■■■□■■■■■■■■■■■■■■■■■■■□□□□■■■□■■■	114	AUS(1) AUT(5) BEL(3) BRA(4) EGY(7) FIN(1) FXX(9) GEO(3) GLP(1) GNB(1) GUF(6) HTI(1) IDN(4) ITA(22) MDG(3) MEX(1) MOZ(2) MTQ(1) NLD(2) PRT(3) SWE(1) USA(27) VEN(1) VNM(4) ZWE(1)	T2-T3
74	■■■■■■■■■■■■■■■■■■■■□□□□□□□□□■■■□□□□■■■■■■■	6	CUB(3) MDG(3)	T1
75	■■■■■■■■■■■■■■□■■■■■■■■■■■■■■■□■□□□□■■■■■■■	29	ARG(3) BRA(1) CMR(5) DEU(1) FXX(7) GLP(2) HTI(1) HUN(1) MDG(1) MEX(1) PRT(1) ROM(2) USA (3)	H3
76	■□□■■■■□□□□□□□□□□■□■■■■■■■■□□□□■□■■■■■■■■■■	2	GUF(2)	EAI5 or EAI3
77	■■■■□□□■■■■■■■■■■■■■■■■■■■■■■■■■□□□□■■■■■■■	11	GLP(1) GUF(4) HTI(4) USA (2)	T1
78	■■■■■■■■■■■■■■■■■■■■■■■■■■■■■■■■□□□□■■■□□■■	32	AUT(2) CZE(1) ESP(1) FIN(1) GBR(3) GUF(2) IND(3) ITA(1) MDG(13) NLD(1) SAU(2) USA (2)	T1-T2
79	■□■□□■■■■□■■■□□■■■■■□□□□■■□□□□■■□□□□■■□■■□■	2	ZWE(2)	LAM11_ZWE
80	■■■■■□■■■■■■■■■■■■■■■■□□■□□□□□□■□□□□■■■■■■■	8	CUB(8)	H1
81	■■■■■■■■■□■■■■■■■■■■□□□□■■■■■■■■□□□□■■■■■■■	20	ARG(1) BRA(1) CUB(16) ITA(1) ZWE(1)	LAM9
82	■■■■■■■■■■■■■□■■■■■■□□□□■■□□□□■■□□□□■■■□■■■	4	ZWE(4)	LAM11_ZWE
83	■■■■■■■■■■■■■□■■■■■■■■■■■■■■■■■■□□□□■■□■■■■	2	ZWE(2)	T1
84	■■■■■■■■■■■■■■■□■■■■□□□□■□□□□□■■□□□□■■■□■■■	2	ZWE(2)	LAM11_ZWE
85	■□■■□■■■■■■■■□■■■■■■□□□□■■□□□□■■□□□□■■■■■■■	2	ZWE(2)	LAM11_ZWE
86	■■■■■■■■■■■■■■■■■■■■■□■■■■■■■■■■□□□□■■■■■■■	46	AUT(2) ESP(2) FXX(4) GUF(1) ITA(1) ITAS(1) MDG(27) NLD(1) TUR(2) USA(4) VEN(1)	T1
87	■□■■■■■■■■■■■□■■■■■■□□□□■■□□□□■■□□□□■■■■■■■	2	ZWE(2)	LAM11_ZWE
88	■■■■■■■■□□■■□■■■■■■■■■■■■■■■■■■■□□□□■■■□■■■	3	CUB(1) DZA(1) GUF(1)	S
89	■■□■■■■□□□□□□□□□□□□□□□□□□■■■□□□□■□■■■■■■■■■	67	AUS(1) FXX(1) GLP(1) GUF(1) IDN(2) MYS(4) NLD(5) SWE(3) THA(36) USA(11) VNM(2)	EAI2_NTB
90	■■□■■■■■■■■■■■■■■■■■■■■■■■□□□□□□□□□□□■■■■■■	6	AUT(2) ITA(1) NLD(1) POL(2)	U
91	■■■□□□□□□□□□■■■■■□■■■■■■■■■■■■■■□□□□■■■■■■■	143	ARG(3) BRA(1) CAN(8) DNK(5) ESP(2) GBR(6) GLP(1) GUF(5) HTI(29) NLD(2) PER(1) USA (79) VEN(1)	X3
92	■■■□□□□□□□□□■■■■■□■■■■■■■■■■■■■■□□□□■■■■■■■	123	ARG(1) AUS(2) AUT(1) BRA(3) CUB(2) DNK(4) GBR(14) GNB(1) GUF(6) MEX(2) MWI(1) NLD(8) NOR(1) NZL(2) USA(60) ZAF(14) ZWE(1)	X3
93	■■■■■■■■■■■■□■■■■■■■□□□□■■■■■■■■□□□□■■■■■■■	188	ARG(2) AUS(1) BRA(13) DEU(3) ESP(2) FXX(1) GLP(7) GUF(2) HTI(15) ITA(14) ITAS(1) NLD(1) USA(44) VEN(82)	LAM5
94	■■■■■■■■■■■■■■■■■■□■■■□□■■■■□■■■□□□□■□□■■■■	5	GUF(5)	T4-CEU1
95	■■■■■■■■■■■■■■■■■■■■□□□□■■■■□■■■□□□□■■■□■■■	11	BRA(2) GBR(1) GUF(2) USA(6)	LAM6
96	■■■■■■■■■■■■■■■■■■■■■■□■□□□□□□□□□□■■□■■■■■■	42	BGD(36) GUF(4) USA (2)	EAI7_BGD2
97	■■■■■■■■■■■■■■■■■■■■■■■□□□□■■■■■□□□□■■■■■■■	3	MNG(2) SWE(1)	T1
98	□□■■■■□□□□□□□□□□■■■■□□□□■■■■■■■■□□□□■■■■■■■	4	DEU(1) MNG(3)	LAM9?
99	■■■■□■■■■■■■■■■■■■■■■■■■■■■■■■□■□□□□■■■■■■■	31	ARG(1) AUT(3) BRA(1) CZE(2) DEU(1) DZA(1) FIN(1) IND(1) ITA(2) JPN(1) MDG(6) NLD(3) ROM(1) USA (7)	H3
100	■■■■■■■■■■■■■■■■■■■■■■■■■■■■■■■■■□■■■■■■■■■	39	FXX(1) GBR(6) IND(23) MYS(1) THA(1) USA(6) VNM(1)	MANU1
101	■■■■■■■□□□□□■■■■■■■■□□□□■■□■■■■■□■■■□□□■■■■	16	CMR(8) FXX(4) GLP(2) USA (2)	AFRI_2
102	■■■■■■■■■■■■□□□□■■■■■■■■■■■■■■■■□□□□■■■■■■■	46	ARG(3) AUS(3) BRA(5) DEU(2) FXX(3) GLP(2) GUF(1) IDN(5) ITA(1) MAR(1)	T1

			MEX(2) MUS(1) MYS(3) RUS(2) USA (11) ZAF(1)	
103	■■■■■■■■■■■■■■■■■■■■■■□■■□□□□□□■□□□□■■■□■■■	3	GLP(3)	H1
104	■□■■■■■■□□□■■■□■■■■□□□□□□□□□□□□□□□□□□□■■■■■	2	ESP(2)	U (LAM3?)
105	■■■■■■■■□□□■■■□□□□□□□□□□□□□□□□□□□□□□□□□□■■■■	24	BRA(1) DZA(1) ESP(5) FXX(2) NLD(1) USA (14)	U (LAM3?)
106	■■■■■■■■□□□■■■■■■■■□□□□□□□□□□□□□□□□□□□■■■■■	43	ARG(5) AUS(1) BEL(1) BRA(3) DZA(3) ESP(6) FXX(3) REU(1) USA(18) VEN(2)	U (LAM3?)
107	□□□□■■■■□□■■■■■■■■■■■■■■■■■■■■■■□□□□■■■■■■■	4	AUS(1) FXX(3)	S
108	□■■■■■■■■■■■■■■■■■■■■■■■■■■■■■□■□□□□■■■□■■■	2	FXX(2)	H3
109	■□□■■■■■■■■■■■■■■■□■■■■■■■■■□□□□■□■■■■■■■■■	51	DNK(1) FXX(2) MDG(46) NLD(1) USA (1)	EAI8_MDG
110	■■■■■■□□□■■■■■■■■■■■■■■■■□■■■■□■□□□□■■■■■■■	2	FXX(2)	H3
111	■■■■■■■■□□□■■■□■■■■■□□□□■■■■■■■■□□□□■■■■■■■	15	ARG(6) BRA(2) FXX(4) PRT(1) USA (2)	LAM3
112	■■■■■■■■■■■■□■■■■■■■■■■□□□■■■■■■□□□□■■■■■■■	5	FXX(5)	T3
113	■□□■■■■■■■■■■■■■■■■■■■■■■■□■□□□□■□■■□□□■■■■	4	FXX(3) IND(1)	EAI3_IND
114	■■■■■■■■■■■■■■□■■■■■■■■□□□□■■■■■■□□□□■■■■■■■	2	FXX(2)	T1
115	■■■■■■■■■■■■■■□■■■■■■■■□□□■■■■■■■□□□□■■■■■■■	21	BRB(1) CMR(11) FXX(8) ITA(1)	LAM10_CAM
116	■■■■■■■■■■■■■■□■■■■■■■■■■□■■■■□■□□□□■■■■■■■	2	FXX(2)	H3
117	■■■■■■■■■■■■■■□■■■■■■■■■■■■■■■■■□□□□■■■□■■■	14	ARG(1) CAF(2) CMR(3) DNK(1) FXX(6) ROM(1)	T1
118	■■■■■■■■■■■■■■□■■■■■■■■■■■■■■■■■□□□□■■■■■■■	76	ARG(5) AUT(1) AZE(1) BRA(1) CAF(2) CMR(1) CZE(4) DEU(1) DNK(2) EGY(1) FIN(1) FXX(21) GUF(1) IDN(1) IND(2) ITA(4) ITAS(1) KAZ(1) MDG(1) MTQ(1) POL(1) PRT(2) SWE(1) TUR(2) USA (8) VEN(9)	T2
119	■■■■■■■■■■■■■■■■■□■■■■■■■■■■■■■■□□□□■■■■■■■	659	?(1) AUT(2) BEL(1) BRA(10) CAN(7) DNK(1) ESP(2) FIN(1) FXX(4) GBR(25) GNB(1) HTI(1) IND(5) ITA(2) ITAS(1) LBY(1) LVA(3) MEX(46) MTQ(3) MWI(1) NLD(15) NOR(2) RUS(1) SWE(1) USA(90) USA (291) USA (111) ZAF(29) ZWE(1)	X1
120	■■■■■■■■■■■■■■■■■■■□■■■■■■■■■■■■□□□□■■■■■■■	5	AUT(2) FXX(3)	T1
121	■■■■■■■■■■■■■■■■■■■■■■■■■□■■■■□■□□□□■■■■■■■	14	BRA(1) CZE(2) ESP(1) FIN(1) FXX(4) GBR(1) ITA(1) SWE(1) USA(2)	H3
122	■■■■■■■■■■■■■■■■■■■■■■■■■□■■■■■■□□□□■■■■■■■	13	AUT(1) FXX(6) IND(1) ITAS(1) KEN(1) MYS(1) USA(1) VNM(1)	T1
123	■■■■■■■■■■■■■■■■■■■■■■■■■■□□■■■■□□□□■■■■■■■	14	AUT(8) BGD(3) FXX(1) GLP(1) NLD(1)	T1
124	■■■■■■■■■■■■■■■■■■■■■■■■■■■■■■□□□□□□■■■■■■■	22	AUT(1) BRA(1) DEU(2) FIN(1) FXX(3) LKA(2) NLD(2) RUS(1) USA(9)	U
125	□□□□□□□□□□□□□□□□□□□□□□□□■■■■■■■■□□□□■■■□■■■	13	BDI(1) BEL(1) CAN(1) DEU(2) EAFR(1) FXX(1) IRN(2) ITA(1) NLD(1) RWA(1) SAU(1)	T2
126	■□□■■■■■■■■■■■■■■■■■■■■■■■■■□□□□■□■■■■■■■■■	35	BEL(1) BGD(4) DNK(3) GBR(15) IND(4) MDG(1) MYS(1) NLD(4) SEN(1) SWE(1)	EAI5
127	■□■■■■■■■■■■■■■■■■■■■■■■■■■■□□□■□□□□■■■■■■■	48	AUS(1) AUT(9) DEU(7) FXX(1) GBR(1) IRN(19) ITA(1) NLD(5) RUS(1) SWE(1) USA (2)	H4
128	■■□□■■■■■■■■■■■■□■■■■■■■■■■■■■■■□□□□■■■□■■□	9	BEL(2) DEU(1) EAFR(4) RWA(1) UGA(1)	T2
129	■■■□□□□□□■■■■■■■■■■■■■□□■■■■□□□□■□■■■■■■■■■	11	DEU(1) GUF(2) MDG(1) MOZ(1) MWI(4) ZWE(2)	EAI5
130	■■■■■■■■□□□■■■■■■■■■□□□□■■■■■■■■□□□□■■■□■■■	34	AUT(1) BRA(1) CHL(1) CUB(1) ESP(1) ITA(4) MDG(1) USA (21) ZAF(3)	LAM3
131	■■■■■■■■■■■■□□■■■■■■■■■■■■■■■■■■□□□□■■■■■■■	28	ARG(1) ARM(1) AUT(1) FIN(2) FXX(1) GLP(1) GUF(9) ITA(1) MTQ(1) POL(1) TUN(1) USA (8)	T1
132	■■■■■■■■■■■■■■■■■■■■□□□□■■□□□□□□□□□□□□□□■■■	7	CUB(1) ECU(1) MEX(1) USA(3) VEN(1)	U
133	■■■■■■■■■■■□□□□□■■■□□□□□■□■■■■■■□□□□■□□□■■□	2	GLP(1) NLD(1)	T1
134	■■■■■■■■■■■■■■■■■■■■■■■■■■■■■■□■□□□□■■□□■■■	10	FXX(2) GUF(1) ITA(5) MTQ(1) SWE(1)	H3
135	■■■■■■■■■■■■■■■■■■■■■■■■■■■■■■■■□□□□■■■□■■□	19	AUT(3) DEU(2) EAFR(4) FXX(2) GBR(1) KEN(1) MYS(1) UGA(2) USA (3)	T2
136	■■■■■■■■■■■□□□□□■■■□□□□□■□■■■■■■□□□□■□□■■■■	30	BEL(1) CAF(2) DZA(10) ESP(1) FXX(6) GBR(2) GUF(2) IDN(2) NLD(3) USA(1)	T1
137	■■■■■■■■■■■■■■■■■□■■■■■■■■■■■■■■□□□□■■□□□□■	720	AUS(5) BRA(7) BRB(1) CAN(11) CMR(7) DNK(15) ESP(5) FXX(1) GBR(55) GUF(2) HTI(1) ITA(3) LBY(1) MEX(1) MYS(1) NLD(9) PRT(1) SAU(1) SWE(2) USA(584) ZAF(7)	X2
138	■■■■■■■■■■■■■■■■■■■■■■■■■■■■□□□□■□■■■■■□□□□	69	BGD(26) DNK(9) FIN(1) GBR(8) GEO(1) GLP(1) IND(2) MYS(1) NLD(7) SUR(1) SWE(1) USA(11)	EAI5
139	■■■■■■■■■■■■■■■■■■■■■■■■■□□■□□□□■□■■■■■■■■■	306	AUS(3) DNK(32) FIN(1) GBR(1) IND(1) NLD(4) NZL(2) SWE(5) USA(76) VNM(181)	EAI4_VNM
140	■■■□□□□■□■■■■■■■■■■■■■□□□□□□□□□□□□■■□□■■■■■	2	FXX(2)	CAS1_DELHI
141	■■■□□□□■■■■■■■□■■■■■■■□□□□□□□□□□□□■■■■■■■■■	10	FXX(3) IND(4) NLD(1) USA (2)	CAS1_DELHI
142	■■■□□□□■■■■■■■■■■■■■■□□□□□□□□□□□□□■■■■■■■■■	13	BGD(1) DEU(1) FXX(2) GBR(1) IND(1) NLD(1) SAU(1) SDN(1) SWE(1) USA(3)	CAS

143	■■■■□■■■■■□□□□□□□□□□□□□□■□□□□□□■□□□□■■■■■■■	5	DZA(1) FXX(2) NLD(2)	H1
144	■■■■■■□□□□□□□□□□□□□□□□□□□■■■■■■■□□□□■■■■■■■	10	DEU(1) FXX(4) GBR(1) NLD(1) PRT(1) USA (2)	T1
145	■■■■■■□□□□□□□□□■■■□□□□□□□□□□□□□□□□■■□□□■■■■	3	FXX(3)	AFRI
146	■■■■■■■□□□□□□□□□□□□□■■■■■■■■■■■■■■■□□□□■■■	2	FXX(2)	AFRI
147	■■■■■■■□□■■■■■■■■■■■□□□■■■□□□□□□□□□□□■■■■■■	3	FXX(3)	U
148	■■■■■■■□□■■■■■■■■■■■■□□■■■□□□□□□□□□□□■■■■■■	2	FXX(2)	U
149	■■■■■■■■■□□□□□□□□□□■■■■■■■■■■■■■□□□□■■■■■■■	208	AUT(1) BEL(1) DEU(1) DNK(57) ETH(79) FIN(1) FXX(9) GBR(13) ITA(2) LBY(1) NLD(16) NOR(2) SWE(6) USA(19)	T3_ETH
150	■■■■■■■■■■■■■■□■■■■■□□□□■■■■■■■■□□□□■■■■■■■	65	ARG(6) BEL(22) BRA(3) DZA(1) EGY(1) FIN(1) FXX(8) GUF(2) ITA(3) ITAS(1) MAR(1) MEX(1) PRT(9) RUS(1) USA (1) VEN(4)	LAM9
151	■■■■■■■■■■■■■■□■■■■■■■■■■■□□□□□□■□□□□■■■■■■■	15	ARG(1) AUT(1) CAF(2) CUB(2) EGY(1) FXX(3) HTI(1) ITA(1) POL(1) PRT(1) SWE(1)	H1
152	■■■■■■■■■■■■■■■■■■■■■■■■■■□■□□□□■□■■■■■■■■■	26	FXX(1) GUF(1) ITA(1) NLD(2) SWE(1) USA(12) VNM(8)	EAI5
153	■■■■□■■■■■■■■■■■■■■■■■■■■■■■■■■■□□□□■■■□■■■	44	AUT(1) BRA(4) CUB(5) ETH(1) FIN(4) ITA(20) NLD(1) SWE(2) USA (6)	T2
154	■■■■□■■■■■■■■■■■■■■■■■■■■■■■■■■■□□□□■■■■■■■	41	ARG(3) AUS(1) AUT(12) DEU(3) ESP(1) FIN(1) FXX(2) HUN(1) ITA(9) NLD(1) RUS(1) USA (6)	T1
155	■■■■■■■■□□□■■□■■■■■■■■■■■■■■■■■■□□□□■■■■■■■	2	ITA(2)	T1
156	■■■■■■■■□□□■■■■■■■■■■■■■■■■■■■■■□□□□■■■■■■■	33	AUT(1) BRA(2) ESP(1) ITA(4) MDG(22) USA(3)	T1
157	■■■■■■■■■■■■□■■■■■■■■■■■■■■■■■■■□□□□■□□■■■■	7	DEU(1) ITA(2) PRT(4)	T3
158	■■■■■■■■■■■■□■■■■■■■■■■■■■■■■■■■□□□□■■□□□□■	3	ITA(2) ZAF(1)	T3
159	■■■■■■■■■■■■■□□□□□□□□□□■■■■■■■■■□□□□■■■■■■■	54	ARG(10) AUS(1) AUT(8) DEU(1) EST(2) FXX(1) ITA(23) ITAS(3) LVA(1) MEX(1) USA (3)	T1 (Tuscany variant)
160	■■■■■■■■■■■■■■■■■■■□□■■■■■■■□□□□□□□□□□□□□□■	13	ARG(1) BEL(2) CUB(1) ESP(2) ITA(2) ITAS(1) USA (4)	U
161	■■■■■■■■■■■■■■■■■■■■□□□□■■■■■■■□□□□□■■■■■■■	4	ITA(1) NLD(1) RUS(1) USA(1)	U
162	■■■■■■■■■■■■■■■■■■■■□□□□■■■■■■■■□□□□■■□■■■■	13	ARG(1) FIN(1) FXX(2) ITA(1) ITAS(2) USA(1) VEN(5)	LAM9
163	■■■■■■■■■■■■■■■■■■■■□□□□■■■■■■■■□□□□■■■□□□□	21	HTI(3) ITA(4) MEX(2) USA (12)	LAM9
164	■■■■■■■■■■■■■■■■■■■■■■■□□□□□□□■■□□□□■■■■■■■	24	AUT(2) FXX(1) HUN(1) ITA(14) ITAS(1) MDG(1) NLD(1) USA(3)	T1 (H?)
165	■■■■■■■■■■■■■■■■■■■■■■■■■□□■■■□■□□□□■■■□■■■	3	ITA(3)	H3
166	■■■■■■■■■■■■■■■■■■■■■■■■■■■□■■□■□□□□■□■■■■■	6	ITA(5) USA (1)	H3
167	■■■■■■■■■■■■■■■■■■■■■■■■■■■■■□■■□□□□■■■■■■■	30	AUT(4) BEL(1) BRA(1) CUB(1) ESP(4) FIN(2) FXX(1) GBR(1) IDN(1) ITA(1) MNG(1) NLD(3) SDN(1) TUR(1) USA (5) VEN(2)	T1
168	■■■■■■■■■■■■■■■■■■■■■■■■■■■■■■□■□□□□■■□■■■■	16	AUT(1) GEO(1) GLP(1) HTI(4) ITA(1) MYS(1) NLD(4) USA(3)	H3
169	■■■■■■■■■■■■■■■■■■■■■■■■■■■■■■■□□□□□■■■□□□□	6	ITA(5) ITAS(1)	U
170	■■■■■■■■□□□□□□■■■■■■■■■■■■■■■■■■□□□□■□□□□□□	2	FXX(2)	T1
171	■■■■■■■■□□□□□□■■■■■■■■■■■■■■■■■■□□□□■■■■■■■	2	FXX(2)	T1
172	■■■■■■■■■■■■■■■■■■■■■■■■■■■■■■■□□□□□■■■■■■■	32	AUT(7) BEL(1) DEU(2) FIN(1) FXX(4) HTI(2) ITA(5) MYS(1) NLD(1) PAK(1) RUS(1) USA (6)	U
173	■■■■■■■■■■■■■■□■■■■■□■■■■■■■■■■■□□□□■■■■■■■	6	FXX(4) TUR(1) USA (1)	T1
174	■■■■■■■■■■■■■■■■■■□□□□■■■■■■■■■■□□□□■■■■■■■	11	FXX(5) HTI(1) RUS(1) USA (3) VNM(1)	T1
175	■■■■■■■■■■■■■■■■■■■■□■■■■■■■■■■■□□□□■■■□■■■	18	ARG(8) FXX(1) HTI(2) ITA(4) PER(1) SUR(1) USA (1)	T2
176	■■■■■■■■■■■■□■■■■■■■□□□□■■■■□■■■□□□□■■■■■■■	16	BRA(1) CAF(1) GUF(2) USA (12)	LAM5
177	□■■■■■■■■■■■■■■■■■■■□□□□■■■■■■■■□□□□■■■■■■■	21	ARG(1) BRA(8) EGY(2) ESP(1) FXX(2) GUF(1) ITA(3) MYS(1) USA(2)	LAM9
178	■□□■■■■■■■■■□■■■■■■■□□□□■■■■■■■■□□□□■■■□■■■	4	CUB(1) SEN(1) VEN(2)	LAM4
179	■■□■■■■■■■■■□■■■■■■■□□□□■■■■■■■■□□□□■■■□□■■	8	BRA(6) GUF(1) PRT(1)	LAM2
180	■■□■■■■■■■■■■■■■■■■■■■■■■■■■■■□■□□□□■■■■■■■	23	AUT(3) CAF(6) CIV(1) CZE(2) ESP(4) MYS(1) NLD(1) POL(1) RUS(1) SWE(1) USA (1) VNM(1)	H3
181	■■■■■■□□□■■■■■■■■■■■■■■■■■■■■■■■■■■■■□■■■■	126	?(1) BEL(1) DEU(3) DNK(1) FXX(6) GBR(3) GNB(72) GUF(1) ITA(7) NLD(9) SEN(7) SWE(1) USA (14)	AFRI_1
182	■■■■■■■■■■■■■■■■■■□□□□□□■□□□□□□■□□□□■■■■■■■	2	NLD(1) SEN(1)	H1
183	■■■■■■■■■■■■■■■■■■□■■■■■■■■■■■□■□□□□■■■■■■■	19	CZE(2) DEU(1) ESP(4) FXX(1) HUN(1) ITA(4) SEN(2) USA (4)	H3
184	■■■■■■■■■■□□□□■■■■■■□□□□■■□□□□■■□□□□■■■□■■■	11	FXX(2) ZWE(9)	LAM11_ZWE

185	■■■■■■■■■■■■■■□■■□■■■■■■■■■■■■■■□□□□■■□□□□■	11	BRA(1) DNK(3) FXX(2) GLP(1) USA (4)	X2
186	■■■■■■■■■■■■■■□■■■■□□□□□■□□■■■■■□□□□■■■■■■■	3	EGY(1) FXX(2)	LAM7_TUR
187	■■■■■■□□□■■■■■■■■■■■■■■■■■■■■■■■□■■■■■□■■■■	39	FXX(1) GBR(1) GIN(1) GNB(27) ITA(1) MDG(1) SEN(1) USA (6)	AFRI_1
188	■■■■■■□□□■■■■■■■■■■■■■■■■■■■■■■■■■■■■□□■■■	3	GBR(1) GNB(2)	AFRI
189	■■■■■■■■■■■■■□□□□■■■■■■■■■■■■■■■□□□□■■■■■■■	8	CUB(2) FIN(1) PER(1) SWE(1) USA (3)	T1
190	□□□□□□□□□□□□□□□□□□□□□□□□□□□□□□□□□□■■■■■□■■■	48	CUB(1) GEO(2) IDN(1) LVA(1) MYS(2) NLD(1) RUS(3) THA(5) USA (30) VNM(2)	BEIJING
191	□□■■■■■■■■■■■■■■■■■■■■■■■■■■■■■■□□□□■■■■■■■	10	ARG(1) AUT(1) GEO(1) GLP(1) NLD(1) RUS(2) USA (3)	T1
192	■□□□□□□□□□□□□□□□■■■■■■■■■■■■□□□□■□■■■■■■■■■	2	USA (2)	EAI5
193	■■□■■■■■■■■■□■■■■■■■□□□□■■■■■□■■□□□□■■■■■■■	17	GUF(1) HTI(8) USA (8)	LAM2
194	■■□■■■■■■■■■□■■■■■■■□□□□■■■■■■■■□□□□■■■□■■■	12	BRA(1) MEX(2) USA (4) VEN(5)	LAM2-LAM4
195	■■□■■■■■■■■■■■■■■■■■□□□□■■■■■□■■□□□□■■■■■■■	4	BRA(2) GLP(1) USA (1)	LAM1
196	■■□■■■■■■■■■■■■■■■■■■■■■■■■■■■■■□□□□■■■■■■■	35	AUT(4) BRA(3) DZA(3) FXX(1) GEO(1) HTI(1) ITA(3) MDG(4) NLD(3) RUS(1) SWE(2) TUR(3) USA(3) VEN(2) ZAF(1)	T1
197	■■■□□□□□□□□□□■■■■□■■■■■■■■■■■■■■□□□□■■■□■■■	244	GBR(1) IND(3) USA(7) USA (229) USA (4)	X3
198	■■■□□□□□□□□□■□□□□□■■■■■■■■■■■■■■□□□□■■■■■■■	28	USA (28)	T1
199	■■■□□□□□□□□□■■■■■□■■■■■□□□□□□□□□□□□□□□□□■■	15	MEX(2) USA(13)	U
200	■■■□□□□□□□□□■■■■■□■■■■■■■■■■■■■■□□□□■■■□□□□	27	AUS(1) CIV(1) GBR(5) IND(1) MEX(1) MWI(2) NOR(1) USA(15)	X3
201	■■■□□□□□□□■■■■■■■■□□□□□□□□□□□□□□□□■■■■■■■■■	2	USA (2)	CAS
202	■■■□□□□□■■■■■■■■■□■■■■■■■■■■■■■■□□□□■■□□□□■	3	USA (3)	X2
203	■■■□□□□■■■■■■■■■■■■■■■□□□□□□□□□□□□□□■■■■■■■	8	FXX(1) SAU(1) USA (6)	CAS
204	■■■□■■■■■■■■■■■■■■■■■■■■■■■■□□□□■□■■■■■■■■■	10	FXX(1) THA(1) USA (7) VNM(1)	EAI5
205	■■■□■■■■■■■■■■■■■■■■■■■■■■■■■■■■□□□□■■■■■■■	15	AUS(1) BRA(2) DEU(1) FXX(1) GBR(1) HTI(1) KEN(1) MYS(1) USA (4) VEN(1) VNM(1)	T1
206	■■■■□□□□□■■■■■■■■■■■□□□□■■■■■■■■□□□□■■■■■■■	7	BEL(1) USA(6)	LAM9
207	■■■■■□■■■■■■■■■■■■■■■■■■■■■■■■□■□□□□■■■■■■■	12	AUT(2) MTQ(1) USA(9)	H3
208	■■■■■■■□□□□□■■■■■■■■■■■■■□□■□□□□■□■■■■■■■■■	2	USA (2)	EAI4_VNM
209	■■■■■■■■□□□□□□■■■■■■□□□□■■■■■■■■□□□□■■■■■■■	23	BEL(2) CUB(3) ESP(10) FXX(5) ITA(1) USA(2)	LAM12_MAD1
210	■■■■■■■■□□□□□■■■■■■■■■■■■■■■■■■■□□□□■■■■■■■	104	USA(104)	T1
211	■■■■■■■■□□□■□■■■■■■■□□□□■■■■■■■■□□□□■■■■■■■	50	ARG(1) AUS(1) ESP(1) FXX(2) MDG(1) MEX(12) PRT(3) USA(28) ZAF(1)	LAM3
212	■■■■■■■■□□■■■■■■■■■■■■■■■■□■■■■■□□□□■■■■■■■	11	AUT(1) CAN(7) USA(3)	S
213	■■■■■■■■□■■■■■■■■■■■□□□□■■■■■■■■□□□□■■■□■■■	9	ITAS(2) MYS(1) USA(6)	LAM4
214	■■■■■■■■■■■■□□□□■■■■■■□■■■■■■■■■■□□□□■■■■■■■	45	USA(45)	T5
215	■■■■■■■■■■■■□□□□■■■■■■■■■■■□■■■■□□□□■■■■■■■	5	ESP(3) USA (2)	T1
216	■■■■■■■■■■■■□□■■■■■■□□□□■■■■■■■■□□□□■■■■■■■	9	ARG(1) ITA(2) USA (5) VEN(1)	LAM9
217	■■■■■■■■■■■■□■■■■□■■■■■■■■■■■■■■□□□□■■■■■■■	15	AUS(1) GBR(1) MEX(2) USA(11)	X1
218	■■■■■■■■■■■■□■■■■■■■■■■■■□□□□□□■□□□□■■■■■■■	14	AUT(3) BEL(1) ESP(1) IDN(1) NLD(1) RUS(1) USA (5) ZWE(1)	H1
219	■■■■■■■■■■■■■□□□□□■■■■■■■■■■■■■■□□□□■■■■■■■	30	PER(2) USA(28)	T1
220	■■■■■■■■■■■■■□□□■□■■■■■■■■■■■■■■□□□□■■□□□□■	7	USA (7)	X2
221	■■■■■■■■■■■■■■□■■□■■■■■■■■■■■■■■□□□□■■■■■■■	7	LBY(1) USA(6)	X1
222	■■■■■■■■■■■■■■■■□□□□□■■■■■■■□■■■□□□□■■■■■■■	11	ESP(3) MEX(1) PER(1) USA (6)	T1
223	■■■■■■■■■■■■■■■■■□□■■■■■■■■■■■■■□□□□■■□□□□■	5	USA(5)	T1
224	■■■■■■■■■■■■■■■■■□■■■■■■■■□□■■■■□□□□■■■□□■■	8	USA(8)	X1
225	■■■■■■■■■■■■■■■■■□■■■■■■■■■■■■■■□□□□■■□□□■■	4	FXX(1) USA (3)	X1
226	■■■■■■■■■■■■■■■■■□■■■■■■■■■■■■■■□□■■■■■■■■■	3	BEL(1) USA (2)	MANU2
227	■■■■■■■■■■■■■■■■■■□■■■■■□□□□□□□■□□□□■■■■■■■	5	USA (5)	H3
228	■■■■■■■■■■■■■■■■■■■■□□□□□■■■□■■■□□□□■■■■■■■	5	USA (5)	T1

229	■■■■■■■■■■■■■■■■■■■■□□□□□■■■■■■■□□□□□■■■■■■	2	USA (2)	U
230	■■■■■■■■■■■■■■■■■■■■□□□□□■■■■■■■□□□□■■■■■■■	15	GNB(1) HTI(1) USA (13)	T1
231	■■■■■■■■■■■■■■■■■■■■■■□■■■■■■■■■■□□□□■■■□□□□	2	USA (2)	T5
232	■■■■■■■■■■■■■■■■■■■■■■■□□□□□□□□□□□□□□□■■■■■	6	MEX(1) NLD(1) USA (4)	U
233	■■■■■■■■■■■■■■■■■■■■■■■■□□□□□□■■□□□□■■■□■■■	9	USA(9)	T2
234	■■■■■■■■■■■■■■■■■■■■■■■■■■■■□□□□■□■■□■■■■■■	17	AUS(1) BGD(3) DNK(1) NLD(1) USA (7) VNM(4)	EAI5
235	■■■■■■■■■■■■■■■■■■■■■■■■■■■■□□□□■□■■■□■□■■■	2	USA (2)	EAI1_SOM
236	■■■■■■■■■■■■■■■■■■■■■■■■■■■■□□□□■□■■■■■■■■■	91	AUS(5) BEL(1) BGD(6) DNK(4) FXX(1) GBR(3) GEO(2) GNB(1) IDN(1) MYS(7) NLD(3) NOR(1) NZL(2) PAK(1) SEN(1) SWE(2) THA(12) USA(32)VNM(5) ZWE(1)	EAI5
237	■■■■■■■■■■■■■■■■■■■■■■■■■■■■■■□□□□□□□□□□□□□	74	AUT(10) BEL(2) CMR(5) DEU(8) FIN(1) FXX(2) GBR(1) IDN(5) ITA(5) ITAS(1) MDG(3) POL(6) PRT(1) RUS(7) SWE(2) USA (13) ZAF(2)	U (likely H3)
238	■■■■■■■■■■■■■■■■■■■■■■■■■■■■■■□□□□□□□□■■■■■	7	FXX(2) USA (5)	U
239	■■■■■■■■■■■■■■■■■■■■■■■■■■■■■■■■□□□□□□□□■■■	44	BGD(2) BRA(1) MEX(7) USA(31) VEN(3)	U
240	■■■■■■■■■■■■■■■■■■■■■■■■■■■■■■■■□□□□□■■■■■■	14	AUT(1) BRA(5) CUB(1) DEU(2) GUF(1) ITA(1) USA(3)	U
241	■■■■■■■■■■■■■■■■■■■■■■■■■■■■■■■■□□□□■□□□□■■	11	FIN(2) USA(9)	T1
242	■■■■■■■■■■■■■■■■■■■■■■■■■■■■■■■■□□□□■□□□■■■	5	ARG(1) GUF(1) HTI(2) USA (1)	T1
243	■■■■■■■■■■■■■■■■■■■■■■■■■■■■■■■■□□□□■■□□□□□	16	BEL(3) DNK(1) ITA(1) MYS(5) USA (6)	T1
244	■■■■■■■■■■■■■■■■■■■■■■■■■■■■■■■■□□□□■■□□□□■	44	ARG(1) BEL(1) BGD(9) BRA(3) FXX(4) GNB(2) HTI(2) IND(1) MDG(1) NLD(1) PRT(16) USA (3)	T1
245	■■■■■■■■■■■■■■■■■■■■■■■■■■■■■■■■□□□□■■□■■■■	11	ARG(1) AUT(1) BRA(1) ESP(1) GNB(1) GUF(1) NLD(1) USA (2) VEN(1) ZWE(1)	T1
246	■■■■■■■■■■■■■■■■■■■■■■■■■■■■■■■■■■■■■■■□■■■	13	MYS(2) NLD(2) THA(1) USA (8)	U
247	■■■□□□□■■■■■■■■■■■■■■■□□□□□□□□□□□□■■■□□■■■■	4	FXX(1) SAU(1) USA (2)	CAS1_DELHI
248	■■■■■□□■■■■■■■■■■■■■■■■■■□□■□□□□■□■■■■■■■■■	4	USA (2) VNM(2)	EAI4_VNM
249	■■■■■■■■■■■□■□□□□□□□□□□■■■■■■■■□□□□■■■■■■■	15	AUS(1) MYS(1) NLD(1) NZL(2) USA (7) VNM(3)	T1
250	□□□□□□□□□□□□□□□□□□□□□□□□□□□□□□□□□□□□□■■■■■■	12	AUS(1) FXX(1) IDN(2) MYS(1) USA(5) VNM(2)	BEIJING-LIKE
251	■■■■■■■□□□□□□□□□□□□□□□□□□□□■■■■■□□□□■■■■■■■	11	FIN(1) MAR(2) NLD(1) RUS(6) USA (1)	T1
252	■■■■■■■■■■□□■■■■■■■■□□□□■■■■■■■■□□□□■■■■■■■	39	LVA(3) RUS(32) USA (4)	LAM9
253	■■■■■■■■■■■■■■■■■■■■□■■□□■■■■■■■□□□□■■■■■■■	19	ARG(5) DNK(1) FIN(1) FXX(1) IDN(4) NLD(1) POL(1) RUS(3) USA(1) VEN(1)	T1
254	■■■■■■■■■■■■■■□□□□□□□□□□■■■■■■■■□□□□■■■■■■■	92	AUT(10) AZE(2) BEL(2) DEU(2) DNK(1) ESP(1) FIN(1) FXX(1) GEO(9) KAZ(1) LVA(6) NLD(1) POL(1) RUS(50) SAU(1) USA (3)	T5_RUS1
255	□□□□□□□□□□□□□□□□□□□□□□□□□□□□□□□□□□■■■■□■■■■	19	BEL(1) FXX(1) ITA(2) MDG(1) MYS(3) REU(1) THA(3) USA(6) VNM(1)	BEIJING
256	■■■■■■■■■■■■■■■■■■■■■■■■■■■■□□□□■□■■■■□■■■■	54	AUS(1) DNK(1) IDN(2) IND(1) MYS(18) NLD(1) THA(10) USA(20)	EAI5
257	□□□■■■■■■■■■■■■■■■■■■■■■■■■■□□□□■□■■■■□□■■■	9	DNK(9)	EAI5
258	□□□□□□□□□□□□□□□□□□□□■■■■■■■■■■■■□□□□■■■■■■■	5	MEX(1) USA (4)	T1
259	■■■■■■■□■■■■■■■■■■□■■■■■■■■■■■■■■□□□□■■■■■■■	4	GLP(2) MTQ(2)	T1
260	□□□□□□□□□□□□□□□□□□□□□□□□□□□□□□□□□□■■□□■■■■■	6	RUS(2) USA (4)	BEIJING
261	■■■□■■■■■■■■■■■■■■■■■■■■□■■■■■■■□□□□■■■■■■■	2	RUS(2)	T1
262	■■■■■■■□□■■■■■■■■■■■■■■■■■■■□□□■□□□□■■■■■■■	88	ARM(2) AUT(12) AZE(1) BEL(1) CZE(1) EST(3) FIN(8) FXX(1) GEO(5) ITA(11) KAZ(3) LVA(1) NLD(2) POL(3) RUS(24) SWE(1) TUR(3) USA (6)	H4
263	■■■■■■■■■■■□□□□□□□□□□□□□□■■■■■■■□□□□■■■■■■■	5	FIN(1) RUS(4)	T1
264	■■■■■■■■■■■■■□□□□□□□□□□□□■■■■■■■□□□□■■■■■■■	40	DEU(1) GEO(8) LVA(3) POL(6) RUS(17) USA (5)	T1
265	□□□□□□□□□□□□□□□□□□□□□□□□□□□□□□□□□□■■□■■■■■■	26	AZE(1) ISR(5) MYS(1) NLD(2) RUS(7) THA(2) USA (7) VNM(1)	BEIJING
266	■■■■■■■□■■■■■□□□□□□□□□□□□■■■■■■■□□□□■■■■■■■	14	DZA(1) LVA(1) RUS(10) USA (2)	T1
267	■■■■■■■■■■■■□□□□□□□□□□□□□■■■■■■■□□□□■■■■■■■	12	BEL(1) DEU(2) GEO(4) LBY(2) RUS(2) USA (1)	T1
268	■■■■■■■■■■■■■■■■■■■■□■■■■■■■■■□■□□□□■■■■■■■	5	ESP(1) MTQ(1) PER(2) RUS(1)	H3
269	□□□□□□□□□□□□□□□□□□□□□□□□□□□□□□□□□□□□■■■■■■■	38	DEU(2) FXX(1) GEO(1) HTI(2) IDN(1) JPN(1) MEX(1) RUS(8) SAU(1) USA(19) VNM(1)	BEIJING-LIKE
270	□■■□■■■■■■■■■■■■■■■■■■■■■■■■□□□□■□■■■■■□■■■	3	MYS(1) NLD(2)	EAI1_SOM

271	■■■■■■■■□□□□□□□□□□□□□□□□□□□□□□□□□□□□□□■□□■■	2	NLD(2)	U
272	□□□□■■■■■■■■■■■■■■■■■■■■■■■■■■■■□□□□■■■■■■■	6	BEL(1) ITAS(2) USA (3)	T1
273	■■■■■■■■■■■□□□■■■■■■□□□□■■■■■■■■□□□□■■■■■■■	4	ITA(1) ITAS(2) VEN(1)	LAM9
274	■■■■■■■■■■■■■■■■■■■■□□□□□□□□□□□□□□□□□■■■■■■	7	AUT(2) GUF(1) ITAS(1) RUS(1) SAU(1) USA (1)	U
275	■■■■■■■■■■■■■■■■■■■■□□□□□□□□□□□■□□□□■■■■■■■	6	BEL(1) BRA(1) CUB(1) ITAS(2) USA (1)	H3
276	■■■■■■■■■■■■■■■■■■■■■■■■□□□□□□■■□□□□■■■■■■■	29	AUT(5) CZE(1) FXX(1) IND(2) ITAS(1) NLD(1) POL(1) SWE(1) USA(16)	T1
277	■■■■■■■■■■■■■■■■■■■■■■■■■■■■□■■■□□□□■■■□□□□	4	ITA(2) ITAS(2)	T1
278	■■■■■■■■■■■■■■■■■■■■■■■■■■■■■■■■□□□□■■■■■□■	5	FIN(1) ITAS(2) SEN(1) USA (1)	T1
279	■■□■■■■■■■■□■■■■■■■□□■■■■■■■□□□□■□■■■■■■■■■	5	AUS(2) MYS(3)	EAI2_MANILLA
280	■■■■■■□□□□□□□□□□□□■■■■■■■■■■■■■■□□□□■■■■■■■	46	AUS(4) AUT(3) DEU(3) EST(1) FIN(7) GEO(1) LVA(1) POL(4) RUS(5) SWE(1) TUR(2) USA (14)	T1_RUS2
281	■■■■■■■■■■■■■■■■□■■■■■■■■■■■■■■■□□□□■■■■■■■	13	AUS(4) BEL(1) ESP(2) FXX(1) IND(2) NLD(1) PAK(1) SAU(1)	T1
282	■■■■■■■■■■■■■■■■■□■■■■□□■■■■■■■■□□□□■■□□□□■	2	AUS(2)	X2
283	■■■■■■■■■■■■■■■■■■■■■□□□■□□□□□□■□□□□■■■■■■■	30	ARG(3) AUS(3) AUT(7) CZE(2) FXX(1) LVA(6) NLD(2) SWE(2) USA(4)	H1
284	□□□□■■■■■■■□□■■■■■■■■■■■■■■■■■■■□□□□■■■■■■■	35	AUS(1) AUT(10) DEU(4) DNK(1) FIN(1) IRN(1) MEX(1) NLD(2) RUS(1) SAU(4) TUR(8) USA (1)	T1
285	■■□□■■■■■■■■■■■■■■■■■■■■■■■■■■■■□□□□■■■■■■■	3	AUS(1) FXX(1) THA(1)	T1
286	■■□■■■■■■■■■■■■■■■■□□■■■□□□□□□□□□□□□□□□□□□□	8	AUS(1) GLP(1) IDN(1) ITA(1) PHL(2) USA(2)	U
287	■■□■■■■■■■■■■■■■■■■□□■■■■■■■□□□□■□■■■■■■□■■	10	AUS(1) FXX(1) IDN(1) MYS(3) PHL(2) USA (2)	EAI2_MANILLA
288	■■■□□□□□□□■■■■■■■■■■■■□□□□□□□□□□□□■■■■■■■■■	40	AUS(1) BGD(2) DEU(2) GBR(1) IND(8) IRN(1) NLD(5) USA(20)	CAS2
289	■■■□□□□■■■■■■■■■■■■■■■□□□□□□□□□□□□■■■□■■■■■	5	AUS(1) IND(1) NLD(2) USA (1)	CAS1_DELHI
290	■■■■■■■■■■■■■■■■■■■■□□□□■■□■■■■■□□□□■■■■■■■	17	AUS(1) FXX(1) GLP(1) IDN(9) MYS(1) USA (4)	LAM8
291	■■■■■■■■■■■■■■■■■■■■□■■■■■■■■■■■□□□□■■■■■■■	47	ARG(3) AUS(1) BEL(1) BGD(3) BRA(4) CZE(2) EGY(1) ESP(1) GEO(6) HTI(1) IDN(1) IND(1) ITA(5) MDG(1) NAM(1) NLD(1) PER(1) POL(2) RUS(1) TUR(1) USA (1) VEN(1) VNM(1) ZWE(6)	T1
292	■■■■■■■■■■■■■■■■■■■■■■□■■■■■□□□□■□■■□■■■■■■	73	AUS(1) BGD(62) FIN(1) GEO(1) ITAS(1) MYS(3) USA (4)	EAI6_BGD1
293	■■■■■■■■■■■■■■■■■■■■■■■■■■■■■■□■□□□□■■■■□■■	6	AUS(1) FXX(1) PRT(1) USA (3)	H3
294	■□■■■■■■■■■■■■■■■■■■■■■■■■■■■■□■□□□□■■■■■■■	11	AUT(1) DEU(3) GBR(1) HTI(1) IDN(1) MYS(1) NLD(1) USA(2)	H3
295	■■■■■■■■□□■■■■■■■■■■■■■□□■■■■■■■□□□□■■■■■■■	12	GLP(1) HTI(5) USA (6)	S
296	■□□■■■■■■■□■■■□■■■■■■■■■■■■■□□□□■□■■□□□■■■■	2	DNK(2)	EAI3_IND
297	■□□■■■■■■■■■■■□■■■□■■■■■■■■■□□□□■□■■■■□■■■■	2	DNK(2)	EAI5
298	■□□■■■■■■■■■■■□■■■■■■■■■■■■■□□□□■□■■□□□■■■■	26	DNK(23) IND(2) USA(1)	EAI3_IND
299	■□□■■■■■■■■■■■■■■□□□□■■■■■■■□□□□■□□■□□■■■■■	22	DNK(12) MYS(1) NLD(7) NZL(2)	U
300	■□■■■■■■■■■■□■□■■■■■□■■■■□□■□□□□■□■■■■□■■■■	2	DNK(2)	EAI4_VNM
301	■□■■■■■■■■■■■■□■■■■■■■■■■■■■□□□□■□■■■■□□■■■	3	DNK(3)	EAI5
302	■■■■■■■■■■■■■□■■■□■■■■■■■■■■■■■■□□□□■■■■■■■	28	DNK(18) GBR(1) ITA(2) KEN(4) MDG(1) NLD(1) NOR(1)	X1
303	■■■■■■■■■■■■■■□■■■■■■■■■■■■■□□□□■□■■□■■□■■■	2	DNK(2)	EAI1_SOM
304	■□■■□□□□□■■■■■■■■■■■■■□■■■■■■■■■□□□□■■■■■■■	3	USA (3)	T5
305	■■■■■■■■■■■■■■■■■■■□□■■■■■■■■■■■□□□□■■□□■■■	2	USA (2	H37Rv?
306	■■■■■■■■■■■■■■■■■■■■□□□□□□■■□■■■□□□□■■■■■■■	16	BEL(3) CAF(2) FXX(2) GBR(2) GUF(1) USA (6)	T1
307	□□□■■■■■■■■■■■■■■■■■■■■■■■■■■■□■□□□□■■■□■■■	4	CAF(4)	H3
308	■■□■■■■□□□□□□□□□□■■■■■■■■■■■■□■■■■■■■■□□□□□	2	CAF(2)	BOV
309	■■■□□□□■■■■■■■□■■■■■■■□□□□□□□□□□□□■■□□■■■■■	2	CAF(2)	CAS1_DELHI
310	■■■■■■■■■■■■□■□■■■■■■■■■□□□□□□□■□□□□■■■□■□□	3	CAF(3)	H3
311	■■■■■■■■■■■■■■□■■■■□■■■■□□□□□□□■□□□□■■■□■■■	2	CAF(2)	H3
312	■■■■■■■■■■■■■■□■■■■■■■■■□□□□□□□■□□□□■■■□■■■	9	CAF(6) CMR(2) FXX(1)	H3
313	■■■■■■■■■■■■■■■■□□□□□□□□□■■■■■■■□□□□■■■□■■■	2	CAF(2)	T2

314	■■■■■■■■■■■■■■■■■■■□■■■■□□□□□□□■□□□□■■■□■■■	4	CAF(4)	H3
315	■■■■■■■■■■■■■■■■■■■■■■■□■□□□□□□■□□□□■■■□■■■	10	CAF(6) ESP(1) MDG(1) USA(2)	H1
316	■■■■■■■■■■■■■■■■■■■■■■■■□□□□□□□■□□□□■■■□■■■	26	CAF(13) CIV(1) CMR(5) FXX(2) LBY(1) MDG(1) SDN(1) THA(1) USA (1)	H3
317	■■■■■■■■■■■■■■■■■■■■■■■■■■■□■■■■□□□□■■■□■■■	15	BEL(2) CAF(2) CMR(10) FXX(1)	T2
318	■□■■■■□□□■■■■■■■■■■■■■■■■■■■■■■■■■■■■■□■■■■	3	CIV(1) GNB(2)	AFRI_1
319	■□■■■■■□□□□□■■■■■■■■□□□□■■■■■■■■■■■■□□□■■■■	9	CIV(1) CMR(3) POL(1) USA(4)	AFRI_2
320	■■■■■■□□□□□□□□□□■■■■□□□□■■□□■■■■■■■■□□□■■■■	4	CMR(2) USA(2)	AFRI
321	■■■■■■□□□■■■□■■■■■□□□□□□■□□□□■■■■■■■■■□■■■■	2	CIV(2)	AFRI_1
322	■■■■■■□□□■■■■■□■■■■■■■■■■■■■■■□■■■■■■■□■■■■	3	MRT(2) SEN(1)	AFRI_1
323	■■■■■■□□□■■■■■□■■■■■■■■■■■■■■■■■□■■■■■□■■■■	3	FXX(1) SEN(2)	AFRI_1
324	■■■■■■□□□■■■■■□■■■■■■■■■■■■■■■■■■■■■■■□■■■■	5	FXX(3) SEN(2)	AFRI_1
325	■■■■■■□□□■■■■■■■■■■□■□□□■■■■■■■■■■■■■■□■■■■	6	FXX(2) ITA(1) USA(3)	AFRI_1
326	■■■■■■□□□■■■■■■■■■■■■□□□■■■■■■■■■■■■■■□■■■■	26	BEL(2) DEU(2) FXX(4) GIN(1) GLP(1) GNB(2) ITA(2) MTQ(1) NLD(6) USA (5)	AFRI_1
327	■■■■■■■□□□□□■■□■■■■■□□□□■■□■■■□■□■■■□□□■■■■	3	CMR(3)	AFRI_2
328	■■■■■■■□□□□□■■□■■■■■□□□□■■□■■■■■□■■■□□□■■■■	9	CMR(8) SEN(1)	AFRI_2
329	■■■■■■■□□□□□■■□■■■■■□□□□■■■■■■■■■■■■□□□■■■■	5	BEL(1) CIV(1) CMR(3)	AFRI_2
330	■■■■■■■□□□□□■■■■■■■■□□□□■■■■■■■■■■■■□□□□■■■	3	BEL(1) CAF(1) CMR(1)	AFRI
331	■■■■■■■□□□□□■■■■■■■■□□□□■■■■■■■■■■■■□□□■■■■	18	BFA(1) CIV(3) CMR(3) NGA(1) NLD(1) USA (9)	AFRI_2
332	■■■■■■■■□■■■□□□■■■■■■■■■■■■■■■□■■■■■□□□■■■■	5	CMR(5)	AFRI
333	■■■■■■■■■■■■□■□■■■■■□□□□■■■■■■■■□□□□■■■■■■■	10	GLP(1) GUF(1) VEN(8)	LAM5
334	■□■■■■■■■■■■■■■■■■■■■■■■■■■■■■■■□□□□■■■■■■■	39	BEL(2) ESP(2) FIN(2) FXX(2) GBR(2) GEO(3) GLP(1) GNB(3) GUF(3) HTI(1) IND(1) ITA(5) JPN(1) MTQ(1) NLD(1) SEN(1) SWE(1) USA (5) VNM(2)	T1
335	■■■■■■■■■■■■■■■■■■■■□□□□■■■■■■□■□□□□■■■■■■■	27	AUT(3) CZE(5) ESP(1) FXX(4) ITA(4) MTQ(2) ROM(1) RUS(3) SWE(1) USA (2) VNM(1)	H3-LAM9
336	■■■■■■■■■■■■■■■■■□■■■■■■■■■■■■■■□□□□■■■□■■■	37	DNK(1) GBR(5) ITA(2) MDG(1) MEX(2) RUS(2) SWE(5) USA(12) ZAF(7)	X1
337	■■■■■■■■■■■■■■■■■■■■■■■■■■■□□□□□■□■■■■■□■■■	3	FXX(1) KEN(2)	EAI1
338	□□□■■■■■■■■■■■■■■■■■■■■■■■■■■■□□□□■□■■□□□■■■■	7	BGD(1) GBR(5) MYS(1)	EAI3_IND
339	■□□□■■■■■■■■■■■■■□■■■■■■■■■■■■■■□□□□■■□□□□■	16	GBR(16)	X2
340	■□□■■■■□□□■■■■■■■■■■■■■■■■■■□□□□■□■■■■■■■■■	21	AUS(1) DNK(2) FXX(1) GBR(2) IND(6) MYS(1) NLD(1) USA (7)	EAI5
341	■□□■■■■■■■■■■■■■■■■■■■■■■□■■□□□□■□■■□□□□■■■	2	GBR(2)	EAI5
342	■■□■■■■■■■■■■■■■■■■■■■■■■■■■□□□□■□■■■■■■■■■	8	BEL(1) DEU(1) DNK(1) GBR(2) NLD(1) USA(2)	EAI5
343	■■■□□□□□□□□□□□□□□□□□□□□□■■■□□■■■■■□■■■■■■■■	5	ETH(2) GBR(2) (1) USA	U
344	■■■□□□□□□□□□■■■■■■■■■■■■■■■■■■■■□□□□■■■■■■■	7	GBR(2) GUF(1) PRT(4)	T1
345	■■■■■■■■■□□□□□□□□□□■■■■■■■■■■■■■□□□□■■■□■■■	10	FIN(1) GBR(4) KEN(2) NOR(1) USA (2)	T3_ETH
346	■■■■■■■■■□■■■■■■■■■■■□□□□□□□□□□□□□■■■□■□■■■	2	GBR(2)	U
347	■■■■■■■■■■■■□■■■■□■■■■■■■■■■■■■■□□□□■■□□□□■	16	GBR(2) GUF(1) ITAS(1) NZL(2) USA(8) ZAF(2)	X2
348	■■■■■■■■■■■■□■■■■□■■■■■■■■■■■■■■□□□□■■□■■■■	7	GBR(2) USA(1) ZAF(4)	X1
349	■■■■■■■■■■■■□■■■■■■■■■■■■■■■□□□□■□■■■■■□■■■	11	BGD(1) GBR(2) USA (8)	EAI1_SOM
350	■■■■■■■■■■■■■■■■■□■■□□□■■■■■■■■■□□□□■■□□□□■	5	AUT(1) GBR(4)	X2
351	■■■■■■■■■■■■■■■■■□■■■■■■■■■■■□□□□□□□□□■■■■■	19	GBR(14) NLD(4) USA(1)	U
352	■■■■■■■■■■■■■■■■■□■■■■■■■■■■■■■■□□□□■■■□□■■	11	GBR(2) IDN(1) NZL(2) USA (6)	X1
353	■■■■■■■■■■■■■■■■■■■■■■■■■□□■■■■■□□□□■■■■■■■	11	AUT(3) GBR(2) NLD(2) USA (4)	T1
354	■■■■■■■■■■■■■■■■■■■■■■■■■■■■□□□□□□■■■■■□■■■	6	AUS(1) GBR(2) NLD(1) SWE(1) THA(1)	U
355	■□□■■■■■■■■■■■■■■■■■■■■■■■■■□□□□■□■■□□□□■■■	20	DNK(1) FXX(1) GBR(5) IND(1) MUS(1) MYS(2) NLD(1) USA (8)	EAI5
356	■■■□□□□■■■■■■■■■■■■■□□□□□□□□□□□□□□□■■■■■■■■	6	GBR(2) SAU(1) SDN(2) USA (1)	CAS
357	■■■□□□□■■■■■■■■■■■■■■■□□□□□□□□□□□□□□■■■■■■■	30	BEL(2) BGD(11) DNK(1) GBR(2) IND(2) IRN(2) NLD(2) SAU(2) SWE(3) USA (3)	CAS

358	■■■□□■■■■■■■■■■■■■■■■■■■■■■■■■■■□□□□■■■■■■■	11	BGD(6) FIN(1) GBR(2) ITA(1) MTQ(1)	T1
359	□□□□■■■■■■■□■■■■■■■■■■■■■□□□□□□■□□□□■■■■■■■	2	AUT(2)	H1
360	■□■■□□□□□□■■■■■■■■■■■■■■■■■■■■□■□□□□■■■■■■■	4	AUT(4)	H3
361	■□■■■■■■■■■■□■■■■■■■■■■■■■■■■□□□■□□□□■■■■■■■	4	AUT(2) IRN(1) TUR(1)	H4
362	■■□■■■■□■■■■■■■■■■■■■■□■■■■■■■■■□□□□■■■■■■■	18	AUT(18)	T5
363	■■■□□■■■□□■■■■■■■■■■■■■■■■■■■■■■□□□□■■■■■■■	7	AUT(3) EGY(2) FXX(1) ITAS(1)	S
364	■■■■□■■■■■■■■■■■■■■■■■■■■■□■■■■■□□□□■■■■■■■	3	AUT(3)	T1
365	■■■■■■■■■□□□□□□□□□□□□□□□□■■■■■■■□□□□■■■■■■■	7	AUT(6) RUS(1)	T1
366	■■■■■■■■■■□■■■■■■■■■■■■■■■■■■■■■□□□□■□□■■■■	10	AUT(10)	T1
367	■■■■■■■■■■■■□■■■■■■□□□□□■□□■■■■■□□□□■■■■■■■	5	AUT(2) DNK(1) NLD(1) TUR(1)	LAM7_TUR
368	■■■■■■■■■■■■■■■■■■□□□□□□■■■■■■□■□□□□■■■□■■■	2	AUT(2)	H3
369	■■■■■■■■■■■■■■■■■■■□■■■■■□□□□□□■□□□□■■■■■■■	3	AUT(2) DEU(1)	H1
370	■■■■■■■■■■■■■■■■■■■■■■□□■■■■■■■■□□□□■□□■■■■	8	AUT(5) EST(1) SWE(1) USA (1)	T1
371	■■■■■■■■■■■■■■■■■■□□□□□□■■■■■■□■□□□□■■■■■■■	9	AUT(7) CZE(1) USA (1)	H3
372	■■■■■■■■■■■■■■■■■■■■■■□■□■□□□□□□□□□□□□□□□□□	2	AUT(2)	U
373	■■■■■■■■■■■■■■■■■■■■■■■□■■■■■■■■□□□□■■■■■■■	16	AUT(3) BEL(1) CZE(3) FIN(1) FXX(1) NLD(1) USA(3) ZAF(3)	T1
374	■■■■■■■■■■■■■■■■■■■■■■■■■■□□□□□□□□□□■■■■■■■	6	AUT(5) FXX(1)	U
375	□□□■■■■■■■■■□■■■■■■■□□□□■■■■■■■■□□□□■■■■■■■	3	VEN(3)	LAM5
376	□■■■■■■■□□□■■■■■■■■■□□□□■■■■■■■■□□□□■■■■■■■	23	BEL(1) USA (16) VEN(6)	LAM3
377	■■□■□■■■■■■■□■■■■■■■□□□□■■■■■■■■□□□□■■■■■■■	6	USA(2) USA (2) VEN(2)	LAM2
378	■■■■■■■■■■■■■■■■■■■■□■■□■■■■■■■■□□□□■■■■■■■	3	GEO(1) VEN(2)	T1
379	■■■■■■■■■■■■■□□□□■□□□□□■■■■■■■■■□□□□■■■■■■■	15	ARG(12) BRA(1) FXX(1) VEN(1)	T1
380	■□■■■■■■■■■■■■■■■■■■■■■■■■■■□□□□■□■■■■■■■■■	2	AUS(1) AUT(1)	EAI5
381	■■■□□□□■■■■■■■■■■■■■■■□□□□□□□□□□□□■■□□□■■■■	9	AUT(1) BGD(1) GBR(1) IND(2) NLD(2) PAK(1) USA(1)	CAS1_DELHI
382	■■■■□■■■■■■■■■■■■■■■■■■■■□□□□□□■□□□□■■■■■■■	8	AUT(3) EST(1) FXX(1) POL(1) USA (2)	H1
383	■■■■■■■■□□■■■■■■■■■■■■■■■□□□□□□■□□□□■■■■■■■	9	AUT(2) BRA(1) DEU(1) FIN(3) ITA(2)	H1-S
384	■■■■■■■■■■■■■■■■■■■■□■■■■□□□□□□■□□□□■■■■■■■	4	AUT(1) CZE(1) FXX(1) USA(1)	H1
385	■■■■■■■■■■■■□■■■■□□■■■■■■■■■■■■■□□□□■□■■■■■	3	GUF(2) MTQ(1)	T3
386	□□□□□□□□□□□□□□□□□□□□□□□□■■■□■■■■□□□□■■■■■■■	3	BRA(3)	T1
387	■□□□□□□□□□□□□□■■■■■■□□□□□□□■□■■■□□□□■■■□■■■	2	BRA(2)	T2
388	■■■□■■■■■■■■■■■■■■■■□□□□■■■■■■■■□□□□■■■■■■■	15	BRA(2) DZA(8) JPN(2) PRT(1) RUS(1) VEN(1)	LAM9
389	■■□■■■■■■■■■■■□■■■■■□□□□■■■■■■■■□□□□■■■■■■■	14	BRA(4) FIN(1) PRT(7) VEN(2)	LAM1
390	■■■■■■■■■■■■■■■■■■■■■■■■■■■■■□□■□□□□■■■■■■■	16	AUT(2) BRA(2) DEU(1) EGY(1) GLP(1) ITA(2) MTQ(3) SWE(1) USA(3)	H3
391	■■■■□□□■■■■■■■■■■■■■□□□□■■■■■■■■□□□□■■■□■■■	2	BRA(2)	LAM4
392	■■■■■■■■■■■■■■■■■■■■□□□□□□□□□■■■□□□□■■■□■■■	3	ARG(1) BRA(2)	T2
393	■■■■■■■■■■■■■□■■■■■■■■■■■■■■■■■■□□□□■■■■■■■	16	ARG(1) CHN(1) DNK(1) EGY(1) EST(1) FXX(3) GEO(1) ITA(1) SWE(1) USA(4) VNM(1)	T1
394	■■■■■■■■■■■■■■■■■■■■■■■■■■■■■□□■□□□□■■■□■■■	2	CUB(1) VNM(1)	H3
395	■■■■■■■■■■■■■■■■■■■■■■■■■■■■■■■■□□□□■□□□□□□	5	ESP(1) USA (4)	T1
396	■■■■■■■■■■■■■■■■■■■■□□□□■■■■□■■■□□□□□■■■■■■	10	BRA(7) ITA(2) PRT(1)	U
397	■■■■■■■■■■■■■■■■■■■■□□□□□□□□□□□□□□□□■■■■■■■	12	ARG(1) BRA(2) ITAS(1) USA (4) VEN(4)	U
398	■■■■■■■■■■■■■■■■■■■■□□□□■■■■■■■■□□□□■■□□■■■	9	AUT(1) BRA(2) CUB(1) MEX(2) USA(3)	LAM9
399	■■■■■■■■■■■■■■■■■■■■■■■■■■■■□□□■□□□□■□□□□■□	2	BRA(2)	H4
400	■■■■□■■■■■■■■■■■■■■■■■■■■□□□□□□■□□□□■■■□■■■	2	AUT(1) VEN(1)	H1
401	■■■■■■■■□□■■■■■■■■■□□□□□□■■■■■■■□□□□■■■■■■■	5	AUS(1) BRA(4)	S
402	■■■■■■■■■■■■■■■■■■■■□□□□□□□□□□□□□□□□□□□□□□□	14	AUS(1) AUT(1) BRA(3) DZA(1) FXX(1) GEO(3) PRT(1) RUS(1) USA(2)	U

403	■■■■■■■■■■■■■■■■■■■■■■□□□■■■■■■■□□□□■■■□■■■	17	AUT(1) CMR(12) FXX(1) IND(2) USA (1)	LAM10_CAM
404	■■■■■■■■■■■□■□□■■■□□□□□□□□□□□□□□□□□□□□□□□■■	2	USA (2)	ZERO
405	■■■■■■■■■■■■■■■■■■□□□□□□□□□□□□□□□□□□□□□□□■■	12	DNK(1) USA(3) VNM(8)	ZERO
406	□□□□□□□□□□□□□□□□□□□□□□□□□□□□□□□□□□□□■■■□■■■	10	IND(1) ITA(1) USA (8)	BEIJING-LIKE
407	□□□□□□□□□□□□□■■■■□□□□■□□□□□□□□□□□□□■■■■■■■■■	2	USA (2)	CAS
408	□□■■□■■■■■■■■□■■■□■■■■■■■■■■■■■■□□□□■■■■■■■	3	USA (3)	X1
409	□■□■■□■□□□□□■■■□■■■■■■■■■■■■■■■■■■■■■■□□□□□	31	ARG(5) GBR(3) USA (23)	BOVIS2
410	□■■■■■■■■■■■■■■■□□□□□■■■■■■■■■■■□□□□■■■■■■■	3	USA (3)	T1
411	■□□□□□■■■■■■■■■■■■■■■■■■■■■■■■■■□□□□■■■■■■■	8	FXX(1) MWI(1) USA (6)	T1
412	■□□□■■■■■■■■■■■■■■■■□□□□■■□□□□■■□□□□■■□□□□□	2	USA (2)	LAM11_ZWE
413	■□□■■■■■■■■■■■■■■■■□□■■■■■■■□□□□■□■■■■■■■■■	10	ITA(1) PHL(3) USA (5) VNM(1)	EAI5
414	■□□■■■■■■■■■■■■■■■■■■■■□■■■■□□□□■□■■□□□■■■■	12	GUF(1) IND(1) MYS(1) NLD(3) USA (6)	EAI3_IND
415	■□□■■■■■■■■■■■■■■■■■■■■■■□□□□■□■■■■■■■■■■■■	14	IND(6) NZL(3) USA (5)	U
416	■■□□□■■■□■■■■■■■■■■■□■■■■■■■■■■■■■■■■■□□□□□	7	CAN(7)	BOV
417	■□■■■■■■■■■■■■■■■■■■■■■■□□□□□□■■□□□□■■■■■■■	2	USA (2)	T1
418	■■□□□■■■■■■■■■■■■■■■■■■■■■■■■■□■□□□□■■■■■■■	5	ARG(1) USA (4)	H3
419	■■□□■■□□□□□□□□□□□□□■□□□□■■■■■■■■□□□□■■■■■■■	4	USA (4)	LAM9
420	■■□□■■■■■■■■■■■■□□■■■■■■■■■■■■■■□□□□■■■□■■□	12	BEL(4) EAFR(1) UGA(1) USA (6)	T2
421	■■□■■■■■□■■□□□□□□□□□□□□□□□□□□□□□□□□□■■■■■■	12	USA (12)	U
422	■■□■■■■■□■■■■■■□■■■■□■■□■■■■■■■■■■■■■■□□□□□	7	USA (7)	BOVIS1
423	■■□■■■■■■■■■■■■■□□□■□□□□■■■■■■■■□□□□■■■■■■■	7	BEL(3) USA (4)	LAM1
424	■■□■■■■■■■■■■■■■■■■□□■■■■■■■□□□□■□■■■■■□□■■	9	USA(8) USA (1)	EAI2_MANILLA
425	■■■□□□□□□□□□■■■■■□■■■■■■■□□□□■■■□□□□■■■■■■■	3	USA (3)	X3
426	■■■□□□□□□□■■■■■■■■■■□□□□□□□□□□□□□□■■■■■■■■■	2	USA (2)	CAS
427	■■■□□□□■■■■■□□□■■■■■■■□□□□□□□□□□□□■■■■■■■■■	6	DEU(1) IND(1) USA (4)	CAS1_DELHI
428	■■■□□□□■■■■■■■■■■■■■■■□□□□□□□□□□□□■■□■■■■■■	8	IND(2) PAK(1) USA (5)	CAS1_DELHI
429	■■■□□□□■■■■■■■■■■■■■■■□□□□□□□□□□□□■■■■■□■■■	9	GBR(1) IND(1) SDN(1) USA(6)	CAS1_DELHI
430	■■■□□□■□□□□□□□□□■■□■■■□□■■■■■■■■□□□□■□□■■■■	9	USA (7) VEN(2)	T4-CEU1
431	■■■□□■■■■■■■■■■■■■■■■■■■■□□□□□□■□□□□■■■■■■■	3	USA (3)	H1
432	■■■□■■■□■■■■■■■■■■■■■■■■■■■■■■■■□□□□■■■□■■■	3	USA (3)	T2
433	■■■■□■■■■■■■■■■■■■■■■■■■■■□■■□■□□□□■■■■■■■	16	BEL(1) ESP(1) USA (14)	H3
434	■■■■■□□□□■■■■■■■■■■■■■■■■■■■■■■□□□□□□■■■■■■	5	USA (5)	U
435	■■■■■□□■■■■■■■■■■■■■□□□□■■■■■■■■□□□□■■■■■■■	4	USA (4)	LAM9
436	■■■■■■□□□□■■■■■■■□■■■■■■■■■■■■■■□□□□■■□□□□■	11	USA (11)	X2
437	■■■■■■■□□□□□■■■■■■■■■■■■■■■■■■■■■■□■□□□■■■■	4	CMR(1) SLE(1) USA (2)	AFRI_3
438	■■■■■■■□□□□□■■■■■■■■■■■■■■■■■■■■■■■■□□□■■■■	5	NGA(2) SLE(1) USA (2)	AFRI_3
439	■■■■■■■■■□■■■■■■■□■■■■■■■■■■■■■■□□□□■■□□□□■	2	USA (2)	X2
440	■■■■■■■■■■■□□□□■■■■■□□□□■■■■■■■■□□□□■■■■■■■	11	GNB(1) USA(10)	LAM9
441	■■■■■■■■■■■■□□□□□□□■□■■■■■■■■■■■□□□□■■■■■■■	2	USA (2)	T1
442	■■■■■■■■■■■■□■■■■■□■■■■■■■■■■■■■□□□□■■■■■■■	4	USA (4)	T3
443	■■■■■■■■■■■■□■■■■■■□□□□■■■■■■□□□□□□■■■■■■■	41	BEL(3) BRA(1) USA (36) VEN(1)	U
444	■■■■■■■■■■■■■□□□□□□□□□□□□□■■■■■■□□□□■■■■■■■	23	AUT(3) DEU(1) GEO(1) KAZ(1) RUS(1) USA(16)	T1
445	■■■■■■■■■■■■■□■■■□■■■■■■■■■■■■■■□□□□■■□□□□■	9	USA (9)	X2
446	■■■■■■■■■■■■■■□□□□□□□□□■■■□■■■■■□□□□■■□□□□■	18	USA(1) USA (17)	T1
447	■■■■■■■■■■■■■■□□□□□■■■■■■■■■■■■■□□□□■■■■■■■	5	AUT(1) USA (4)	T1

448	■■■■■■■■■■■■■■□■■■■■■■■■■■■■■■□■□□□□■■■□■■■	10	CMR(2) FXX(1) PRT(2) USA (5)	H3
449	■■■■■■■■■■■■■■■■■□■■■■□■■■■■■■■■■□□□□■■■■■■■	6	USA(6)	X1
450	■■■■■■■■■■■■■■■■■□■■■■■■□□□□□□□□□□□□□□□□□□□	57	CMR(19) CZE(3) DNK(1) FXX(1) ITA(1) MEX(5) NLD(1) USA(26)	U
451	■■■■■■■■■■■■■■■■■■■□□■■■■■■■■■■■□□□□■■■■■■■	115	?(1) ARG(2) AUT(1) FXX(2) GBR(5) GLP(1) IND(1) NAM(2) NLD(1) PRT(2) RUS(1) SAU(4) SEN(1) USA(86) VEN(4) VNM(1)	H37Rv
452	■■■■■■■■■■■■■■■■■■■■□□□□■■□□■■■■□□□□■■■■■■■	7	USA (7)	LAM9
453	■■■■■■■■■■■■■■■■■■■■□■■■■■■■□■■■□□□□■■■■■■■	7	GUF(1) PRT(1) SWE(1) USA (4)	T1
454	■■■■■■■■■■■■■■■■■■■■■□■■■■□■■■■■□□□□■■■□■■■	4	USA(3) USA (1)	T2
455	■■■■■■■■■■■■■■■■■■■■■■■□□□□□■■■■□□□□■■■■■■■	14	HTI(6) USA (8)	T1
456	■■■■■■■■■■■■■■■■■■■■■■■■■□□■□□□□■□■■■■■□□■■	3	USA (3)	EAI4_VNM
457	■■■■■■■■■■■■■■■■■■■■■■■■■■■□■■□■□□□□■■■■■■■	12	ESP(1) FIN(1) HTI(1) ITA(1) USA (8)	H3
458	■■■■■■■■■■■■■■■■■■■■■■■■■■■■□□□□□□■■■■■■■■■	17	DNK(1) GNB(2) IND(1) THA(8) USA (3) VNM(2)	U
459	■■■■■■■■■■■■■■■■■■■■■■■■■■■■□□□□■□□□□■■□■■■	6	FXX(1) USA(5)	U
460	■■■■■■■■■■■■■■■■■■■■■■■■■■■■□□□□■□■■■■■■□■■	2	USA (2)	EAI5
461	■■■■■■■■■■■■■■■■■■■■■■■■■■■■□□□□■■■□■■■■■□■	57	IND(1) NZL(2) USA (54)	U
462	■■■■■■■■■■■■■■■■■■■■■■■■■■■■□■■■□□□□■■■■■■■	23	AUT(1) BRA(2) CMR(1) ETH(1) FIN(2) FXX(1) ITA(3) POL(3) PRT(1) RUS(1) USA (7)	T1
463	■■■■■■■■■■■■■■■■■■■■■■■■■■■■■■□■□□□□■□■■■■■	10	ARG(2) AUT(1) BRA(2) POL(2) USA (3)	H3
464	■■■■■■■■■■■■■■■■■■■■■■■■■■■■■■■□□■■■■■■■■■■	3	USA (3)	U
465	■■■■■■■■■■□□□□■■■■■■■□■■■■■■■■□□□□□□□□□□□□□	2	USA (2)	U
466	■■■■■■■■□□■■■■■■■■□■■■■■■■■■■■■■■□□□□■■■■■■■	15	BEL(1) DEU(1) DZA(9) FXX(1) MDG(1) NLD(1) TUR(1)	S
467	□□□□□□□□□□□□□□□□□□□□□□□□□□□□□□□■□□□□■■■■■■■	5	LBY(1) USA(4)	H3
468	□□□■■□■■■■■■■■■■■■■■■■■■■■■■□□□□■□■■□□□□■■■	2	AUS(1) USA(1)	EAI5
469	□□□■■■■■■■■■■■■■■■■■□□□□■■■■■■■■□□□□■■■■■■■	21	EGY(2) FXX(1) HTI(1) ITA(2) PER(3) THA(1) USA (11)	LAM9
470	□□■■■■■■■■■■■■■■■■■■■■■■■■■■□□□□■□■■■■■■■■■	3	FXX(1) USA (2)	EAI5
471	□■■□□□□■■■■■■■■■■■■■■■□□□□□□□□□□□□■■■■■■■■■	6	BGD(2) GBR(1) IND(1) USA (2)	CAS1_DELHI
472	□■■■■■■■■■■■■■■■■■■■■■■■■■■■■■□■□□□□■■■■■■■	6	BEL(1) CZE(1) ESP(1) FXX(1) NLD(1) USA (1)	H3
473	■□□□□□□□■■■■■■■■■■■■■■■■■■■■□□□□■□■■□□□■■■■	7	AUS(1) DEU(1) IND(1) MYS(1) USA (3)	EAI3_IND
474	■□□■■□■■■■■■■■■■■■■■■■■■■■■■□□□□■□■■□□□□■■■	2	GBR(1) USA (1)	EAI5
475	■□□■■■■■■■■■■■■■■□□□□■■■■■■■□□□□■□□■■■■■■■■	4	GBR(1) IND(1) USA (2)	U
476	■□■■■■■■■■■■■■■■■□■■■■■■■■■■■■■■□□□□■■□□□□■	2	GBR(1) USA (1)	X2
477	■□■■■■■■■■■■■■■■■■■■■■■■■■■■□□□□■□■■■■■□■■■	10	AUS(1) DNK(3) IND(1) MYS(1) NLD(1) USA (3)	EAI1_SOM
478	■■□□□■■■■■■■■■■■■□■■■■■■■■■■■■■■□□□□■■□□□□■	16	GBR(1) MEX(2) NOR(1) USA(12)	X2
479	■■□■□□□□□□□□□□■□■■■■■■■■■■■■■■■■■■■■■■□□□□□	97	ARG(32) AUS(1) BRA(4) FXX(12) GBR(7) IRL(29) MEX(3) USA (8) ZAF(1)	BOVIS3
480	■■□■■■■□□■■■■■■■■■■□□■■■■■■■□□□□■□□■■■■■■■■	3	USA (3)	U
481	■■□■■■■■□■■■■■■□■■■■□■■■■■■■■■■■■■■■■■□□□□□	414	?(2) ARG(40) BEL(17) BRA(16) DEU(32) ESP(14) FXX(149) GBR(2) ITA(1) MEX(8) NLD(50) SAU(1) SWE(3) URY(1) USA(4) ZAF(74)	BOVIS1
482	■■□■■■■■□■■■■■■□■■■■■■■■■■■■■■■■■■■■■■□□□□□	646	?(7) ARG(15) AUT(12) BEL(5) BRA(10) CHN(1) CZE(1) DEU(36) DNK(30) ESP(12) FXX(342) GBR(7) GLP(3) GUF(1) IRN(5) ITA(26) JPN(1) LKA(1) MEX(3) NAM(1) NLD(43) NOR(1) NZL(2) PRT(2) RUS(4) SWE(29) USA(39) VEN(1) ZAF(4) ZMB(2)	BOVIS1_BCG
483	■■□■■■■■■■■■■■■■■■■□□■■■■■■■□□□□■□■■■■■□□□■	11	FXX(1) GUF(1) MYS(1) PHL(2) USA (6)	EAI2_MANILLA
484	■■■□□□□□□□□□□■■■■□■■■■■■■■■■■■■□□□□□□□■■■■■	2	USA (1) USA (1)	U
485	■■■□□□□■■■■■■■■■■■■□□□□□□□□□□□□□□□■■■■■■■■■	14	AUT(1) IRN(7) USA (6)	CAS
486	■■■□□□□■■■■■■■■■■■■■■■□□□□□□□□□□□□□□□■■■■■■	11	AUT(1) IRN(3) PAK(1) SAU(2) USA (4)	CAS
487	■■■■□□□□□□□□□□□□□□□□□□□□■□□□□□□■□□□□■■■■■■■	10	AUT(1) CZE(5) DEU(1) USA (3)	H3
488	■■■■■□□□□■■■■■■■■■■■■■■■■□□□□□□■□□□□■■■■■■■	5	FXX(1) ITA(1) USA (3)	H1
489	■■■■■□■■■■■■■■■■■□■■■■■■■■■■■■■■□□□□■■□□□□■	3	AUS(1) USA (2	X2

490	■■■■■□■■■■■■■■■■■□■■■■■■■■■■■■■■□□□□■■■■■■■	4	GBR(1) USA(3)	X1
491	■■■■■■□■■■■■■■■■■□■■■■■■■■■■■■■■□□□□■■□□□□■	2	AUS(1) USA(1)	X2
492	■■■■■■□■■■■■■■■■■■■■□□□□■■■■■■■■□□□□■■■■■■■	8	GUF(1) HTI(2) ITA(1) NLD(2) USA (2)	LAM9
493	■■■■■■■□□□□■■■■■■■■■■■■■■■■■□□□□■□■■■■■□■■■	5	MYS(1) NLD(3) USA (1)	EAI1_SOM
494	■■■■■■■■□□■■■■■■■■■■■■■■■■■■■■■■□□□□■■■□□■■	3	ITAS(1) USA (2)	S
495	■■■■■■■■□■■■■■□□□□□□□□□□□■■■■■■■□□□□■■■■■■■	5	AZE(2) RUS(2) USA (1)	T1
496	■■■■■■■■■■□□■■□□□□□□□□□□■■■■■■■■□□□□■■■■■■■	6	GEO(1) RUS(4) USA (1)	T5_RUS1
497	■■■■■■■■■■■□■■■■■■■■■■■■■■■■□□□□■□■■■■■■■■■	4	USA (1) VNM(3)	EAI5
498	■■■■■■■■■■■□■■■■■■■■■■■■■■■■■■■■□□□□■■■■■■■	5	AUT(1) CZE(1) DEU(1) GBR(1) USA (1)	T1
499	■■■■■■■■■■■■□□□□□□□□□□□□□□□□□□■■□□□□■■■■■■■	3	RUS(2) USA (1)	T1
500	■■■■■■■■■■■■□□□□□□□□□□□□□□■■■■■■□□□□■■■■■■■	7	FIN(1) POL(1) RUS(4) USA (1)	T1
501	■■■■■■■■■■■■□□□□□□□■■■■■■■■■■■■■□□□□■■■■■■■	5	CAN(2) GBR(1) USA (2)	T1
502	■■■■■■■■■■■■□■□■■■■■■□■■■■■■■■■■□□□□■■■■■■■	2	FXX(1) USA (1)	T3
503	■■■■■■■■■■■■□■■■■■□■■■■■■■■■■■■■□□□□■■■□■■■	3	CZE(1) GLP(1) USA (1)	T2-T3
504	■■■■■■■■■■■■□■■■■■■■■□■■■■■■■■■■□□□□■■■■■■■	7	FXX(1) ITAS(1) USA (5)	T3
505	■■■■■■■■■■■■□■■■■■■■■■■■■■■■■■■■□□□□■■■□□□□	10	BRA(1) FXX(1) HTI(4) ITA(1) USA (3)	T3
506	■■■■■■■■■■■■■■□□□□□□□□□□□■■■■■■■□□□□■■■■■■■	3	MEX(1) RUS(1) USA (1)	T1
507	■■■■■■■■■■■■■■■■■□□□□□■■■■■■■■■■□□□□■■■■■■■	2	USA (1) USA (1)	T1
508	■■■■■■■■■■■■■■■■■□□■■■■■■■■■■■■■□□□□■■■■■■■	5	GBR(1) USA(4)	T1
509	■■■■■■■■■■■■■■■■■■■■□□□□■□■■■■■■□□□□■■■■■■■	3	FXX(1) USA (2)	LAM9
510	■■■■■■■■■■■■■■■■■■■■■□□□□□□□□□□□□□■■■□■□■■■	2	USA (1) USA (1)	U
511	■■■■■■■■■■■■■■■■■■■■■□□□□□□□□□□■□□□□■■■■■■■	19	CZE(4) DEU(2) FIN(1) FXX(5) POL(3) RUS(1) USA (3)	H3
512	■■■■■■■■■■■■■■■■■■■■■□□□■■■■■■□■□□□□■■■■■■■	8	AUT(1) DEU(1) HUN(1) USA (5)	H3
513	■■■■■■■■■■■■■■■■■■■■■□□■■■■■■■■■□□□□■■■□■■■	5	CZE(2) ESP(1) GBR(1) USA (1)	T2
514	■■■■■■■■■■■■■■■■■■■■■■■■■□□■□□□□■□■■■■■□■■■	4	USA (4)	EAI1_SOM-EAI4
515	■■■■■■■■■■■■■■■■■■■■■■■■■■□■■■■■□□□□■■■□■■■	8	ITA(2) NLD(1) SWE(1) USA (4)	T2
516	■■■■■■■■■■■■■■■■■■■■■■■■■■■□■■■■□□□□■■■■■■■	7	DZA(1) FXX(1) IDN(1) ITA(1) RUS(1) USA (2)	T1
517	■■■■■■■■■■■■■■■■■■■■■■■■■■■■□□□□■□■■■■■□□■■	5	USA (3) USA (1) VNM(1)	EAI5
518	■■■■■■■■■■■■■■■■■■■■■■■■■■■■■□■■□□□□□□□□■■■	2	USA (1) USA (1)	U
519	■■■■■■■■■■■■■■■■■■■■■■■■■■■■■■■□□□□□□■■■■■■	6	GBR(1) NAM(2) PRT(1) USA (1) ZAF(1)	U
520	■■■■■■■■■■■■■■■■■■■■■■■■■■■■■■■■□□□□■□■■■■■	14	AUT(1) BEL(3) CZE(3) DEU(1) FXX(1) MTQ(1) PAK(1) RUS(1) USA (2)	T1
521	■■■■■■■■■■■■■■■■■■■■■■■■■■■■■■■■□□□□■■□□□■■	8	ARG(2) AUT(1) BRA(1) GNB(1) USA (1) ZAF(2)	T1
522	■■■■■■■■■■■■■■■■■■■■■■■■■■■■■■■■□□□□■■■■■■□	8	AUT(1) GNB(1) HTI(1) IDN(2) KEN(1) USA (2)	T1
523	■■■	19	AUT(1) BEL(1) FXX(1) GLP(1) IDN(1) JPN(1) MYS(3) THA(2) USA (4) VNM(2) ZAF(2)	U
524	■■■■■■■■■■■■■■■■■■■■■■■■■■■■■■□■□□□□■■■□□■■	6	AUT(3) ITAS(1) NLD(1) USA (1)	H3
525	■□■■■■□□□■■■■■■■■■■■□□■■■■■■■■■□□□■■■■□■■■■	2	GNB(2)	AFRI_1
526	■■□■■■■■■■■■■■■■■■■■■■■■■■■■□□□□□□■■■■□■■■■	2	GNB(2)	U
527	■■□■■■■■■■■■■■■■■■■■■■■■■■■■□□□□□□■■■■■■■■■	7	GNB(7)	U
528	■■□■■■■■■■■■■■■■■■■■■■■■■■■■□□□□■□■■■■□■■■■	5	GNB(3) USA(2)	EAI5
529	■■□■■■■■■■■■■■■■■■■■■■■■■■■■□□□□■□■■■■■□■■■	4	FXX(1) GNB(3)	EAI1_SOM
530	■■■■■■□□□■■■■■■■■■■□□■■■■■■■■■■■■■□■■■□■■■■	2	GNB(2)	AFRI_1
531	■■■□■■■■■■■■■■■■■■■■■■■■■□□□□□□■□□□□■■■■■■■	4	CZE(2) GNB(1) USA (1)	H1
532	■■■■■■□□□■■■■■■■■■■■■■■□■■■■■■■■■■■■■■□■■■■	2	GNB(2)	AFRI_1
533	■■■■■■■■■■□□□□□□□□■■■■■■■■■■■■□■□□□□■■■■■■■	2	GNB(2)	H3
534	■■■■■■■■■■■■■■■■■■■■□□□□■■■□□□□□□□□□□□□□□□	4	ESP(1) GNB(3)	U

535	■■■■■■■■■■■■■■■■■■■■■□□□■■■■■■■■□□□□■■■■■■■	29	BRA(17) CMR(1) FIN(1) GEO(1) GNB(2) MYS(1) PRT(1) RUS(1) USA(3) VEN(1)	T1
536	■■■□□□□□□□□□□□□□□□□□■■■■■■■■■■■■■■■■□■■□■■■■	3	GNB(1) USA (2)	AFRI
537	■■■■■■□□□■■■■■■■■■■■■■■■■■■■■■■■■■■■■□□□□■	2	GNB(2)	AFRI
538	■■■■■■■■■■■□□■■■■■■■■■■■■■■■□□□□■□■■■■■□■■■	2	GNB(1) USA (1)	EAI1_SOM
539	□□□□□□□□□□□□□□□□□□□□□□□□□□□□□□□□□□□□■■□□□□□	19	FXX(4) GBR(6) NLD(6) USA (3)	MICROTI
540	□□□□□□□□□□□□□□□□□□□□□□□□□□□□□□□□□□■■□□□□■■■	3	USA (3)	BEIJING
541	□□□□□□□□□□□□□□□□□□□□□□□□□□□□□□□□□□■■■■■□□■■	6	IND(1) USA (5)	BEIJING
542	□□□■■■■■■■■■■□■■■■■■□□□□■■■■■■■■□□□□■■■□■■■	2	USA (2)	LAM4
543	■□■■■■■■■■■■■■■■■■■■□■■■■■■■□■■■□□□■■■■■■■■	2	USA (2)	U
544	■■□□□□□□□□□□□□□□□□□□□□□□□□□■■■■■□□□□■■■□■■■	2	USA (2)	T2
545	■■□■■■■□■■■■□■■■■■■■□□□□■■■■■■■■□□□□■■■■■■■	4	FXX(2) USA (2)	LAM2
546	■■■□□□□□□□□□□■■■■□■■■■■■■■■■□■■■□□□□■■■■■■■	8	HND(3) USA(5)	X3
547	■■■□□□□□□□□□□■■■■□■■■■■■■■■■■■■■□□□□□□□□■■■	2	USA (2)	U
548	■■■□□□□□□□□□■■■■■□■■■□□□□□□□□□□□□□□□□□□■■■■	6	RUS(1) USA(5)	U
549	■■■□□□□□□□□□■■■■■□■■■■□■■■■■■■■■□□□□■■■■■■■	3	USA (3)	X3
550	■■■□□■■■■■■■■■■■■■■□□■■■■■■■□□□□■□■■■■■□■■■	2	USA (2)	EAI1_SOM
551	■■■□■■■■■■■■■■■■■■■■■■■■■■■■■■■■□□□□■■□□□■□	2	USA (2)	T1
552	■■■■□□□□□□□□■■■■■□■■■■■■■■■■■■■■□□□□■■□□□□■	2	USA (2)	X2
553	■■■■□□□□■■■■□■■■■■■■■■■■■■■■■■■■□□□□■■■□■■■	2	USA (2)	T2-T3
554	■■■■□■■■■■■■□□□□□□■■■■■■■■■■■■■■□□□□■■■■■■■	5	USA (5)	T1
555	■■■■□■■■■■■■■■■■■□■■■■■■■■■■■■■■□□□□■□■■■■■	2	USA (2)	X1
556	■■■■■■□■■■■□□□□□■■■■■■■■■□□□■■■■□□□□■■■■■■■	3	USA (3)	T1
557	■■■■■■□■■■■■■■■■■■■■■■■■■□□□□□□■□□□□■■■■■■■	9	USA (9)	H1
558	■■■■■■■■□□■■■■■■■■■■■■■■□□□■■■■■□□□□■■■□■■■	2	USA (2)	S
559	■■■■■■■■□□■■■■■■■■■■■■■■□□□■■■■■□□□□■■■■■■■	4	USA(4)	S
560	■■■■■■■■■□□□□□□□□□□□□□□□□□□□□□□□□□□□□■■■■■■	5	GEO(1) RUS(3) USA (1)	U
561	■■■■■■■■■■□■□□□□□□□□□□□□□■■■■■■■□□□□■■■■■■■	4	RUS(2) USA (2)	T1
562	■■■■■■■■■■■■□■■■■■■■□□□□□□□□□□□□□□□□□□□□□□□	6	MEX(1) MYS(1) USA(4)	U
563	■■■■■■■■■■■■□■■■■■■■■■■□□□□□□□□□□□□□□□□■■■■	5	FXX(1) USA (4)	U
564	■■■■■■■■■■■■□■■■■■■■■■■■□□■□□□□■□■■■■■■■■■	10	NOR(2) THA(1) USA (3) VNM(4)	EAI4_VNM
565	■■■■■■■■■■■■□■■■■■■■■■■■■■■■■■■■□□□□■■□■■■■	4	MEX(1) USA (3)	T3
566	■■■■■■■■■■■■■□□□□□■■■■■■■■■■■■■■□□□□■■■□■■■	6	USA (6)	T2
567	■■■■■■■■■■■■■■□□□□□□□□□□■■■■■■■■□□■■■■■■■■■	5	RUS(3) USA (2)	MANU2
568	■■■■■■■■■■■■■■□■■■■□□■■■■■■■■■■■□□□□■■■■■■■	3	THA(1) USA (2)	H37Rv?
569	■■■■■■■■■■■■■■□■■■■■■■■□□□□□□□□□□□□□□□□□□□□	4	CZE(2) USA (2)	U
570	■■■■■■■■■■■■■■■□■■■■■■■■■■□■■■■■□□□□■■■■■■■	2	USA (2)	T1
571	■■■■■■■■■■■■■■■■□■■■■■■■□□□□□□■□□□□■■■■■■■	2	USA (2)	H1
572	■■■■■■■■■■■■■■■■■□■□□□□□■■■□■■■■□□□□■■□□□□■	4	THA(1) USA (3)	X2
573	■■■■■■■■■■■■■■■■■■■■□■■□□■■■□■■■□□□□■■■■■■■	6	MEX(2) USA (3) VEN(1)	T1
574	■■■■■■■■■■■■■■■■■■■■■■■■■■■■■■■□□□□□□□□■■■■	3	ESP(1) USA (2)	U
575	■■■■■■■■■■■■■■■■■■■■■■■■■■■■■■■■□□□□■□■■■□■	9	IND(1) USA (8)	T1
576	■■■■■■■■■■■■■■■■■□■■■■■■■■■□■■■■□□□□■■■■■■■	19	USA (19)	X1
577	■□□■■■■■■■■■■■□■■■□■□■■■■■■■□□□□■□■■■■■■■■■	5	DNK(5)	EAI5
578	■■□□■■■■■■■■■■■■■■■■□□□□■■■■■■■■□□□□■■■■■■■	16	HTI(3) USA(10) VEN(3)	LAM9
579	■■■■■■■■■■■■□■■■■■■■□□□□■■■■■■□□□□□□□□■■■■■	2	USA (2)	U

580	■■□■■■■■□□□□■■■■■■■■□□□□■■■■■■■■□□□□■■■■■■■	2	NAM(2)	LAM1
581	■■■■■■■■■■■■■■■■■■■■□■■■■■■■□■■■□□□□■■□■■■■	2	NAM(1) USA (1)	T1
582	■■■■■■■■■□□□□□□□■□■■■■■■■■■■■■■■□□□□■■■□■■■	2	FXX(2)	X1
583	■■■■■■■■■■■■□■■■■■■■■■■■■■■■■■■■□□■■■■■■■■■	2	FXX(2)	MANU2
584	■■■■■■■■■■■■■■■■□■■■■■■■■■■■■■■□□□□■■■□■■■	7	FXX(2) NLD(2) SWE(3)	T2
585	□□■■■	5	BRA(2) FXX(2) USA(1)	BEIJING-LIKE
586	□□□□□□□□□□□□□□□□□□□□□□□□■■■■■■□■□□□□■■□□■■■	2	FXX(2)	H3
587	■■■□□□□□□■■■■□□□□□□□□□□□■■■■■■■■■■□□■■□■■■■	8	BGD(3) DNK(1) FXX(1) GBR(1) NLD(1) USA (1)	AFRI
588	■■■□□□□■■■■■■■■■■■■■■■□□■□□□□□□□□□■■■■■■■■■	2	FXX(2)	U
589	■■■■■■■■□□□■■■□□□■□□□□□□□□□□□□□□□□□□□□□■■■■	2	ESP(1) FXX(1)	U
590	■■■■■■■■■■■■■■■■■■■□□□□□□■■■■■■■□□□□■■■□■■□	2	EAFR(1) FXX(1)	T2
591	■■■■■■■■■■■■■■■■■■■■■■□■■■■■□□□□■□■■■■■■■■■	24	AUT(2) BGD(1) FXX(1) ITA(1) MYS(10) NLD(4) SWE(1) THA(2) USA (2)	EAI6_BGD1
592	□□□□□□□□□□□□□□□□□□□□□□□□□□□□□■□□□□□■□□□□□□□	6	CHE(1) FXX(1) GLP(1) SOM(3)	CANETTII
593	□□□■■■■□□□□□□□□□□□□□□□■■■■■■■■■■■■■■■■□□□□□	9	ARG(4) FXX(3) GBR(2)	PIN
594	■■□■□■■■□■□■■■■□■■■■■■■■■■■■■■■■■■■■■■□□□□□	43	ARG(8) BRA(7) GBR(8) IRL(10) PRY(1) SWE(1) USA(3) ZAF(5)	BOVIS1
595	■■□■■■■■□■■■■■■■■■■■■■■■■■■■■■■■■■■■■■□□□□□	5	DNK(1) IRN(2) NLD(1) USA(1)	BOV
596	■□□■□■■■■■■■■■■■■■■■■■■■■■■■□□□■□□□□■■■■■■■	2	IRN(2)	H4
597	■□■■■□■■■■■■■■■■■■■■■■■■■■■■□□□■□□□□■■■■■■■	2	IRN(2)	H4
598	■■■□□□□■■■■■■■■■■■■□□□□□□□□□□□□□□□□□□■■■■■■	3	IRN(3)	CAS
599	■■■□□□□■■■■■■■■■■■■□□□□□□□□□□□□□□□□□■■■■■■■	5	AUT(2) BEL(1) IRN(2)	CAS
600	■■■□□□□■■■■■■■■■■■■□□□□□□□□□□□□□□□■■□□■■■■■	5	IRN(5)	CAS
601	■■■□□□□□□□■■■■■■■■■□□□□□□□□□□□□□□□□□■■■■■■■	2	AUT(1) IRN(1)	CAS
602	■■■■■■■■■■■■■■■■■■■■■■■■□□□□□□□□□□□□■■■■■■■	48	AUT(4) BEL(2) BRA(5) DEU(1) FIN(1) GEO(21) IDN(1) IRN(1) ITA(1) NLD(2) NZL(1) RUS(1) SWE(1) USA(4) USA (1) VNM(1) ZAF(1)	U
603	■■■■■■■■■■■■■■■■■■■■■■■■■■■□■■□■□□□□■■■□■■■	4	USA (2) VEN(2)	H3
604	■■□■■■■■■■■■□■■■■■■■□□□□■■■■■■■■□□□□■■■■□■■	4	ARG(1) BEL(1) GNB(1) VEN(1)	LAM2
605	■■■■■■■■■■■■□■■■■■■■□□□□■■■■■■□□□□□□□□□□■■■	20	USA (1) VEN(19)	U
606	■■■■■■■■■■■■□■■■■■■■□□□□■■■■■■■■□□□□□□□□□□□	4	FXX(1) VEN(3)	U
607	■■■■■■■■■■■■■■■■■■■□□■■□■■■■□□□□□□□□□□□□□□□	2	FXX(2)	U
608	■■■■■■■■□□□■■■■■■■■■■■■■□□□□□□□□□□□□□□□□□□□	7	BEL(2) DEU(2) FXX(1) TUR(1) USA(1)	U
609	■■■■■■■■■■■□□□□□□□■■■■■■■□□□□□□■□□□□■■■■■■■	3	FXX(3)	H1
610	■■■■■■■■■■■■■■□■■■■■■■■■■□□□□□□■□□□□■■■□■■■	3	BEL(1) FXX(2)	H1
611	■■■■■■■■■■■■■■□■■■■■■■■■■■■■□■■■□□□□■■■■■■■	2	FXX(1) GNB(1)	T1
612	■■■■■■■■■■■■■■■■■■■■■■■■■■■■■■■■□□□□■■■■□■■	7	ARG(1) FXX(2) GBR(1) USA (3)	T1
613	■■■■■■■■■□□■■■■■■■■■■■■■■■■■■■■■□□□□■■■■■■■	8	AUT(3) FXX(1) ITA(1) SWE(1) USA(2)	T1
614	■■■■■■■■■■■■■■■■■■■■□□□□■■■■■■■■□□□□■□□□■■■	2	FXX(1) USA (1)	LAM9
615	■■■■■■■■■■■■■■■■■■■■■■■■■■■■■■□■□□□□■■■■■■□	3	AUT(1) FXX(1) USA(1)	H3
616	■□□□□□□□□□□□□□□□□□□□□□□□□□□□□□□□□□■■■■■■■■■	3	USA (1) VNM(2)	U
617	■■■■■■■■■■■■□■■■■■■■■■■■■■□■□□□□■□■■■■■■■■■	2	USA (1) VNM(1)	EAI5
618	■■■■■■■■■■■■□■■■■■■■■■■■■■■■□□□□■□■■■■■■■■■	6	FXX(1) NLD(1) THA(1) USA (1) VNM(2)	EAI5
619	■■■■■■■■■■■■■■■■■■□□□□□□□■□□□□□□□□□□□□□□□■■	3	USA (1) USA (1) VNM(1)	U
620	■■■■■■■■■■■■■■■■■■■■■■□□■□□□□□□■□□□□■■■■■■■	7	AUT(3) BEL(1) FXX(1) USA (1) VNM(1)	H1
621	□□□□□□□□□□□□□□□□□□□□□□□□□□□□□□□□□□■□■■■■■■■	6	ARM(2) IND(1) USA(1) VNM(2)	BEIJING
622	■■■■■■■■■■■□□□□□□■■■■■■■■□□■□□□□■□■■■■■■■■■	5	VNM(5)	EAI4_VNM
623	■■■■■■■■■■■■■■■■■■■■■■□■■■■■■■■■■■■■■■■■■■■	9	DEU(1) GUF(1) MYS(2) VNM(5)	U

624	■□□■■■■■■■■■■■■■□■■■■■■■■■■■□□□□■□■■□□□■■■■	2	USA (2)	EAI3_IND
625	■□□■■■■■■■■■■■■■■■■■■■■■■■■■□□□□■□■□□□□■■■■	4	GBR(1) NLD(1) USA (2)	EAI5
626	■□□■■■■■■■■■■■■■■■■■■■■■■■■■□□□□■□■□■■■■■■■	2	USA (2)	EAI5
627	■■■■□□□□■■■■□■■■■■■■■■■■■■■■■■■■□□□□■■■■■■■	11	FIN(1) IND(1) JPN(8) USA (1)	T3-OSA
628	■■■■■■■■■■■■■■■■■■■■■■■■■■■■■■■■□□□□■■■■■□□	3	AUT(1) NLD(1) USA (1)	T1
629	■□□■■■■■■■■■■■■■■■■■■■■■■■■■□□□□■□□□□□□■■■■	3	GBR(1) IND(1) USA (1)	U
630	■■■■■■□□■■■■■■■■■■■■■□■■■■■■■■■■□□□□■■■■■■■	6	FXX(1) GLP(1) HTI(2) USA (2)	T1
631	■■■■■■■■■■■■■■■■■■■■■■■□■■■■■■□■□□□□■■■■■■■	8	AUT(1) BEL(2) BRA(1) CMR(1) CZE(1) FXX(1) USA (1)	H3
632	□□□□□□□□□□□□□□□□□□□□□□□□□□□□□□□□□□■■■□■■■■■	3	GEO(1) THA(2)	BEIJING
633	■■□■□■■■■■■■■■■■■■■■□□□□■■■■■■■■□□□□■■■□■■■	4	GLP(1) HTI(3)	LAM4
634	□□□□□□□□□□□□□□□□□□□□□□□□■□□□□■■■■■■■■■□□□□□	6	ESP(6)	PINI
635	□□□□□□□□□□□□□□□□□□□□□□□□■■■■□■■■□□□□■■■■■■■	5	BEL(1) BRA(4)	T1
636	□□□□□□□□□□□□□□□□□□□□□□□□■■■■■■■■■■■■□□□□□□□	7	BEL(7)	BOV
637	□□□□□□□□□□□□□□□□□□□□□□□□■■■■■■■■■■■■■■□□□□□	83	BEL(55) FXX(28)	PINI
638	□□□□□□□□■■■■■■■■■■■■□□□□■■■■■■■■□□□□■■■■■■■	2	NLD(2)	LAM9
639	□□□■□□□□□□□□□□□□□□□□□□□□□□□□□□□□□□□□■■□□□□□	3	GBR(3)	MICROTI
640	□□□■■■■□□□□□□□□□□□□□□□□□□■■■□□■■■■■■■□■□□□□□	2	GBR(2)	PIN
641	□□□■■■■□□□□□□□□□□□□□□□■■□□□□□□□□□□□□■■□□□□□	8	GBR(7) USA (1)	MICROTI
642	□□□■■■■□□□□□□□□□□□□□□□■■□■□□□□□□□□□□■■□□□□□	8	BEL(1) DEU(1) FXX(3) GBR(3)	MICROTI
643	□□□■■■■■■■■■■■■■■■□■■■■■■■■□□□□□□□□□□■■■■■■	2	NLD(2)	U
644	□■□□□□□□□□□□□□□□■■□■■■■■■■■□■□□□□■■■■■□□□□□	27	DEU(3) ESP(23) FXX(1)	CAP
645	□■□□□□□□□□□□□□□□■■■■■□□■■■■□■□□□□■■■■■□□□□□	3	ESP(2) NLD(1)	CAP
646	□■□□□□□□□□□□□□□□■■■■■■■■■■■□■□□□□■■■■■□□□□□	14	DEU(4) ESP(9) FXX(1)	CAP
647	□■□□□□□□□□□□□□□□■■■■■■■■■■■□■■■■■■■■■■□□□□□	80	?(1) AUT(8) BEL(1) DEU(50) FXX(12) GBR(1) HND(2) ITA(3) SWE(2)	CAP
648	□■□□□□□□□□■□□□□■■■■■□□■■■■□■□□□□■■■■■□□□□□	2	NLD(2)	CAP
649	□■■■■■■■■■■■■■■■■■■□□□■□□■■■□■■■□□□□■■■■■■■	5	KEN(2) NLD(2) SWE(1)	T1
650	■□□□■■■■□■■■■■■□■■■■□■■■■■■■■■■■■■□□■■□□□□□	10	BEL(2) DEU(1) FXX(6) NLD(1)	BOV
651	■□□■■□■□□□□□■■□□■■■■■■■■■■■■■■■■■■■■■■□□□□□	2	GBR(1) NZL(1)	BOV
652	■□□■■■■■■■□■■■■■■■■■■■■■■■■■□□□□■□■■□□□■■■■	5	DNK(2) GBR(1) NLD(2)	EAI3_IND
653	■□□■■■■■■■■■■■■■■■■■□□□□■□□□□□□■□□□□■■■■■■■	2	NLD(2)	LAM9
654	■□□■■■■■■■■■■■■■■■■■■□■■■■■■□□□□■□■■□□□■■■■	4	IND(1) NLD(2) USA (1)	EAI3_IND
655	■□□■■■■■■■■■■■■■■■■■■■■■■■■■■■□■□□□□■■■■■■■	18	BEL(1) FXX(1) IDN(6) NLD(3) USA (7)	H3
656	■□■■■■■■■■■■■■■■■■□■■■■■■■■■□□□■□□□□■■■■■■■	2	IRN(1) NLD(1)	H4
657	■■□□□□□□□□□□□□□□□□□□□□□■■■□□□■■■■■■■■■□□□□□	2	ARG(2)	BOV
658	■■□□□□□□□□□□□□□□□□□□□□□■■■■■■■■■■■■■■■□□□□□	41	ARG(33) BRA(4) MEX(4)	BOV
659	■■□□□□□■□■■■□■■□■■■■■■■■■■■■■■■■■■■■■■□□□□□	6	BDI(6)	BOVIS1
660	■■□□□□□■□■■■■■■□■■■■□□□□■■■■■■■■■■■■■■□□□□□	4	BDI(4)	BOVIS1
661	■■□□□□□■□■■■■■■□■■■■■■■■■■■■■■■■■■■■■■□□□□□	8	DEU(1) NLD(4) UGA(3)	BOVIS1
662	■■□□□■■□□□□□■■■□■■■■■■■■■■■■■■■■■■■■■■□□□□□	30	BEL(9) FXX(21)	BOV
663	■■□□□■■■□■■■■■■□■■■■□■■■■■■■■■■■■■■■■■□□□□□	14	CAN(1) ESP(11) ITA(1) NLD(1)	BOVIS1
664	■■□□□■■■□■■■■■■□■■■■■■■■■■□□■■■■■■■■■■□□□□□	2	NLD(1) SAU(1)	BOVIS1
665	■■□□□■■■□■■■■■■□■■■■■■■■■■■■■■■■■■■■■■□□□□□	120	?(5) ARG(1) BEL(20) BRA(1) DEU(3) ESP(3) FXX(71) GBR(2) ITA(7) ITAS(1) NLD(2) REU(2) ZAF(2)	BOVIS1
666	■■□■□□□□□□□□□□■□■■■■■■■■■■■□□■■■■■■■■■□□□□□	7	GBR(7)	BOVIS3
667	■■□■□□■□□□□□■■■□■■■■■■■■■■■■■■■■■■■■■■□□□□□	20	ARG(1) ESP(1) GBR(10) IRL(8)	BOV

668	■■□■□■■■□■□□■■■□■■■■■□■■■■■■■■■■■■■■■■□□□□□	11	? (1) ARG(3) GBR(5) URY(1) ZAF(1)	BOVIS1
669	■■□■□■■■□■□■■■■□■■□■■■■■■■■■■■■■■■■■□■□□□□□	5	ARG(5)	BOVIS1
670	■■□■□■■■□■□■■■■□■■□■■■■■■■■■■■■■■■■■■■□□□□□	9	GBR(4) MEX(1) USA(2) ZAF(2)	BOVIS1
671	■■□■■□■□□□□□■□□□■■■■■■■■■■■■■■■■■■■■■■□□□□□	4	GBR(1) IRL(3)	BOV
672	■■□■■□■□□□□□■■□□■■■■■■■■■■■■■■■■■■■■■■□□□□□	21	ARG(4) BRA(3) GBR(6) IRL(3) MEX(4) NZL(1)	BOV
673	■■□■■□■□□□□□■■■□□□□■■■■■■■■■■■■■■■■■■■□□□□□	4	AUS(1) GBR(1) IRL(2)	BOV
674	■■□■■□■□□□□□■■■□□■■■■■■■■■■■■■■■■■■■■■□□□□□	3	ARG(3)	BOV
675	■■□■■□■□□□□□■■■□■■□■■■■■■■■■■■■■■■■■■■□□□□□	6	ARG(1) GBR(5)	BOVIS2
676	■■□■■□■□□□□□■■■□■■■■■■■■■□□□□□□■■■■■■■□□□□□	177	GBR(107) IRL(70)	BOVIS2
677	■■□■■□■□□□□□■■■□■■■■■■■■■■■■■■■□□□□□■■□□□□□	31	GBR(30) IRL(1)	BOVIS2
678	■■□■■□■□□□□□■■■□■■■■■■■■■■■■■■■□■■■■■■□□□□□	16	ARG(2) GBR(14)	BOVIS2
679	■■□■■□■□□□□□■■■□■■■■■■■■■■■■■■■■□□■■■■□□□□□	428	ARG(1) GBR(112) IRL(315)	BOVIS2
680	■■□■■□■□□□□□■■■□■■■■■■■■■■■■■■■■□■■■■■□□□□□	5	ARG(4) IRL(1)	BOVIS2
681	■■□■■□■□□□□□■■■□■■■■■■■■■■■■■■■■■■■□■■□□□□□	11	ARG(1) BRA(3) FXX(1) GBR(6)	BOVIS2
682	■■□■■□■□□□□□■■■□■■■■■■■■■■■■■■■■■■■■□■□□□□□	66	ARG(3) AUS(1) GBR(9) IRL(53)	BOVIS2
683	■■□■■□■□□□□□■■■□■■■■■■■■■■■■■■■■■■■■■■□□□□□	1811	ARG(250) AUS(1) BEL(2) BRA(25) FXX(10) GBR(481) IRL(993) MEX(7) MTQ(1) NLD(1) NZL(20) PRY(2) URY(3) USA (5) ZAF(10)	BOVIS2
684	■■□■■□■■□■■■■■■□■■■■□■■■■■■■■■■■■■■■■■■□□□□□	25	BEL(4) DEU(1) FXX(8) NLD(2) USA(6) ZAF(4)	BOVIS1
685	■■□■■■■□□□□□■■■□■■■■■■■■■■■■■■■■■■■■■■□□□□□	10	ARG(2) DEU(3) FXX(1) GBR(3) IRL(1)	BOV
686	■■□■■■■□□□□■■□■□■■■■■■■■■■■■■■■■■■■■■■□□□□□	2	DEU(2)	BOV
687	■■□■■■■□□■□■■■■□■■■■■■■■■■■■■■■■□■■■■■□□□□□	2	ARG(2)	BOV
688	■■□■■■■■□■□□■■■□■■■■■■■■■■■■■■■■■■■■■■□□□□□	25	GBR(3) IRL(22)	BOVIS1
689	■■□■■■■■□■□■■■■□■■■■■■■■■■■□□□□□■■■■■■□□□□□	12	GBR(2) IRL(10)	BOVIS1
690	■■□■■■■■□■□■■■■□■■■■■■■■■■■■■□□■■■■■■■□□□□□	5	SWE(1) ZAF(4)	BOVIS1
691	■■□■■■■■□■□■■■■□■■■■■■■■■■■■■■■■■■■■■■□□□□□	175	ARG(60) BRA(23) ESP(1) FXX(9) GBR(9) IRL(20) MEX(4) NZL(22) PRY(3) SWE(1) USA (3) ZAF(20)	BOVIS1
692	■■□■■■■■□■■□■■■□■■■■□■■■■■■■■■■■■■■■■■□□□□□	4	NLD(4)	BOVIS1
693	■■□■■■■■□■■■■□□□■■■■□■■■■■■■■■■■■■■■■■□□□□□	2	NLD(2)	BOV
694	■■□■■■■■□■■■■□□□■■■■■■■■■■■■■■■■■■■■■■□□□□□	4	DEU(1) DNK(1) NLD(2)	BOV
695	■■□■■■■■□■■■■■□□■■■■□■■■■■■■■■■■■■■■■■□□□□□	10	BEL(1) BRA(1) DEU(2) MEX(2) NLD(3) USA(1)	BOV
696	■■□■■■■■□■■■■■■□■■■■□■■■■□□□□■■■■■■■■■□□□□□	21	AUT(1) BEL(1) DEU(1) FXX(16) NLD(2)	BOVIS1
697	■■□■■■■■□■■■■■■□■■■■□■■■■■□□□■■■■■■■■■□□□□□	8	DEU(2) NLD(3) SAU(3)	BOVIS1
698	■■□■■■■■□■■■■■■□■■■■□■■■■■■■■■■■■■■■□■□□□□□	55	ARG(13) BRA(23) CRI(1) ESP(5) FXX(5) MEX(4) NLD(3) USA (1)	BOVIS1
699	■■□■■■■■■■■■■■■■■■■■■■■■■■■■■■□■□□□□■□■■■■■	4	NLD(2) SWE(1) USA(1)	H3
700	■■■□□□□□□■■■■□□□□■□□□□□□■■■■■■■■□■□□■■□■■■■	4	NLD(4)	AFRI
701	■■■□□□□□□■■■■□□□□■□□□□□□■■■■■■■■■■□□■■□■■■■	3	NLD(2) SAU(1)	AFRI
702	■■■□□□□□□■■■■■■■□■■■■■□□■■■■□□□□■□■■■■■■■■■	15	CUB(2) GUF(1) MWI(12)	EAI5
703	■■■□□□□■■□□■■■■■■■■□□□□□□□□□□□□□□□□■■■■■■■■	4	NLD(2) SWE(2)	KILI
704	■■■□□□□■■■■■■■■■■■■■■■□□□□□□□□□□□□■■□□■□□■■	2	IRN(1) NLD(1)	CAS1_DELHI
705	■■■□□■■■■□■■■■■■■■■■■■■■■■■■■■■■□□□□■■■■■■■	2	NLD(2)	T1
706	■■■□■■□□□□□■■■■■■■■□■■■■■■■■■■■■□□□□■■■■■■■	2	NLD(2)	T1
707	■■■□■■■■□□■■■■■■■■■■■■■■■■■■■■■■□□□□■■■■■■■	4	ARG(2) IDN(1) NLD(1)	S
708	■■■□■■■■■■■■■■■■■□■■■■■■■■■■■■■■□□□□■■■■■■■	7	AUT(1) DZA(1) NLD(4) RUS(1)	X1
709	■■■□■■■■■■■■■■■■■■■■□□□□■■■■■■■■□□□□■■■□□□□	2	NLD(2)	LAM9
710	■■■□■■■■■■■■■■■■■■■■■□□□□□□□□□□□□□□□□□□■■■	4	NLD(4)	U

711	■■■□■■■■■■■■■■■■■■■■■■■■■■■■□□□□■□■■■■■□■■■	7	DNK(2) FIN(1) GBR(1) NLD(2) USA (1)	EAI1_SOM
712	■■■■□□■■■■■■■■■■■■■■■■■■■■■■■■■■□□□□■■■□■■■	2	NLD(2)	T2
713	■■■■■□□■■■■■■■■■■■■■■■■■■■■■■■■■□□□□■■■■■■■	6	DNK(1) NLD(2) NZL(3)	T1
714	■■■■■■■■■■■■■■■■■■□□□□□□■■□■■■□■□□□□■■■■■■■	4	HTI(4)	H3-LAM8
715	■■■■■■□□□■□□■■■■■■■■■■■■■■■■■■■■■■■■■■□■■■■	3	CMR(1) NLD(2)	AFRI_1
716	■■■■■■□■□■■■■■■■■■■■□□□□□□□□□□□□□□□□■■■□■■■■	2	GBR(2)	AFRI
717	■■■■■■■□□□□■■■■■■■■■■■■■■■■■■■■■□□□□■■■■■■■	3	CUB(1) FXX(1) NLD(1)	T1
718	■■■■■■■■□□□□□□□□□□□□□□□□□□□□□□□□□□□□■■■■■■■	2	CUB(1) IDN(1)	U
719	■■■■■■■■□□□■■■■■■■■□□□□□■■■■■■■■□□□□■■■■■■■	11	ARG(1) NLD(1) USA(2) ZAF(7)	T1
720	■■■■■■■■□□□■■■■■■■■■□□□□■■■■■■■■□□□□□■■■■■■	4	ARG(4)	U
721	■■■■■■■■□□■■■■■■■■■■■■■■■■■■■■■■□□□□□■■■■■■	5	DNK(1) NLD(2) USA (1) ZAF(1)	U
722	■■■■■■■■■■■■□□□□□□□□□□□□■■■■□□□□■□■■■■■□■■■	2	IDN(2)	EAI1_SOM
723	■■■■■■■■■■■■□□□□□□□□□■■■■□□■□□□□■□■■■■■■■■■	2	NLD(2)	EAI4_VNM
724	■■■■■■■■■■■■□□□□□■□□□□□□□■□□□□□□□□□□■■■□■■■	3	MYS(2) NLD(1)	U
725	■■■■■■■■■■■■□■■■■■■■□□□□■■■■■■■■□□□□■■■■■■□	8	ARG(8)	LAM5
726	■■■■■■■■■■■■□■■■■■■■■■□□■■■■□□□□■□■■■■■■■■■	7	AUS(1) NLD(3) SWE(2) USA(1)	EAI5
727	■■■■■■■■■■■■□■■■■■■■■■■■■□□□□□□■□□□□■■■□■■■	5	NLD(1) POL(1) USA (2) VEN(1)	H1
728	■■■■■■■■■■■■□■■■■■■■■■■■■■■■■■■■□□□□■□□□■■■	2	NLD(2)	T3
729	■■□■□■■■■■■■■■■■■■■■□□□□■■■■■■■■□□□□■■■■■■■	4	GLP(1) HTI(3)	LAM1
730	■■■■■■■■■■■■■□■■■■■■■■■■■■■■□□□□■□■■■■■□■■■	2	IND(2)	EAI1_SOM
731	■■■■■■■■■■■■■■□□□■■■□□□□■■■■■■■■□□□□■■■■■■■	5	BEL(1) FXX(1) ITA(1) NLD(2)	LAM9
732	■■■■■■■■■■■■■■□□■■■■■■■■■■■■■■■■□□□□■■■■■■■	9	ARG(4) AUT(1) USA (4)	T1
733	■■■■■■■■■■■■■■■■□■■■■■□□■■■■□□□□■□■■■■■■■■■	5	IND(3) NLD(1) USA (1)	EAI5
734	■■■■■■■■■■■■■■■■■□■■■■■■■■■■□□□□■□□■■■■□■■■	3	AUS(1) IDN(2)	U
735	■■■■■■■■■■■■■■■■■□■■■■■■■■■■□□□□■□■■■■■□■■■	3	DNK(1) NLD(2)	EAI1_SOM
736	■■■■■■■■■■■■■■■■■■■□■■■■■■■■■■■■□□□□■■■□■■■	12	NLD(12)	T2
737	■■■■■■■■■■■■■■■■■■■■□□□□■■■■■■■■□□□□■□■■■■■	7	FXX(1) ITA(3) MAR(1) VEN(2)	LAM9
738	■■■■■■■■■■■■■■■■■■■■□□□□■■■■■■■■□□□□■■■■■□□	4	ARG(3) LVA(1)	LAM9
739	■■■■■■■■■■■■■■■■■■■■■□□□■■■■□□□□■□■■■■■■■■■	3	NLD(3)	EAI5
740	■■■■■■■■■■■■■■■■■■■■■■□□■■■■■■□■□□□□■■■■■■■	6	AUT(1) DEU(1) GLP(1) NLD(2) PER(1)	H3
741	■■■■■■■■■■■■■■■■■■■■■■□■■■■■■■□■□□□□■■■■■■■	6	AUT(2) NLD(2) POL(1) USA (1)	H3
742	■■■■■■■■■■■■■■■■■■■■■■■■□□□□□□□■□□□□■■■■■■■	18	AUT(2) IDN(2) MYS(3) THA(1) USA (8) VNM(2)	H3
743	■■■■■■■■■■■■■■■■■■■■■■■■■■□□□□□□□□□□□□□■■■■	4	CZE(1) MYS(2) USA(1)	U
744	■■■■■■■■■■■■■■■■■■■■■■■■■■■■□□□□■□□■■■■□■■■	2	NLD(2)	U
745	■■■■■■■■■■■■■■■■■■■■■■■■■■■■□□□□■□■■□□■□■■■	27	IND(1) MYS(26)	EAI1_SOM
746	■■■■■■■■■■■■■■■■■■■■■■■■■■■■□■□■□□□□■■■■■■■	11	BEL(2) FXX(1) IDN(1) NLD(1) POL(1) USA (5)	H3
747	■■■■■■■■■■■■■■■■■■■■■■■■■■■■■■□■□□□□■□□■■■■	7	BRA(3) NLD(4)	H3
748	■■■■■■■■■■■■■■■■■■■■■■■■■■■■■■□■□□□□■■■■■□□	2	NLD(2)	H3
749	■■■■■■■■■■■■■■■■■■■■□■■■■■■■■■■■■■■■■■■■■■■	2	IDN(1) JPN(1)	U
750	□□□□■■■■■■■□■■■■■■■□■□□□□□□□□□□■□□□□■■■■■■■	18	ARG(3) AUT(8) EAFR(1) USA (6)	H3
751	□□□■■■■■■■■■■■■■■■■■■■■■■■■■■■■■□□□□■■■■■■■	9	BEL(1) FXX(1) MAR(1) NLD(1) SAU(1) SWE(1) TUR(1) USA (2)	T1
752	■□□■□□□□□□□□□□■□■■■■■■■■■■■■■■■■■■■■■■□□□□□	2	GBR(1) USA (1)	BOV
753	■□□■■■■■■■■■■■■■■■■■□□□□■■■■■■■■□□□□■■■■■■■	12	BRA(1) GUF(1) MWI(2) NLD(1) USA(2) ZAF(1) ZWE(4)	LAM9
754	■□■□□□□■■■■■■■■■■■■■■■□□□□□□□□□□□□■■■■■■■■■	2	GBR(1) IRN(1)	CAS1_DELHI
755	■□■■■■■■■■■■■■■■■■■■□■■■■■■■■■□■□□□□■■■■■■■	2	ARG(1) USA(1)	H3

756	■■□■■■□■■■■■■■■■■■■□□■■■■■■■□□□□■□■■■■■■■■■	2	USA (1) VNM(1)	EAI2_MANILLA
757	■■□■■■■■□■□■■■■□■■■■■■■■■■■■■□■■■■■■■■□□□□□	2	GBR(1) NLD(1)	BOVIS1
758	■■□■■■■■■■■■■■■■■■■□□■■■■■■■□□□□■□■■■■■□□□□	2	PHL(1) USA (1)	EAI2_MANILLA
759	□□□■■■■■■■■□□■■■■■■■■■■■■■■■■■■■■□□□□■■■■■■■	2	TUR(2)	T1
760	■■■■■■■□□□□□□■■■■■□□□□□□□□□□□□□■□□□□■■■□□□□	7	ARG(1) AUT(1) DZA(3) FXX(1) ITA(1)	H3
761	■■■■■■■□□□□□■■■■■■□□□□□□■■■■■■■■■■■■□□□■■■■	3	DEU(1) USA(2)	AFRI_3
762	■■■■■■■□□■■■■■■■■■■■■■■■■■■■□□□■□□□□■■■□■■■	2	NLD(1) RUS(1)	H4
763	■■■■■■■■■■■■□□□□□□■■■■■■■■■■□□□□■□■■■■■□□□□	5	DNK(1) MYS(1) NLD(1) USA (2)	EAI5
764	■■■■■■■■■■■■■□■■■■■■■■■■■■■■■■□■□□□□■■■■■■■	4	BEL(1) NLD(1) SEN(1) VNM(1)	H3
765	■■■■■■■■■■■■■■□□□□□□□■■■■■■■■■■■□□□□■■■■■■■	5	DEU(1) NLD(1) USA (3)	T1
766	■■■■■■■■■■■■■■□□□■□□□□□□■■■■■■■■□□□□■■■■■■■	11	EST(5) FIN(2) FXX(1) NLD(1) PRT(1) ZAF(1)	T1
767	■■■■■■■■■■■■■■■□■■■■■■■■■■■■□□□□■□■■■■■□□□□	3	GBR(1) ITA(1) NLD(1)	EAI5
768	■■■■■■■■■■■■■■■■■■■□□□□□■■■■■■□■□□□□■■■■■■■	6	AUT(1) DEU(2) NLD(1) USA (2)	H3
769	■■■■■■■■■■■■■■■■■■■□□■■■■□□□□□□■□□□□■■■■■■■	2	AUT(1) CUB(1)	H1
770	■■■■■■■■■■■□■■■■■■■■□□□□■■■■■■■■□□□□■■■■■■■	4	ARG(1) ESP(1) FXX(1) ITAS(1)	LAM9
771	■■■■■■■■■■■■■■■■■■■■■□□■■■■■■■■■□□□□■■■■■■■	3	ARG(1) AUT(1) NLD(1)	T1
772	■■■■■■■■■■■■■■■■■■■■■■□□□■■■□□■■□□□□■■■■■■■	8	FXX(2) ITA(2) NLD(1) SWE(1) USA(2)	LAM10_CAM
773	■■■■■■■■■■■■■■■■■■■■■■■□□□□□□□□□□□□□□□□□■■■	5	IDN(1) USA (4)	U
774	■■■■■■■■■■■■■■■■■■■■■■■■□■■■■■■■□□□□■■■■■■■	5	CMR(2) IDN(1) RUS(1) USA (1)	T1
775	■■■■■■■■■■■■■■■■■■■■■■■■■■■□□□□□□□□□□■■■■■■	20	AUT(4) CZE(8) HUN(1) NLD(1) SWE(1) USA (5)	U
776	■■■■■■■■■■■■■■■■■■■■■■■■■■■■□□□□■□□□■■■■■■■	4	IDN(1) MYS(2) USA (1)	CAS
777	■■■■■■■■■■■■■■■■■■■■■■■■■■■■□□□■□□□□■■■■■■■	24	ARM(1) BRA(1) FIN(1) GEO(1) KAZ(1) NLD(1) RUS(4) SAU(8) SWE(1) USA(5)	H4
778	■■□■■■■■□■□■■■■□■■■■■■■■■■■□□□■■■■■■■■□□□□□	5	ARG(3) MEX(2)	BOVIS1
779	■■■■■■■■■■■■■■■■■■■■■□□■■■■■■■□■□□□□■■■■■■■	2	FXX(2)	H3
780	■■■■■■■■■■■■■■■■■■■■■■■■■■■■■□□□□□□□■■■■■■■	6	FXX(5) ZWE(1)	U
781	□□□□□□□□□□□□□□□□□□□□□□□□■□□□□□□■□□□□■■■□■■■	6	ARG(1) AUT(1) FXX(1) ITA(1) USA(2)	H2
782	■□□□□■■■■■■■■■■■■■■■□□□□■■■■■■■■□□□□■■■■■■■	2	FXX(1) USA (1)	LAM9
783	■□□■■■■■■■■■■■■■■■■■■■■■■■■■■■■■□□□□■■■□■■■	6	FXX(1) MYS(3) USA (2)	T2
784	■■■■■■■■□□■■■■■■■■■■■■■■■■■■■■■■□□□□■■■□■■■	23	BRA(2) CAN(14) CUB(1) FXX(1) USA (3) ZAF(2)	T2-S
785	■■■■■■■■■■■■■■■■■■■□■■□■■□□■■■■■□□□□■■■■■■■	2	FXX(1) NLD(1)	T5_MAD2
786	■■■■■■■■■■■■■■■■■■■■■■■□□□□□□□□□□□□□□□□□□□□	5	AUT(1) CMR(1) FXX(1) NLD(1) SWE(1)	U
787	■■■■■■■■■■■■■■■■■■■■■■■■■■■■■■■■□□□□□□□■■■■	4	AUT(1) FXX(1) MEX(1) USA(1)	U
788	■□■■■■■■■■■■■■■■■■□□□□□□□■□□□□■■□□□□■■■■■■■	3	NLD(1) SWE(1) USA(1)	T1
789	■■□■■■■■□□■■■■■■■■■■■■■■■■■■■■■■□□□□■■■■■■■	7	ARG(1) AUT(2) ITAS(1) SWE(1) USA(1) ZAF(1)	S
790	■■■■■■■■□□■■■■■■■■■■■■■■■■■■■■■□□□□□■■■■■■■	4	AUS(1) SWE(1) USA(1) ZAF(1)	U
791	■■■■■■■■■■■■■■■■■■■■■■■□□□□□□□□■□□□□■■■■■■■	4	CUB(1) CZE(1) SWE(1) USA(1)	H3
792	■■■■■□□■■■■■■■■■■■■■■■■■■■■■■□□□□■□■■■■■■■■■	2	FXX(1) USA (1)	EAI5
793	■□■■■■■■■■■■□■■■■□■■■■■■■■■■■■■■□□□□■■□□□□■	2	USA (2)	X2
794	■■■□□□□■■■■■■□■■■■■■■■□□□□□□□□□□□□■■■■■■■■■	3	GBR(1) PAK(1) USA(1)	CAS1_DELHI
795	□□□□□□□□□□□□□□□□□□□□□□□□■■□■■■■■□□□□■■■■■■■	4	EGY(3) MYS(1)	T1
796	□□□□□□□□□□□□□□□□□□□□□□□□□□□□□□□□□□□■■■■■■■■	7	BGD(2) GEO(3) USA (1) VNM(1)	BEIJING-LIKE
797	■■□■■■■■□■■■■■■□■■■■■■■■■■■■■■■■■■■■□■□□□□□	5	FXX(3) USA (2)	BOVIS1
798	■□□□■■■■■■■■■■■■■■■■■■■■■■■■■■■■□□□□■■■■■■■	2	FXX(1) NLD(1)	T1
799	■■■■■■□□□■■■■■□■■■■■□■■■■■■■■■■■■■■■■■□■■■■	2	SEN(2)	AFRI_1
800	■■■■■■■□□□□□□□□□□□□■■■■■□□□□□□□■□□□□■■■■■■■	3	USA (2) VNM(1)	H3

801	■■■■■■■□□■■■■■■■■■■■■■■■■■■■■■■■□□□□■■■■■■■	3	DEU(1) USA (1) ZWE(1)	T1
802	■■■■■■■■■■■□□□□□■■□□□□□□□□□□□□□□□□□□□□□□□■■	2	VNM(2)	ZERO
803	■■■■■■■■■■■■■□□□□□□□□□□□■■■■■■■■□□□□■■■■■■■	24	ARM(2) BEL(1) CHN(1) EST(2) GEO(5) LVA(1) POL(1) RUS(7) SWE(1) USA (1) VNM(2)	T1
804	■□□■■■■■■■■■■■■■■■■■■■■■■■■■■■■■□□□□■■■■■■■	11	AUT(1) DNK(1) IND(1) ITA(1) SWE(1) USA(1) USA (2) ZWE(3)	T1
805	■■■□■■■■■■□□□□■■■■■■□□□□■■□□□□■■□□□□■■■□■■■	2	ZWE(2)	LAM11_ZWE
806	■■■■□■■■■■■■■■■■■■■■■■■■■■■■□□□□■□■■■■■□■■■	8	MOZ(2) NOR(2) USA(2) ZWE(2)	EAI1_SOM
807	■■■■■■□□□□□□■■■■■■■■□□□□■■□□□□■■□□□□■■■□■■■	3	ZWE(3)	LAM11_ZWE
808	■■■■■■■■■■■■■□□■■■■■□□□□■■□□□□■■□□□□■■■■■■■	2	ZWE(2)	LAM11_ZWE
809	■■■■■■■■■■■■■■■■□■■■□□□□■■□□□□■■□□□□■■■■■■■	3	ZWE(3)	LAM11_ZWE
810	■■■■■■■■■■■■■■■■■■■■□□□□□■□□□□■■□□□□■■■■■■■	2	ZWE(2)	T1
811	■■■■■■■■■■■■■■■■■■■■□□□□■□□□□□■■□□□□■■■□■■■	12	MOZ(1) USA(1) ZAF(2) ZWE(8)	LAM4
812	■■■■■■■■■■■■■■■■■■■■□□□□■■□□□□■■□□□□■□■■■■■	3	ZWE(3)	LAM11_ZWE
813	■■■■■■■■■■■■■■■■■■■■□□□□■■□□□□■■□□□□■■□□■■■	7	USA(1) ZWE(6)	LAM11_ZWE
814	■■■■■■■■■■■■■■■■■■■■□□□□■■□□□□■■□□□□■■□■■■■	4	ZWE(4)	LAM11_ZWE
815	■■■■■■■■■■■■■■■■■■■■□□□□■■□□□□■■□□□□■■■□■■■	14	BEL(1) MWI(2) USA(2) ZWE(9)	LAM11_ZWE
816	□□□■■■■■■■■■■■■■■■■■□□□□■■□□□□■■□□□□■■■■■■■	8	MDG(1) MWI(5) USA(1) ZWE(1)	LAM11_ZWE
817	■■■■■■■■■■■■■■■■■■■■■■■■■■■■□□□■□□□□■■■□■■■	4	ETH(1) USA (2) ZWE(1)	H4
818	□■□□□□□□□□□□□□□□■■■■■■■□□□□□□□□□□□□□□□□□□□□	8	AUT(1) DEU(4) FXX(2) ITA(1)	CAP
819	■■□■■■■■□■■■■■□□■■■■□■■■■■■■■■■■■■■■■□□□□□□	3	DEU(3)	BOV
820	■■□■■■■■□■■■■■□□■■■■■■■■■■■■■■■■■■■■■■□□□□□	47	BRA(5) DEU(6) DNK(29) FXX(3) MEX(1) NOR(1) USA (2)	BOV
821	■■□■■■■□□□□□□□□□□□□□□□□□□□□□□□□□□□□□□□■■■■■	6	BRA(1) USA(4) VEN(1)	U
822	■■■■□■■■■■■■■■■■■■■■□□□□■■■■■■■■□□□□■■■■■■■	4	BRA(2) GLP(1) USA (1)	LAM9
823	■■■■■■■■□□□□□□□□□□□□□□□□■■■■■■■■□□□□■■■■■■■	10	BRA(2) DEU(1) FIN(1) ITA(1) ITAS(1) USA(4)	T1
824	■■■■■■■■■■■■■■■■■■■□□■□■■■■■■■■■□□□□■■■■■■■	3	BRA(1) ESP(1) PER(1)	T5
825	■■■■■■■■■■■■■■■■■■■■□□□□□□□□□□□□□□□□□□■■■■■	4	AUT(2) BRA(1) GBR(1)	U
826	■■□■■■■■■■■■□■■■■■□■□□□□■■■■■■■■□□□□■■■■■■■	3	BRA(2) VEN(1)	LAM2
827	■■■■■■■■□□■■□■■■■■■■■■■■□□□■■■■■□□□□■■■■■■■	4	ARG(1) BRA(2) ITA(1)	S
828	□■■■■■■■■■■■■■■■■■■■□□□□■■■■■■■■□□□□■■■□■■■	6	BRA(5) GNB(1)	LAM4
829	■■□■□□□□□□□□□□□□■■■■■■■■■■■■■■■■■■■■■■□□□□□	8	ARG(2) GBR(1) GLP(1) MEX(4)	BOV
830	□□□□□□□□□□□□□□□□□□□□□□□□■■■■■■□■□□□□■■■□■■■	6	CMR(2) FXX(1) GLP(1) USA(2)	H3
831	■■■■■■■■□□■■■■□■■■■■■■■■■■■■■■■■□□□□■■■■■■■	6	FXX(1) GLP(1) ITAS(1) MYS(1) PRT(1) ZAF(1)	S
832	■■■■■■■■■■■■□■□■■■■■■■■■■■■■■■■■□□□□■■■□■■■	3	FXX(3)	T2-T3
833	■■■■□■■■■■■■■■■■■■■■■■■■■■■■■■■■□□□□■■■□□□□	2	FXX(2)	T1
834	■■■■■■■■■■■■■■□■■■■■■■■■■■■■■■■□□□□□■■■■■■■	4	FXX(2) IDN(1) PRT(1)	U
835	■■■■■■■■■■■■■■□■■■■■■■■■■■■■■■□■□□□□■■□■■■■	2	FXX(2)	H3
836	■■■■■■■■■■■■■■■■■■■■■■■■■■■■■■□□□□□□□□■□■■■	3	CMR(1) ITA(1) USA(1)	U
837	■■■■■■■■■■■■■■■■■■■■■■■■■■■□□□□■□□□□■■■□■■■	2	CMR(1) FXX(1)	H3
838	■■■■■■■■■■■■■■■■■■■■■■□□□■■■■■■■□□□□■■■■□■■	6	BEL(1) CMR(4) SDN(1)	LAM10_CAM
839	■■■■■■■■■■■■■■■■■■■■■■□□□■■■■■□□□□□□□□■■■■■	3	CMR(2) FIN(1)	U
840	■■■■■■■■■■■■■■■■□■■■■■■■■■■■■■□■□□□□■■■■■■■	3	CMR(2) SWE(1)	H3
841	■■■■■■■■■■■■■■□■■■■■□■□□□■■■■■■■□□□□■■■■■■■	3	CMR(3)	LAM10_CAM
842	■■■■■■■■■■■■■■□■■□■■■■■■□□□□□□□□□□□□□□□□□□□	6	CMR(5) ITA(1)	U
843	■■■■■■■■■□□■■■■■■■■■■■■■■■■■■■□■□□□□■■■■■■■	4	AUT(1) CMR(1) MEX(1) POL(1)	H3
844	■■■■■□□□■■■■■■■■■■■■■■□□□■■■■■■■□□□□■■■□■■■	2	CMR(2)	LAM10_CAM
845	■■■■■□□□■■■■■■□■■■■■■■□□□■■■■■■■□□□□■■■□■■■	2	CMR(2)	LAM10_CAM

846	■■■■■□□□□■■■■■■■■■■■■■■■■■■■■■■■□□□□■■■□■■■	3	CMR(2) USA(1)	T2
847	■■■■■□□□□□□□□□□□□■■■■■□□□■■■■■■■□□□□■■■■■■■	4	CMR(4)	LAM10_CAM
848	■■■□■■■■■■■■■■■■■■■■■■■■■■■■■■■■□□□□■■■□■■■	10	CMR(4) DEU(1) FIN(1) GBR(1) LBY(1) USA(2)	T2
849	■■□□■■■■■■■■■■■■■■■■■■■■■■■■■■□■□□□□■■■■■■■	3	CMR(1) DEU(1) SEN(1)	H3
850	■□□□■■■■■■■■■■■■■■■■■■□□□■■■■■■■□□□□■■■■■■■	2	CMR(2)	LAM10_CAM
851	■□□□□□□■■■■■■■■■■■■■■□□□□■□□□□□□□□□□□■■■■■■	2	CMR(2)	U
852	■□□□□□□□□□□□□□□□■■■■■■□□□■■■■■■■□□□□■■■■■■■	13	BEL(1) CMR(12)	LAM10_CAM
853	□■■■■■■■■■■■■■■■■■■■■■■■■■■■■■■■□□□□■■■□■■■	19	AUT(3) BEL(1) CMR(13) DEU(1) USA(1)	T2
854	□□■■■■■■■■■■■■■■■■■□□□□□□■■■■■■■□□□□■■■■■■■	2	CIV(1) CMR(1)	T1
855	□□□□■■■■■■■■■■□■■■■■■■■■■■■■■■■□■□□□□■■■□■■■	2	CMR(2)	H3
856	■■■■■■■□□□□□■■■■■■■■■■■■□□□□□□□□□■□■□□□■■■■	4	CMR(4)	AFRI_3
857	■■■■■■■□□□□□■■□■■■■■■■■■□□□□□□□□□■■■□□□■■■■	2	CMR(2)	AFRI_3
858	■■■■■■■□□□□□■■□■■■■■■■■■□□□□□□□□□■□■□□□■■■■	3	CMR(3)	AFRI_3
859	■■■■■■□□□■■■■■■■■■■■■■■■■■■■■■■□□■■■□□□■■■■	3	CMR(2) USA(1)	AFRI
860	■□■■■■■□□□□□■■□■■■■■□□□□■■■■■■■■■■■■□□□■■■■	2	CIV(1) CMR(1)	AFRI_2
861	■□■■■■■□□□□□■■□■■■■■□□□□■■□■■■■■□■■■□□□■■■■	2	CMR(2)	AFRI_2
862	■■■■■■■■■■■■■■□■■■■■■■■■■■■■□■■■■□□□□■■■□■■■	2	CMR(2)	T2
863	□□□□□□□□□□□□□□□□□□□□□□□□■■■■□□□□□□□□□□□□□□□	4	BRA(3) USA(1)	U
864	■■■□□□□■■■■■■□□□□□□□□□□□□□□□□□□□□□□■■■■■□□	9	IND(1) NLD(1) SUR(1) USA(6)	CAS
865	■□□■■■■■■■■■■■■■■■■■□■■■■■■■□□□□■□■■□□□■■■■	3	USA(3)	EAI3_IND
866	■□■■■■■■■■■■■■■■■■■■□□□□■■■■■■■■□□□□■■■■■■■	18	BRA(1) FXX(2) GNB(1) ITA(9) USA (4) VEN(1)	LAM9
867	■■■■■■■■■■■■□■■■■■■■■□□□□■■■■□■■■□□□□■■■□■■■	5	USA (5)	LAM5-LAM6
868	■■■■■■■■■■■□■■■■■□■■■■■■■■■■■■■■□□□□■■■■■■■	3	USA (3)	X1
869	■■■■■■■■■■■■■■■■□□□□□■□□■■■■□■■■□□□□■■■■■■■	3	USA (3)	T1
870	■■■■■■■■■■■■■■□■■■■■■■■■■■□■■■■■□□□□■■■□■■■	2	USA (2)	T2
871	■■■□■■■■■■■■■■■■■■■■■■■■■■■■■■□■□□□□■■■■■■■	12	ARG(2) ARM(1) FIN(1) ITA(1) USA (7)	H3
872	■■■■■■□■■■■■■■■■■■■■■□□■□□■■■■■■■□□□□■■■■■■■	3	NLD(1) USA (2)	T1
873	■■■□□□□■■■□□□□□□□□□□□□□□□□□□□□□□□□□□□□□□□	3	NLD(1) USA (2)	CAS
874	■■■■■■■■□□■■■■■■■■■■■■■■■■■■■■■■□□□□■□□□■■■	2	USA (2)	S
875	■■■■■■■■■■■■□□■■■■■■■■■■■■■■■■■■□□□□■■■□■■■	4	FIN(1) USA (3)	T2
876	■■■□□□□■■■■■■■■■■■■■■■□□□□□□□□□□□□□□■■■□■■■	5	IND(2) NLD(2) USA (1)	CAS
877	■■■■■■□□□□□□□□□□□□■■■■■■□□□□□□□□□□□□□□□□□□□	4	FIN(1) NLD(1) USA (2)	U
878	■■■■■■■■■■■■■■■■■□■■■■■■■■■■■■■■□□□□■□■■■■■	8	AUT(2) BEL(1) EGY(1) GBR(1) LBY(1) TUR(1) USA (1)	X1
879	■■■■■■■■■■■■■■□■■■■■■■■■■■■■■■■■□□□□■■□■■■■	4	ARG(1) FXX(1) RUS(1) USA (1)	T1
880	■■■■■■■■■■■■■■■■■■□□□□□□□□□■■■■■□□□□■■■■■■■	7	MEX(2) USA(3) VEN(1) ZAF(1)	T1
881	■■■■■■■■□□■■■■■■■■■■■■■■□□□□□□□□□□□□■■■□■■■	4	USA(3) VEN(1)	U
882	■□■■■■■■■■■■■■■■■■■■■■□■■■■■□□□□■□■■■■■■■■■	17	BGD(12) GEO(1) ITA(1) MYS(1) USA (2)	EAI6_BGD1
883	■■■■■■■■■■■■■■■■■■■■■■□■■□□□□□□■□□□□■■■■■■■	5	ESP(1) ITA(1) USA (3)	H1
884	□■■■■■■■□□■■■■■■■■■■■■■■■■■■■■■■□□□□■■■■■■■	7	ESP(1) ITA(1) USA(4) ZAF(1)	S
885	■■■■■■■■■■■■□■■■■■■■■□□□□■■■■□□■■□□□□■■■■■■■	2	USA (2)	LAM5
886	■■■■■■■■■■■■■■■■■□■■■■■■■■□■■■■■□□□□■■□□□□■	2	USA (2)	X2
887	■□■■□■■■■■■■■■□■■■■■■■■■■■■■■■□■□□□□■■■■■■■	2	USA (2)	H3
888	■■■■■■■■■■■■■■■■■■■■■■■■■■■■■■■■□□□□■■□□■■■	5	ESP(1) ITA(1) POL(1) USA (2)	T1
889	■■■■□■■■■■■■■■■■■■■■■■□■■■■■□□□□■□■■■■■■■■■	3	IND(1) USA (2)	EAI6_BGD1
890	■■■■□□□□■■■■■■■■□□□□□■□□■■■■□■■■□□□□■■■■■■■	7	USA (7)	T1

891	■■■■■■■■■■■■■■■■■■■■□□□□■■■■■□■■□□□□■■■■■■■	12	ESP(1) POL(7) RUS(1) USA (3)	LAM9
892	■■■■■■■■■■■■■■■■■■■□□■■■■■■■□□□□■□■■■■■■■■■	3	USA(3)	EAI5
893	■■■■■■■■■■■■■■■■■■□□□□□■■■■■■■■■□□□□■■■■■■■	2	SEN(1) USA (1)	T1
894	■■□■■■■■■■■■■■■■■■■□□■■■■■■■□□□□■□■■■■■□■■■	9	MYS(1) PHL(4) USA (4)	EAI1_SOM-EAI2
895	■■□■■■■■■■■■■■■■■■■□□■■■■■■□□□□□■□■■■■■■■■■	4	PHL(2) USA (2)	MANU1
896	■■■■■■■■■■■■□■■■□□□□□■■■■■■■□■■■□□□□■■■■■■■	3	CUB(1) USA (2	T3
897	■■□■■■■■□□□□□□□□■■■□□■■■■■■■□□□□■□■■■■■■■■■	4	AUS(1) MYS(1) USA (2)	EAI2_MANILLA
898	■■□■■■■■■■■■■■■■■■■■■■■■■□□□□□□■□□□□■■■■■■■	3	NLD(1) USA (2)	H1
899	■■□■■■□□□□□□□□□□□□■■■■■■■■■■■■■■□□□□■■■■■■■	2	USA (2)	T1_RUS2
900	■■□■■■■■■■■■■■■■■■■■□□□□■■■■■■■□□□□□□□□□□■■	2	USA (2)	U
901	■■■□□□□□□□□□□■■■■□■■■■■■■■■■■■■□□□□□□□□□□■■	3	USA (3)	U
902	■■■■■■■■□□□■□■■■■■■■□□□□■■■■■■■■□□□□■■□□■■■	3	USA (3)	LAM3-LAM5
903	■■■■■■■■■■■■■■■□■□■■■■■■■■■■■■■■□□□□■■□□□□■	3	USA(3)	X2
904	■■■■■■■□■■■■■■■■□■■■■■□■■■■■■■■■□□□□■■■■■■■	6	USA (6)	T5
905	■■□■■■■■■■■■□■■■■■■■□□□□■■■■■■□□□□□□■■■■■■■	15	ARG(1) USA (5) VEN(9)	U
906	■■■■■■■■■■■■■■■■□□□□■■■■■■■■■■□■□□□□■■■■■■■	4	USA (4)	H3
907	■■■■■■■■■■■■■■■■■■■■□■■■■■■■■■■■□□□□■■■■■■□	2	USA (2)	T1
908	■■□■■■■■■■■■□■■■■■■■□□□□■■■■■■■■□□□□■■■□□□□	7	USA (7)	LAM2
909	■■■■□□□□□■■■■■■■■■■■□■■■■■□□□□□□□□□□□□□□□■■	14	CAN(9) USA (5)	U
910	■■■□□□□□□□□□□□□□□□□□□□□□■■■□□■■■■■■■■■■■■■■	5	ETH(2) USA (3)	U
911	■■■■■■■■■■■■■■■■■■■■□□□□□□□□□□■■□□□□■■■■■■■	20	ARG(1) USA (19)	T1
912	■■□■■■■■■■■■■■■■■■■■■■■■■■■■□□□□■□■■□■■■■■■	5	NOR(1) USA(4)	EAI5
913	■■■■■■■■■■■■■□□□■■■■■■■■■■■■■■■■□□□□■■■■■■■	10	AUT(2) CHN(2) FIN(1) MWI(1) SWE(1) USA (2) VEN(1)	T1
914	■■■■■■■■□□■■■■■■■■■■■■■■■■■■■■□■□□□□■■■■■■■	35	CAN(22) FIN(7) HTI(1) ITA(2) USA (2) VEN(1)	H3-S
915	■■■■■□□□■■■■■■■■■■■■■■■■■■■■■■□■□□□□■■■□■■■	2	FIN(2)	H3
916	■■■■■■■■■■■■■■■■■■■■□□□□■■■■■■■■□□□□■□□□□■■	4	FIN(2) USA(2)	LAM9
917	■■■■■■■■■■□■■■■■■■■■■■■■■■■■■■■■□□□□■■■■■■■	7	DNK(1) FIN(2) LVA(1) USA (3)	T1
918	■■■■■■□□□□□□□□□□□□■■□□□□■■■■■■■■□□□□■■■■■■■	8	EST(4) FIN(1) ITA(1) SWE(2)	T1_RUS2-LAM9
919	■■■■■■■■■■■■■■■■■■■■■■■■■□□□□□□■□□□□■■■■■□□	5	CZE(1) FIN(1) POL(2) USA (1)	H1
920	■■■■■■■■■■■□□■■□■■■■■■■■■■■■□□□■□□□□■■■■■■■	2	FIN(2)	H4
921	■□□□■■■■■■■■□■■■■■■■■■■■■■■■□□□■□□□□■■■■■■■	5	FIN(5)	H4
922	■■■■■■■□□■■■■□□■■■□■■■■■■■■■□□□■□□□□■■■■■■■	2	FIN(2)	H4
923	■■□■■■■■□□■■■■■■■■■■■■■■■■■■■■□■□□□□■■■■■■■	3	FIN(3)	H3-S
924	■■■■■■■■■■■□□□□□□□□□□□□□■■■■□□□□■□■■□■■■■■■	9	COM(2) FIN(1) GUF(3) MUS(1) NLD(1) SWE(1)	EAI5
925	■■■■■■■■■■■■□□■■■■■■■■■■■■■■■■□■□□□□■■■■■■■	5	AUT(2) FIN(3)	H3
926	■■■■■■□■■■■■■■■■■■■■■■■■■■■■■■■■□□□□■■■■■■■	8	ARM(3) FIN(1) GUF(1) ITA(1) USA(1) VEN(1)	T1
927	■□□■□□□□□□□□■■□□□□□□□□□□□□□□□□□□□□□□■■■■■■■	2	FIN(2)	U
928	■■■■■■■■■■■■■■■■■■■■■■□■□□□□□□■■□□□□■■■■■■■	10	FIN(8) USA (2)	T5
929	■■■■■■■■■■■■□■■■■■■■■■■■■■■■□□■□□□□■■■■■■■	2	FIN(1) GBR(1)	H3
930	■■□■■■■■■■■■■■■■■■■□□□□□■□□■■■■■□□□□■■■■■■■	6	BEL(2) FIN(2) NLD(1) TUR(1)	LAM7_TUR
931	■■■■■■■□□□□□□□□□□□□□□□□□■■■■■■■■□□□□■■■■■■■	2	FIN(1) NLD(1)	T1
932	■■■■■■■■■■■■■■■■■■■■□■■□□■■■■■■■□□□□■■■□■■■	4	BEL(1) FIN(1) RUS(1) USA (1)	T2
933	■■■■■■■■■■■■■■■■■■■■□□□□■■■■■■■■□□□□□□□■■■■	5	FIN(1) RUS(2) USA (2)	U
934	■■■■■■■■■■■■■■■■■■■■■■■■■■■■□□□□■□■■□□□■■■■	6	FIN(1) SWE(1) THA(2) USA(2)	EAI5
935	■■■■■■■□■■■■■■■■■■■■■■■■■■■■■■□■□□□□■■■■■■■	5	FIN(2) USA(3)	H3

936	■■■■□□□□□■■■□■■■■■■■□■■■■■■■■■■■□□□□■■■□■■■	2	MTQ(2)	T2-T3
937	■■■■■■■■■■■■■■■■■■■■■■■■■■■□□□□□■□■■■■■■■■■	3	THA(3)	MANU1
938	■□□■■■■□□□□■■■■■■■■■■■■■■■■■□□□□■□■■■■■■■■■	2	THA(2)	EAI5
939	■■■■■■■□■■■■■■■■■■■■■■■■■■■■□□□□■□■■■■■■■■■	2	THA(2)	EAI5
940	□□□□□□□□□□□□□□□□□□□□□□□□□□□□□□□□□□■■■□□□□□■	4	ITA(1) THA(2) VNM(1)	BEIJING
941	□□□□□□□□□□□□□□□□□□□□□□□□□□□□□□□□□□■■■■■■□■■	6	GBR(1) MEX(1) THA(2) USA (2)	BEIJING
942	■■■■■■■■■■■■■■■■■■■■■■■■□□□■■■■■□□□□■■■□■■■	3	THA(3)	T2
943	■■■■■■■■■■■■■■■■■■■■■■□■■■■■■■■■□□□□■■■□■■■	8	AUT(1) FXX(1) NZL(2) THA(3) USA(1)	T2-T4
944	■■■■■■■■■■■■■■■■■■■■■■■■■□□□□□□□□□□□□□□■■■■	4	MTQ(1) MYS(2) THA(1)	U
945	■■■■■□□■■■■■■□□□■■■■■■■■■■■■□□□□■□■■■■■■■■■	2	THA(2)	EAI5
946	■■■■■■■■■■■■■■■■■■■■■■□□□□□□□□□■□□□□■■■■■■■	3	IDN(1) NLD(1) THA(1)	H3
947	■■■■■■■■■■■■■■■■■■■■■■■■■■■■□□□□■□■■■■□□■■■	5	DNK(1) IND(1) MYS(1) THA(1) USA (1)	EAI5
948	■■■■■■■■■■■■■■■■■■■■■■■□□□□□□□□■□□□□■■□□□■■	6	MEX(1) PER(2) USA(3)	H3
949	■■■■■■■■■■■■■■■■■■□□□□□■■■■■■■□■□□□□■■■■■■■	2	PER(2)	H3
950	■■■□□□□□□□□□□■■■■■■■□□□□■■■■■■■■□□□□■■■■■■■	7	AUS(1) USA (6)	LAM9
951	■■■■■■■□□□□■■■■■■■■■■■■■■■■■□□□□■□■■■■■■■■■	18	AUS(1) DNK(1) MYS(9) NZL(2) THA(4) USA (1)	EAI5
952	■■□□□□□■■■■■■■■■■■■■■■□□□□□□□□□□□□■■■■■■■■■	4	BGD(1) SWE(1) USA (2)	CAS
953	■■■■■■■■■□■■■■■■■■■□■■■■■■■□■■■■□□□□■■■■■■■	2	AUT(1) USA(1)	T1
954	■■■□□□□■■■■□■■■■■■■■■■□□□□□□□□□□□□■■■■■■■■■	3	GBR(1) USA (2)	CAS1_DELHI
955	□□□□□□□□□□□□□□□□□□□□□□□□■■■■■■■■■■■■■■■■■■■	3	USA (2) VNM(1)	U
956	■■■■■■■■■■■■■■■■■■■■■■■■■■■■■■■■□□□□□□□□□■■	4	BRA(1) USA(3)	U
957	■■□■■■■■□■□■□□□□□■■■■■■■■■■□□■■■■■■■■■□□□□□	2	USA (2)	BOV
958	■■■■■■■■■■■■■■■■■■■■■■■■■■□□□□□□■□■■■■■□■■■	4	GBR(1) GUF(2) USA (1)	MANU1
959	■■■■■■■■■■■■■□□□□■■■■■■■■■■■□□□□■□■■■■■□■■■	2	GBR(1) USA (1)	EAI1_SOM
960	■□□■■■■■■■■■□■■■■■■■□□□□■■■■■■■■□□□□■■■■■■■	8	USA (2) VEN(6)	LAM5
961	■□□□□□■■■■■■■■■■■■■■□□□□■■■■■■■■□□□□■■■■■■■	3	GEO(1) SEN(1) USA (1)	LAM9
962	■■■■■■■■■■■■■■■■■■■■■■■■■■■■□□□□■□■■□□□□■■■	5	MYS(3) USA (2)	EAI5
963	■■■■■■■■■■■■■■■■■■■■■□■■■■■■■■□■□□□□■■■■■■■	5	CZE(1) ITA(2) NLD(1) USA (1)	H3
964	■□□□■■■■■■■■■■■■■■■■□□□□■■■■■■■■□□□□■■■■■■■	5	FXX(2) MAR(1) SWE(1) USA (1)	LAM9
965	■□□□■■■■■■■■■■■■■■■□□□□□■■■■■■■■□□□□■■■■■■■	2	FXX(2)	T1
966	■■■■■■■□■■■■■■■■■■■■■■■■■■■■■■■■□□□□■■■■■■■	6	ARG(1) EST(1) FXX(2) MUS(1) NLD(1)	T1
967	□□□□□□□□□□□□□□□□□□□□□□□□■■□□□■■■■■■■■■□□□□□	4	ESP(3) FXX(1)	PINI
968	□□□□□□□□□□□□□□□□□■■■■□□□■□■■■■■■■■■■■■□□□□□	2	FXX(2)	BOV
969	□□□□□□□□□□□□□□□□□■■■■□□□■■■■■■■■■■■■□□□□□□□	10	FXX(10)	BOV
970	□□□□□□□□□□□□□□□□□■■■■□□□■■■■■■■■■■■■■■□□□□□	28	FXX(28)	BOV
971	□□□□□□□□□□□□□□□□□■■■■■□□■■■■■■■■■■■■□□□□□□□	3	FXX(3)	BOV
972	□□□□□□□□□□□□□□□□□■■■■■□□■■■■■■■■■■■■■■□□□□□	4	ESP(1) FXX(3)	BOV
973	□□□□□■■■□■■■■■■□■■■■■■■■■■■■■■■■■■■■■■□□□□□	2	FXX(2)	BOV
974	□□□■□□■■□■■■■■■□■■■■■■■■■■■■■■■■□■□□■■□□□□□	13	FXX(13)	BOV
975	□□□■□□■■□■■■■■■□■■■■■■■■■■■■■■■■□■■■■■□□□□□	16	FXX(16)	BOV
976	□□□■■■■■□■■■■■■□■■■■■■■■■■■■■■■■■■■■■□□□□□□	5	FXX(5)	BOV
977	□□□■■■■■□■■■■■■□■■■■■■■■■■■■■■■■■■■■■■□□□□□	5	?(1) ARG(1) DEU(1) FXX(1) ITA(1)	BOV
978	□■□□□□□□□□□□□□□□■■■■■■■■■■■□■■■□■■■■■■□□□□□	8	AUT(1) FXX(7)	CAP
979	□■□□□□□□□□□□□□□□■■■■■■■■■■■■■■■■■■■■■■□□□□□	3	BEL(1) FXX(1) ITA(1)	CAP
980	■□□□□□□□□□□□□□□□□□□□□■■■■■■■■■■■■■■■■■□□□□□	19	ESP(3) FXX(15) NLD(1)	BOV

981	■□□□□■■■□■■■■■■□■■■■■■■■■■■■■■■■■■■□□□□□□□	5	FXX(5)	BOV
982	■□□□□■■■□■■■■■■□■■■■■■■■■■■■■■■■■■■■■■□□□□□	2	ESP(1) FXX(1)	BOV
983	■■□□□□□□□□■■■■■□■■■■■■■■■■■■■■■■■■■■■■□□□□□	3	FXX(3)	BOV
984	■■□□□□□■□■■■■□■□■■□■■■■■■■■■■■■■■■■■■■□□□□□	2	FXX(2)	BOVIS1
985	■■□□□□■■□■■■■■■□■■■■□■■■■■■■■■■□□■■■■■□□□□□	3	FXX(3)	BOVIS1
986	■■□□□□■■□■■■■■■□■■■■■■■■■■■■■■■■■■■■■■□□□□□	3	DEU(1) FXX(2)	BOVIS1
987	■■□□□■■□□□□■■■■□■■■■■■■■■■■■■■■■■■■■■■□□□□□	2	FXX(2)	BOV
988	■■□□□■■■□■■□□□□□□■■■■■■■■■■■■■■■■■■■■■□□□□□	2	FXX(2)	BOV
989	■■□□□■■■□■■■□■■□■■■■■■■■■■■■■■■■■■■■■■□□□□□	6	? (1) FXX(2) USA(3)	BOVIS1
990	■■□□□■■■□■■■■■■□■□■■■■■■■■■■■■■■■■■■■■□□□□□	21	BEL(4) FXX(17)	BOVIS1
991	■■□□□■■■□■■■■■■□■■■■■□■■■■■■■■■■■■■■■■□□□□□	30	?(1) BEL(1) FXX(28)	BOVIS1
992	■■□□□■■■□■■■■■■□■■■■■■■■■■■■■□■■■■■■■■□□□□□	2	FXX(2)	BOVIS1
993	■■□□□■■■□■■■■■■□■■■■■■■■■■■■■■■□■■■■■■□□□□□	3	FXX(3)	BOVIS1
994	■■□□□■■■□■■■■■■□■■■■■■■■■■■■■■■■□□■■■■□□□□□	2	FXX(2)	BOVIS1
995	■■□□□■■■□■■■■■■□■■■■■■■■■■■■■■■■■■□■■■□□□□□	4	FXX(4)	BOVIS1
996	■■□□■■■■□□■■■■■□■■■■□■■■■■■■■■■■■■■■■■□□□□□	2	FXX(2)	BOV
997	■■□□■■■■□■■■■■■□■■■■■■■■■■■■■■■■■■■■■■□□□□□	28	ARG(2) BRA(1) FXX(20) MEX(5)	BOVIS1
998	■■□■□□■□□■■■■■■□■■■■■■■■■■■■■■■■□■■■■■□□□□□	2	FXX(2)	BOV
999	■■□■□□■■□■■■■■■□□□□□□■■■■■■■■■■■□■■■■■□□□□□	22	FXX(22)	BOV
1000	■■□■□□■■□■■■■■■□■■■■■■■■■■□□□■■■□■■■■■□□□□□	19	FXX(19)	BOVIS1
1001	■■□■□□■■□■■■■■■□■■■■■■■■■■■■■■■■□■■■□■□□□□□	4	FXX(4)	BOVIS1
1002	■■□■□□■■□■■■■■■□■■■■■■■■■■■■■■■■□■■■■■□□□□□	9	FXX(9)	BOVIS1
1003	■■□■□■■■□■■■■■■□■■■■■■■■■■■■■■■■■■■■■■□□□□□	6	FXX(4) MWI(2)	BOVIS1
1004	■■□■■□□■□■■■■■■□■■■■■■■■■■■■■■■■■■■■■■□□□□□	6	FXX(4) ITA(2)	BOVIS1
1005	■■□■■■□■□■■■■■■□■■■■■■■■■■■■■■■■■■□■■■□□□□□	2	ESP(1) FXX(1)	BOVIS1
1006	■■□■■■■□□□■■■■■□■■■■■■■■■■■■■■■■■■□□□□□□□□□	2	FXX(2)	BOV
1007	■■□■■■■□□□■■■■■□■■■■■■■■■■■■■■■■■■■■■■□□□□□	2	FXX(2)	BOV
1008	■■□■■■■■□■□■■■□□■■■■■■■■■■■■■■■■■■■■■■□□□□□	6	ARG(3) FXX(1) GBR(1) MEX(1)	BOV
1009	■■□■■■■■□■■■□□■□■■■■□■■■■■■■■■■■□■■■■■□□□□□	2	FXX(2)	BOVIS1
1010	■■□■■■■■□■■■□■■□■■■■□■■■■■■■■■■■■■■■■■□□□□□	4	ESP(1) FXX(3)	BOVIS1
1011	■■□■■■■■□■■■■□□□■■□■■■■■■■■■■■■■■■■■■■□□□□□	3	FXX(2) NLD(1)	BOV
1012	■■□■■■■■□■■■■■□□□□□□□□□□□□□□□□□□□■■■■■□□□□□	2	FXX(2)	BOV
1013	■■□■■■■■□■■■■■□□□□□□□□□□■■□□□□■■□■■■■■□□□□□	6	FXX(6)	BOV
1014	■■□■■■■■□■■■■■□□■■■■□□■■■■■■■■■■□■■■■■□□□□□	4	FXX(4)	BOV
1015	■■□■■■■■□■■■■■■□□□□□□■■■■■■■■■■■■■■■■■□□□□□	5	FXX(5)	BOV
1016	■■□■■■■■□■■■■■■□■□■■■■■■■■■■■■■□■■■■■□□□□□	13	FXX(13)	BOVIS1
1017	■■□■■■■■□■■■■■■□■■□□□□□■■■■■■■□■■■■■■■□□□□□	3	FXX(3)	BOVIS1
1018	■■□■■■■■□■■■■■■□■■□■■■■■■■■■■■■□■■■■■■□□□□□	2	ESP(2)	BOVIS1
1019	■■□■■■■■□■■■■■■□■■□■■■■■■■■■■■■■■■■■■■□□□□□	6	DEU(2) DNK(1) FXX(3)	BOVIS1
1020	■■□■■■■■□■■■■■■□■■■□□□□■■■■■■■■■■■■■■■□□□□□	7	FXX(7)	BOVIS1
1021	■■□■■■■■□■■■■■■□■■■■□■■■■□□□□□□■■■■■■■□□□□□	6	BRA(2) FXX(2) MEX(2)	BOVIS1
1022	■■□■■■■■□■■■■■■□■■■■□■■■■■■■■■■□□□■■■■□□□□□	13	DEU(6) FXX(7)	BOVIS1
1023	■■□■■■■■□■■■■■■□■■■■□■■■■■■■■■■□■■■■■■□□□□□	4	FXX(3) ZAF(1)	BOVIS1
1024	■■□■■■■■□■■■■■■□■■■■□■■■■■■■■■■■□□□■■■□□□□□	2	FXX(2)	BOVIS1
1025	■■□■■■■■□■■■■■■□■■■■□■■■■■■■■■■■□□■■■■□□□□□	3	FXX(3)	BOVIS1

1026	■■□■■■■■□■■■■■■□■■■■□■■■■■■■■■■■■■□□■■□□□□□	3	FXX(3)	BOVIS1
1027	■■□■■■■■□■■■■■■□■■■■□■■■■■■■■■■■■■□■■■□□□□□	2	FXX(1) SAU(1)	BOVIS1
1028	■■□■■■■■□■■■■■■□■■■■□■■■■■■■■■■■■■■■□□□□□□□	2	FXX(1) NLD(1)	BOV
1029	■■□■■■■■□■■■■■■□■■■■■□■■■■■■■■■■□■■□■■□□□□□	3	FXX(3)	BOVIS1
1030	■■□■■■■■□■■■■■■□■■■■■□■■■■■■■■■■■■■■■■□□□□□	27	DEU(1) FXX(26)	BOVIS1
1031	■■□■■■■■□■■■■■■□■■■■■■■□■□□□□■■□□■■□■■□□□□□	2	FXX(2)	BOVIS1
1032	■■□■■■■■□■■■■■■□■■■■■■■■■■□□□□□□□□■■■■□□□□□	2	FXX(2)	BOVIS1
1033	■■□■■■■■□■■■■■■□■■■■■■■■■■■□□□■■■■□■■■□□□□□	6	DEU(1) FXX(5)	BOVIS1
1034	■■□■■■■■□■■■■■■□■■■■■■■■■■■■■□□□□□□□■■□□□□□	3	FXX(3)	BOVIS1
1035	■■□■■■■■□■■■■■■□■■■■■■■■■■■■■□□■□■■■■■□□□□□	2	FXX(2)	BOVIS1
1036	■■□■■■■■□■■■■■■□■■■■■■■■■■■■■□□■■■■■■■□□□□□	4	FXX(4)	BOVIS1
1037	■■□■■■■■□■■■■■■□■■■■■■■■■■■■■□■■■■■■■■□□□□□	3	FXX(2) USA (1)	BOVIS1
1038	■■□■■■■■□■■■■■■□■■■■■■■■■■■■■■□■■■□■■■□□□□□	8	FXX(8)	BOVIS1
1039	■■□■■■■■□■■■■■■□■■■■■■■■■■■■■■□■■■■□■■□□□□□	3	FXX(3)	BOVIS1
1040	■■□■■■■■□■■■■■■□■■■■■■■■■■■■■■□■■■■■■■□□□□□	17	AUT(1) DEU(1) FXX(15)	BOVIS1
1041	■■□■■■■■□■■■■■■□■■■■■■■■■■■■■■■□□□■■■■□□□□□	9	FXX(9)	BOVIS1
1042	■■□■■■■■□■■■■■■□■■■■■■■■■■■■■■■□■□■■■■□□□□□	7	FXX(7)	BOVIS1
1043	■■□■■■■■□■■■■■■□■■■■■■■■■■■■■■■■□■■□■■□□□□□	5	FXX(5)	BOVIS1
1044	■■□■■■■■□■■■■■■□■■■■■■■■■■■■■■■■□■■■■■□□□□□	51	FXX(51)	BOVIS1
1045	■■□■■■■■□■■■■■■□■■■■■■■■■■■■■■■■■□□□■■□□□□□	7	BEL(2) FXX(5)	BOVIS1
1046	■■□■■■■■□■■■■■■□■■■■■■■■■■■■■■■■■□□■■■□□□□□	5	FXX(5)	BOVIS1
1047	■■□■■■■■□■■■■■■□■■■■■■■■■■■■■■■■■□■■■■□□□□□	13	ESP(1) FXX(12)	BOVIS1
1048	■■□■■■■■□■■■■■■□■■■■■■■■■■■■■■■■■■□■■■□□□□□	10	FXX(10)	BOVIS1
1049	■■■■■■■■■■■□□■■■■■■□■■□□■■■□□□□□■□■■■■■■■■■	2	GBR(2)	MANU1
1050	■■■■■■■■■■■■□■■□□□□□□□□□□□□□□□□□□□□□■■■■■■■	4	AZE(1) RUS(2) USA (1)	U
1051	■■■■■■■■■■■■■■■■■■■□□□■■■■■■■■■■□□□□■■■■■■■	12	AZE(1) NLD(1) POL(10)	T1
1052	■■■■■■■■□□■■■■□■■■□■■■■■■■■■■■■■□□□□■■■■■■■	2	EGY(1) FXX(1)	S
1053	■■■■■■■■■■■■■■□■■■■■□□■■■■■■■■■■□□□□■■■■■■■	2	EGY(1) FXX(1)	T1
1054	■■■■■■■■■■■■□■■■■■■■■■■■□□□□□□□■□□□□■■■□■■■	7	CAF(5) FXX(1) USA (1)	H3
1055	■■■■■■■■■■■■■■□■■■■■■■■■□□□□□□□■□□□□■■■□■□□	4	CAF(3) GBR(1)	H3
1056	■■■■■■■■■■□□■■■■■■■■■■■■■■■■■■■■□□□□■■■□■■■	3	CAF(1) CMR(1) USA(1)	T2
1057	□□□□□□□□□□□□□□□□■■■■■■□□□■■■■■■■□□□□■■■■■■■	3	CMR(3)	LAM10_CAM
1058	□□□□■■■■■■■■■■■■■■■■■■■■■■■■■■■■□□□□■■■□■■■	2	AUT(1) CMR(1)	T2
1059	■■■■■■■■■■■■■■■■■■■■■■■■■□□■□□□□■■■■■■■■■■■	3	GUF(1) VNM(2)	U
1060	■■■■■■■■■■■■■■■■■■■■■■■■■■□□□■■■□□□□■■■■■■■	2	VNM(2)	T1
1061	■■■■□■■■■■■■■■■■■■□■■■■■■■■■■■■■■□□□□■■■■■■■	2	MDG(1) USA (1)	X1
1062	■■■□□□□□□■■■■■■■□□□□□□□□■■■■□□□□■□■■■■■■■■■	3	GUF(1) MDG(1) MOZ(1)	EAI5
1063	■■■■■■■■□□■■■■■■■■■■■■■■■■■■■□■■□□□□■■■■■■■	23	DEU(1) DZA(22)	S
1064	■■■■■■□□■■■■■■■■■■■■□□□□■■■■■■■■□□□□■■■■■■■	16	BEL(4) DZA(4) FXX(2) MAR(6)	LAM9
1065	■■■■■■■■■■■■□□■■■■■■■■□□□■■■■■■■□□□□■■■■■■■	2	DZA(2)	LAM10_CAM
1066	■■■■■■■■■■■■■■□■■■■■□□□□■■■■□■■■□□□□■■■■■■■	5	DZA(1) GUF(1) SWE(1) USA(1) VEN(1)	LAM6
1067	■■■■■■■□□□□□□■■■■■■■■□□□■■■■■■■■□□□□■■■□□□□	8	DZA(1) FXX(1) USA(1) ZAF(5)	T1
1068	■■■■■■■■□□■■□■■■■■□■■■■■■■■■■■■■□□□□■■■■■■■	4	MAR(4)	S
1069	■■■■■■□□■■■■■■■■■■■■■■■■■■■■■■■■□□□□■■■■■■■	15	AUT(7) ESP(1) FXX(1) MAR(5) PRT(1)	T1
1070	■■■■■■■■■■■■■■■■■■■■□□□□■■■■■■■■□□□□□■■■■■■	8	AUT(1) BEL(1) BGD(1) HTI(1) ITA(1) MAR(1) USA (2)	U

1071	■■■■■■■■■■■■■■■□□■■■□□□□■■■■■■■■□□□□■■■■■■■	4	MAR(4)	LAM9
1072	■■■■■■■■■■■■□■■□□■■■□□□□■■■■■■■■□□□□■■■■■■■	3	MAR(3)	LAM5
1073	■■■■■■■■■■■□□■■■■■■■■■■■■■■■■■■■□□□□■■■■■■■	2	FXX(1) MAR(1)	T1
1074	■■■■■■■■■■■■■■■■■■■■□□□□■■■■■■■■□□□□■■■■■□■	7	DEU(2) ITAS(1) MAR(2) USA(2)	LAM9
1075	■■■■■■■■■■■■■■■□□□■■□□□□■■■■■■■■□□□□■■■■■■■	3	FXX(1) MAR(1) USA(1)	LAM9
1076	□□□□□□■■■■■■■■■■■■■■□□□□■■■■■■■■□□□□■■■■■■■	5	IDN(1) MOZ(1) RUS(2) USA (1)	LAM9
1077	■■■■■■■■■■■■■■■■■■□■■■■■■■■■■■■■□□□□■■■□■■■	4	AUT(1) IDN(2) VNM(1)	T2
1078	□■■■■■■■■■■■□□□□■■□■■■■■■■■■■■■■□□□□■■■□■■■	2	IDN(2)	T2
1079	■□■■■■■■■■■■■■■■■■■■■□□□□■■■■■■■□□□□■■■■■■■	3	IDN(2) MYS(1)	T1
1080	■■■■■■■■■■■■■■■■■□■■■■■■■■■■■■■■□□□□■■■□□□■	5	IDN(2) ITA(1) USA(2)	X1
1081	■■■■■■■■■■■■□■■■■□■■■■■■■■■■■■■■□□□□■■■□■■■	2	IDN(1) USA (1)	X1
1082	■■■■■■■■■■■■■■■■■■■■■■■■■■■■□□□□■□■■□■■□□■■	2	IDN(2)	EAI5
1083	■■■■■■■■■■■■□□□□□□□□□□□□□□□□□□□□□□□■■■■□■■■	6	IDN(1) MYS(2) USA (3)	U
1084	■■□□□□□□□□□□□□□□□□□□□□□□□□□□■■■■□□□□■■■■■■■	4	GUF(4)	T1
1085	■■■■■■■■■■■■■■■■■■■■■■■■■□□■■□□■□□□□■■■□■■■	2	FXX(1) MTQ(1)	H3
1086	■■■■□□□□□■■■□■■■■■■■□□□□■■■■■■■■□□□□■■■□■■■	3	MTQ(3)	LAM4-LAM5
1087	□□■■■■■■■■■■■■■■■■■■□□□□□■■■■■■■□□□□■■■■■■■	4	GLP(1) HTI(1) USA(2)	T1
1088	■■■■■■■■■■■■■■□■■■■■■■■■■■■■■■■■□□■■■■■■■■■	6	IND(5) PRT(1)	MANU2
1089	■□□□□□□■■■■■■■■■■■■■■■□□□□□□□□□□□□■■■■■■■■■	3	IND(2) USA (1)	CAS
1090	□□□■■■■■■■■■■■■■■■■■■■■■■■■■□□□□■□■■■■■■■■■	3	IND(2) NLD(1)	EAI5
1091	■■■□□□□■■■■■■■■■■■■■■■□□□□□□□□□□□□■□■■■■■■■	6	IND(2) PAK(1) USA(3)	CAS1_DELHI
1092	■■■□□□□■□■■■■■■■■■■■■■□□□□□□□□□□□□■■■■■■■■■	6	IND(2) ITA(1) USA (1) ZAF(2)	CAS1_DELHI
1093	■■■□□□□■■■■■■■■■■■■■□□□□□□□□□□□□□□■■■■■■■■■	5	GBR(1) IND(2) SAU(1) USA(1)	CAS
1094	■■■■■■■■■■■■■■■■■■■■■■■■■■■■□■■■□□■■■■■■■■■	2	IND(2)	MANU2
1095	□□□□□□□□□□□□■■■■■□■■■■■■■■■■■■■■□□□□■■■■■■■	2	IND(2)	X1
1096	■□■■■■■■■■■■■■■■■■■■■■■■■■■■■■■■□□■■■■■■■■■	2	IND(2)	MANU2
1097	■□□□□□□□□□□■■■■■■■■■■■■■■■■■□□□□■□■■□□□■■■■	2	IND(2)	EAI3_IND
1098	■■■■■■■■■■■■■■■■■■■■■■■■■■■■■■■■□□□□□■■□■■■	3	AUS(1) USA(2)	U
1099	■■■■■■■■□□□□□□□□□□□□□□□■■■■■■■■■□□□□■□□■■■■	3	USA(3)	T1
1100	□□□□■■■■■■■■■■■■■□■■■■■■■■■■■■■■□□□□■■□□□□■	30	USA(30)	X2
1101	□□□□□□□□□□□□■■■■■□■■■□□□□□□□□□□□□□□□□□□■■■	2	USA(2)	U
1102	■■□□□■■■■■■■■■■■■□■■■■■■■■■■■■■■□□□□■□□□□□■	2	USA(2)	X1
1103	■■■■■■□□□□□□□□□□□□■■■■□■■■■■■■■■□□□□■■■■■■■	2	USA(2)	T1_RUS2
1104	■■■■■■■■■■■■■■■■■■■■■■□■■■■■■□■■□□□□■■■■■■■	2	VEN(2)	T5
1105	■■■■■■■■■■■■■■■□■■■■■■■■■■■■■■■■□□□□■■■■■■■	5	CHN(1) PER(1) USA (3)	T1
1106	■■■■■■■■■■■■■■■■■■■■□□□□■■■■■■■■□□□□■□□□□□□	11	ITAS(1) PER(1) PRT(9)	LAM9
1107	■■■■■■■■■■■■□□□□□□□□□□□□■■■■■■■■□□□□■■■■■■■	3	FXX(1) ITA(1) PER(1)	T1
1108	□■□□□□□□□□□□□□□□■■□■■■■■□□□□□□□□□□□□□□□□□□□	4	AUT(1) DEU(3)	CAP
1109	□■□□□□□□□□□□□□□□■■■□□□□□□□□□□□□■■■■■■■□□□□□	5	AUT(5)	CAP
1110	□■■■■■■■□□□□□■■■■■■■■■■■■■■■■■■■□□□□■■■■■■■	20	AUT(20)	T1
1111	□■■■■■■■■■■■■■■■■■■■■■■■■■■■■■■■□□□□■■■■■□□	21	AUT(20) DEU(1)	T1
1112	■□□□□□□■■■■■■■■■■■■■■■■■■■■■■■■■□□□□■■■■■■■	3	AUT(2) USA (1)	T1
1113	■□□■■■■■□□■■■■■■■■■■■■■■■■■■■■■■□□□□■■■■■■■	10	AUT(8) BEL(1) ZAF(1)	S
1114	■□□■■■■■■□■■■■■■■■■■■■■■■■■■■■■■□□□□■■■□■■■	6	AUT(4) LVA(1) POL(1)	T2
1115	■□■■■■■■■■■■■■■■■■■■■■■■■■■■■■□□□□□□□□□□□□□	3	AUT(3)	U

1116	■■□□□□■■■■■■■■■■■■■■■■■■■■■■■■□■□□□□■■■■■■■	2	AUT(2)	H3
1117	■■□■■■■□□■■■■■■■■■■■■■■■■■■■□□□■□□□□■■■■■■■	3	AUT(1) RUS(2)	H4
1118	■■□■■■■■□■■□■■■□■■■■■■□■■■■■■■■■■■■■■■■□□□□□	3	AUT(2) USA (1)	BOVIS1
1119	■■■□□□□□□□□□□■■■■□■■■■■■■■■■■■■■□□□□■□□■■■■	3	AUT(1) USA (2)	X3
1120	■■■□□□□■■■■■■■□□□□□□□□□□□□□□□□□□□□□□□■■□■■■	3	AUT(1) DEU(1) PAK(1)	CAS
1121	■■■□□■■■■■■■■■■■■■■■■■■■■■■■■■■□□□□■■□□□■■	34	AUT(34)	T1
1122	■■■■■□■■■■■■■■■■■■■■■■■■■■■■■■■■□□□□■■■■■■■	15	ARG(1) AUT(8) BEL(2) CZE(1) FXX(1) USA (2)	T1
1123	■■■■■■□□□□□□□□□□□□■■■■■■■■■■■■■□□□□□□□■■■■■	5	AUS(1) AUT(1) BEL(1) USA(2)	T5-RUS2
1124	■■■■■■□□□□□□□□□□■■■■■■■■■■■■■■□■□□□□■■■■■■■	5	AUT(5)	H3
1125	■■■■■■□■■■■■□■■■■■■■■■■■■□□□□□□■□□□□■■■■■■■	4	AUT(4)	H1
1126	■■■■■■■□■■■□■■■■■■■■■■□■■■■■■■■■■□□□□■■■■■■■	2	AUT(1) GBR(1)	T5
1127	■■■■■■■■□□■■■■■■■■■□■■■■■■■■■■■■□□□□■■■■■■■	8	AUT(3) CAN(3) ITA(1) USA (1)	S
1128	■■■■■■■■□□■■■■■■■■■■■□■■□□□□■■■■□□□□■■■■■■■	4	AUT(4)	S
1129	■■■■■■■■□■■■■■■■■■■■■■■■■■■■■■■□□□□■■■■■■■	8	AUT(3) FXX(1) USA(4)	T1
1130	■■■■■■■■■□■■■■■■■■■■■■■■■■■■■■□□□□□□□□□□□□□	9	AUT(8) BEL(1)	U
1131	■■■■■■■■■■■□□□■■■■□■■■□□■■■■■■■■□□□□■□□■■■■	5	AUT(3) DEU(1) GLP(1)	T4-CEU1
1132	■■■■■■■■■■■□□■■■■■■■■■■■■■■■■■□■□□□□■■■■■■■	2	AUT(1) GBR(1)	H3
1133	■■■■■■■■■■■■□■■■■■□■■■□□■■■■■■■■□□□□■□□■■■■	2	AUT(1) USA (1)	T4-CEU1
1134	■■■■■■■■■■■■□■■■■■■■■■■■■■■■■□□□■□□□□■■■□■■■	27	ARM(2) AUT(1) GEO(5) RUS(18) USA (1)	H4
1135	■■■■■■■■■■■■□■■■■■■■■■■■■■■■■■■□■□□□□■■■□■■■	3	AUT(1) FXX(1) HUN(1)	H3
1136	■■■■■■■■■■■■■□□□□□□□□□□□■□□■■■□■□□□□■■■■■■■	7	AUT(6) USA(1)	H3
1137	■■■■■■■■■■■■■□□□□□□□□□□■■□■■■■■■□□□□■■■■■■■	4	AUT(4)	T1
1138	■■■■■■■■■■■■■■□■■■■■■■■■■■□□■■■■■□□□□■■■■■■■	4	AUT(3) ITA(1)	T1
1139	■■■■■■■■■■■■■■■■■■□■■■■■■□□□□□□■□□□□■■■■■■■	3	AUT(1) SWE(1) USA (1)	H1
1140	■■■■■■■■■■■■■■■■■■■□□□□□■□□□□■□■□□□□■■■■■■■	7	AUT(7)	H3
1141	■■■■■■■■■■■■■■■■■■■■■■□□□□■■■■■■□□□□■■■■■■■	2	AUT(1) USA (1)	T1
1142	■■■■■■■■■■■■■■■■■■■■■■■■■■■■■■□■□□□□□□□■■■■	6	AUT(6)	U
1143	■■■■■■■■■■■■■■■■■■■■■■■■■■■■■■□■□□□□□■■■■■■	3	AUT(1) POL(1) SWE(1)	U
1144	■■■■■■■■■■■■■■■■■■■■□□□□□□□■■■■■□□□□■■■■■■■	3	USA (2) VEN(1)	T1
1145	■■□■■■■■■■■■■■■■■■■■□□□□■■■□□□■■□□□□■■■■■■■	2	HTI(1) USA (1)	LAM1
1146	■■■■□□□□■■■■□■■■■■■■■□□□□■■■■□■■■□□□□■■■■■■■	3	CUB(2) USA (1)	T3-OSA
1147	■■■■■■■■■■■■■■■■■■■■□□□□□□□■□■■■□□□□■■■■■■■	2	USA (1) VEN(1)	T1
1148	■■■■■■■■■■■■□■■■□□□■■■■■■■■■■■■■□□□□■■■■■■■	2	USA(2)	T3
1149	■■■■■■■■■■■■■■■■■■■■■■■■■■■■■■■■■■■■□■■□■■■	5	THA(1) USA (4)	U
1150	■■■□□□□□□□□□□■■■■□■■■■■■■■□□■■■■□□□□■■■■■■■	3	PER(1) USA (2)	X3
1151	■■■□□□□□□□■■■■■■■■■■■■□□□□□□□□□□□□□■■■■■■■■	3	GBR(1) USA (2)	CAS
1152	□□□□□□□□□□□□□■■■■■■■■■■■■■■■■■■■□□□□■■■■■■□	2	USA (2)	T1
1153	■■■■■■□■□■■■□□□□□□□□□□□□□□□□□□□□□□■■■■□□■■■	2	NLD(1) USA (1)	AFRI
1154	■■■■■■■■■■■■■■■■■■■■□□□□■■■■■■■■□□□□■■■■□■■	7	AUS(1) CZE(1) HTI(1) USA (4)	LAM9
1155	■□■■■■■■■■■■■■■■■■■■■■■■■□□□□□□■□□□□■■■■■■■	2	BRA(1) USA (1)	H1
1156	■■□□■■■■□□■■■■■■■■■■□□□□■■■■■■■■□□□□■■■■■■■	2	USA(2)	LAM9-S
1157	■■■□□□□□□□□□■■■■■□■■■■■■■■■■■■■■□□□□■■■■■■□	3	USA(3)	X3
1158	■■□■□□□□□□□□□□■□■■□■■■■■■■■■■■■■■■■■■■□□□□□	5	ARG(2) USA (3)	BOVIS3
1159	■□□□■■■■■■■■■■■■■■■■■■■■■■■■■■□■□□□□■■■■■■■	5	USA (5)	H3
1160	■■■■■■■■■■■■□■■■■■□■□□□□■■■■■■■■□□□□■■■■■■■	3	USA (2) VEN(1)	LAM5

1161	■■■■■■■■■■■■■□□□□□□□□□□□■■■■■■■■□□□□■□■□■■■	2	USA (2)	T2
1162	□□□□□□□□□□□□□□□□□□□□□□□□□□□□□□□□□□■□□□■■■■■	5	MYS(1) USA (4)	BEIJING
1163	■■□■■■■■■■■■□■■■■■■■■■■■■■■■■■■■□□□□■■■■■■■	9	BRA(5) USA (4)	T3
1164	■■□□■■■■■■■■■■■■■■■■■■■■■■■■■■□□□□□□□■■■■■■	4	USA (4)	U
1165	■■■■■■■■■■■■□■■■■■■■■□■■■■■□□□■□□□□■■■■■■■	3	RUS(1) USA (2)	H4
1166	■■■■■■■■■□■■■■■■■■■■■■■■■■■■■■■■□□□□■■■■■■■	8	MWI(1) USA (5) VEN(2)	T1
1167	■■■■■■■■■■■■□□□□■■■■■□■■■■■■■■■■□□□□■■■■■■■	3	USA (3)	T1
1168	□□□□□□□□□□□□□□□□□□□□□□□□□□□□□□□□□□■■■■□□■■■	2	USA (2)	BEIJING
1169	■■□■■■■■■■■■□■■■■■■□□■■■■■■■□□□□■□■■■■■■■■■	5	PHL(4) USA (1)	EAI2_MANILLA
1170	■■□■■■■■□□□■■■■■■■■□□■■■■■■■□□□□■□■■■■■■■■■	3	PHL(2) USA (1)	EAI2_MANILLA
1171	■■□■■■■■■■■■■■■■■■■□□■■■■■■■□□□□■□■□■■■■■■■	2	PHL(1) THA(1)	EAI2_MANILLA
1172	□□□□□□□□□□□□□□□□□□□□□□□□□□□■□□□□□□■■■■■■■■■	3	EST(3)	U
1173	■■■■■■□□□□□□□□□□□□■■■■■■■■■■■■■■□□□□■■■□■■■	5	DEU(1) EST(1) FIN(1) RUS(1) SWE(1)	T1_RUS2
1174	■■■■■■■□□■■■■■■■■■■■■■□■■■■■□□□■□□□□■■■■■■■	2	EST(2)	H4
1175	■■■■■■■□□■■■■■■■■■■■■■■■■■■■□□□■□□□□□■■■■■■	4	EST(4)	U
1176	■■■■■■■■■■■■■□■■■■■■□□□□■■■■■■■■□□□□■■■■■■■	11	ARG(1) EST(1) GEO(3) IDN(4) MDG(1) NLD(1)	LAM9
1177	■■■■■■■■■■■■■■■■■■■■■■■□■■■■■■□□□□□□□□□□□□□	3	EST(3)	U
1178	■■■■■■■■■■■■■■■□□■□□□□□□□□□□□□□□□□□□■■■□■■■	2	GNB(1) NLD(1)	U
1179	■■■■■■■■■■■■■■■■■□■■■■■■■■□□■■■■□□□□■■■■■■■	2	HND(1) USA (1)	X1
1180	■■■■■□□■■■■■■■■■■■■■■■■■■■■■□□□□■□■■■■■□□□□	2	SWE(1) USA (1)	EAI5
1181	■■□■■■■■□■■■■■■□■■■■■■□■■■■■■■■■■■■■■■□□□□□	4	BEL(2) SWE(2)	BOVIS1
1182	■□□■■■■■■■■■■■■■■■■■■■■■■■■■□□□□■□■■■■■□■■■	2	SWE(2)	EAI1_SOM
1183	■■■■■■■■■■■■■■■■■■■■■■■■■■■■□□□□■□■■□■■□■■■	3	DNK(1) NLD(1) SWE(1)	EAI1_SOM
1184	□□□□□□□□□□□□□□□□■□□□□□□□□□□□□□□□□□■■■■■■■■■	3	FXX(1) SWE(1) VNM(1)	U
1185	■■□■■■■■□■■□■■■□■■■■■■■■■■■■■■■■■■■■■■□□□□□	2	FXX(1) SWE(1)	BOVIS1
1186	■■■■■■■■■■■■■■■■■■■■■■■■■■■■□□□□□□■■■■□■■■■	4	NLD(1) THA(3)	U
1187	■■□■■■■□□□□□□□□□□□□□□□□□□■■■□□□□□□■■■■■■■■■	4	THA(4)	U
1188	■■■■■■■■■■■■■■■■■■■■■■■■■■■□□□□□□□■■■■■■■■■	2	THA(2)	U
1189	■■□■■■■■■■■■■■■■■■■□□■■■■■■■□□□□□□■■■■■■■■■	4	MYS(2) THA(1) USA (1)	U
1190	■■■■■■■■■■■■□□□□□□■■■■■■■■□■□□□□■□■■■■■■■■■	8	VNM(8)	EAI5
1191	■■■■■■■■■■■■□■■■■■■■■■■■■□■■■■■□□□□■■■□■■■	2	VNM(2)	T2-T3
1192	■■■■■■■■■■■■■■■■■■■■□■■■■■■■■■■■□□■■■■■■■■■	4	GEO(1) IND(1) MDG(1) USA (1)	MANU2
1193	■■■■■■■■■■■■■■■■■■■■□■■■■■■■■■■■■■□■■■■■■■■■	2	IND(2)	MANU1
1194	■■■■■■■■■■■■■■■■■■■■□■■■■■■■■■■■■■□□□■■■■■■■	2	IND(2)	CAS
1195	■■■■■■■■■■■■■■□■■■■□□■■■■■■■■■■■□□■■■■■■■■■	2	IND(2)	MANU2
1196	■■■■■■■■■■■■■■■■■■■■■■■■■■■■■■■■□□□■■■■■■■■	3	IND(2) USA(1)	U
1197	■■■□□□□■■■■■■■■■■■■■■■□□□□□□■□□□□□■■□□■■■■■	21	NOR(21)	U
1198	■■■□□□□■■■■■□■■■■■■■■■□□□□□□□□□□□□■■□□■■■■■	6	DEU(1) GBR(1) SAU(1) SDN(3)	CAS1_DELHI
1199	■■■□□□□■■■■■■■■■■■■■■■□□□□□□□□□□□□□■□□■■■■■	3	SDN(1) USA (2)	CAS
1200	■■■□□□□■■■■■■■■■■■■■■■□□■■■■■■■■■■■■■■■■■■■	3	SDN(1) USA (2)	U
1201	■■□■■■■■■■■□□■■■■□□■□□□□■■■■■■■■□□□□■■■■■■■	2	CMR(1) SDN(1)	LAM1
1202	□□□□□□□□□□□□□□□□□□□□□□□□□■■■■■■■□□□□■■■■■■■	4	BRA(2) LBY(1) MDG(1	T1
1203	■■■□□□□■■■■■■■■■■■■■□■□□□□□□□□□□□□■■□□■■■■■	2	AUT(1) LBY(1)	CAS1_DELHI
1204	■■■■■■■■■■■■■■■□□□□□□□□□□□□□□□□□□□□□■■■□■■■	3	LBY(1) USA (2)	U
1205	■■■■■■■■■■■■■■■■■■■■■■■■■□□□□□□■□□□□■■□■■■■	2	ITAS(1) USA (1)	H1

1206	■□□□■■■■■■■■■■■■■■■■■■■■■■■■□□□□□□□□□□■■■■■	2	ITAS(1) USA (1)	U
1207	■■■□■■■■■■■■■■■■■■■■■■■■■■■■■■□□□□□□■■■■■■■	2	ITAS(2)	U
1208	□□□□□□□□□□□□□□□□□□□□■□□□■□□□□□□■□□□□■■■■■■■	2	FXX(1) USA(1)	H1
1209	□□□□□□□□□□□□□□□□□□□■■■■■■■■■■■■■□□□□■■■■■■■	3	MEX(2) USA (1)	T1
1210	□□□□□■■■■■■■■■■■■■■■□□□□■■■■■■■■□□□□■■■■■■■	4	MEX(2) USA(2)	LAM9
1211	■□■■■■■■□□■■■■■■■■■■■■■■■■■■■■■■□□□□■■■■■■■	2	MEX(1) USA(1)	S
1212	■■□□□□□□□□□□□□□□□□□□□□□□□□□□□□■■□□□□■■■■□■■	2	MEX(2)	T1
1213	■■□■□□■■■■■■□■■■■■■■□□□□■■■■■■■■□□□□■■■■■■■	2	USA(2)	LAM2
1214	■■■■■■■■■■■□□□■■■■■■■■■■■■■■■■■■□□□□■■■■■■■	11	DEU(1) GUF(1) MDG(8) POL(1)	T1
1215	■■□■■■■■■■■■□■■■□■■■□□□□■■■■■■■■□□□□■■■■■■■	5	MEX(2) USA(2) VEN(1)	LAM2
1216	■■□■■■■■■■■■□■■■■■■■□□□□■■■■■■□□□□□□□■■■■■■	2	USA(2)	U
1217	■■□■■■■■■■■■■■■■■■■□□■■■■■■■□□□□■□■■□□■■□■■	2	USA(2)	EAI2_MANILLA
1218	■■■□□□□□□□□□■■■■■□■■■■■■■■■■■■■□□□□□■■■■■■■	2	GBR(1) USA(1)	U
1219	■■■□■■■■■■■■■■■■■■■□□□■□□■■■□■■■□□□□■■■■■■■	4	MEX(4)	T1
1220	■■■■□■■□□□■■■■■■■■■■□□□□■■■■■■■■□□□□■■■□□□□	2	USA(2)	LAM9
1221	■■■■□■■■■■■■■■□■■■■■■■■■■■■■■■■■□□□□■■■■■■■	2	FXX(1) MEX(1)	T1
1222	■■■■■■□□□□□■■■■■■■■■□□□□■■■■■■■■□□□□■■■■■■■	2	IDN(1) USA(1)	LAM9
1223	■■■■■■■■□□□□■■■■■■■■■■■■■■■■■■■■□□□□■■■■■■■	3	MDG(1) USA(1) USA (1)	T1
1224	■■■■■■■■□□□■■■■■■■■■□□□□■■■■■■■■□□□□■■■□□□□	2	USA(2)	LAM3
1225	■■■■■■■■□□■■■■■■■■■■■□□□□□□□□■■■□□□□■■■■■■■	2	MEX(2)	S
1226	■■■■■■■■■■■□□□■■■■■■■■■■■■■■■■■■■■■■■■■■■■■	4	USA(4)	U
1227	■■■■■■■■■■■■□■■■■■■□■■□■■■■■■■■■□□□□■■■■■■■	6	DEU(1) ESP(4) USA(1)	T5_MAD2
1228	■■■■■■■■■■■■■□□□□□□□□□□■■■■■■■■■□□□□■■■□■■■	2	AUS(1) MEX(1)	T2
1229	■■■■■■■■■■■■■□□□■■■■■■■■■■■■■■□■□□□□■■■■■■■	3	AUT(1) POL(1) USA(1)	H3
1230	■■■■■■■■■■■■■□■■■■■■■■■■■□□□□□□■□□□□■■■■■■■	3	ARG(1) NLD(1) USA(1)	H1
1231	■■■■■■■■■■■■■■■■□□□□□■□■■■■■■■■■□□□□■■■■■■■	2	USA(2)	T5
1232	■■■■■■■■■■■■■■■■□□□□□■■■■■■■□■■■□□□□■■■□■■■	3	USA(3)	T2
1233	■■■■■■■■■■■■■■■■■■□□□■■■■■■■■■■■□□□□■■■■■■■	3	MEX(2) USA(1)	T1
1234	■■■■■■■■■■■■■■■■■■■■□■□□■□□■■■□■□□□□■■■■■■■	6	USA(6)	H3
1235	■■■■■■■■■■■■■■■■■■■■■■□□■□□■■■□■□□□□■■■■■■■	2	MEX(1) USA (1)	H3
1236	■■■■■■■■■■■■■■■■■■■■■■■□□□□□□□□□□□□□■■□□□■■	13	MEX(4) USA(9)	U
1237	■■■■■■■■■■■■■■■■■■■■■■■□□■■■■□■■□□□□■■■■■■■	3	USA (3)	T1
1238	■■■■■■■■■■■■■■■■■■■■■■■■■■□■■■□■□□□□■■■■■■■	3	GLP(1) MEX(1) VEN(1)	H3
1239	■■■■■■■■■■■■■■■■■■■■■■■■■■■□□■■■□□□□□□□□■■■	2	MEX(1) USA(1)	U
1240	■■■■■■■■■■■■■■□□□□□□□□□□□□■■■■■■□□□□■■■■■■■	2	LVA(1) USA (1)	T1
1241	■■■■■■■■■■■■■■■■■■■■□□□□■■■■■■□□□□□□■■■■■■■	28	ARG(13) BRA(3) GUF(1) USA(2) ZAF(9)	U
1242	■■■■■■■■□□■■■■■■■■■■■■■■■■■■□□□■□□□□■■■■■■■	8	ITA(6) USA (2)	H4-S
1243	■■■■■■■■■□■■■■■■■■■■■■■■■■■■■■□■□□□□■■■■■■■	3	ARG(1) ITA(1) MYS(1)	H3
1244	■■■■■■■■□□■■■■■■■■■■■■■■■■■■■■□■□□□□■■■□■■■	2	CAN(2)	S
1245	■■■■■■■■□□■■■■■■■■■■■■■■■■■■□□□□□□□□□□□■■■■	3	CAN(2) USA(1)	U
1246	■■■■■■■■■■■■■■■■■□■■■■■■■■■■■■□■□□□□■■■■■■■	8	CAN(7) EST(1)	H3
1247	■■■■■■■■■■■■■■■■■■■■□□□□■■■■■■■■□□■■■■■■■■■	10	IND(1) LVA(1) RUS(8)	MANU2
1248	■■■■■□□□□□□□□□□□■■■■■■■■■■■■■■■■□□□□■■■■■■■	3	RUS(3)	T1
1249	□□□□□□□■■■■■■■■■■■■■□□□□■■■■■■■■□□□□■■■■■■■	3	RUS(2) USA (1)	LAM9
1250	■■■■■■■■■■■■■■■■■■■■■□■■■■■■■■■□□□□□■■■■■■■	2	FXX(1) RUS(1)	U

1251	■■■■■■■■■■■■■■□■■■■■■■■■■■■■□□□□■□■■■■■□■■■	2	DNK(1) NOR(1)	EAI1_SOM
1252	■■■■■■■■■■■■■■■■■■□□■■■■■■■■■■■■□□□□■■■■■■■	2	AUS(1) RUS(1)	T1
1253	■■□■■■■■□□■■■■■■■■■■■□■■■■■■■■■■□□□□■■■■■■■	7	ARG(1) DEU(1) FXX(1) RUS(2) TUR(2)	S
1254	■■■■■■■■■■■■■■■■■□■■■■■■■■■□□□■■□□□□■■■■■■■	3	NLD(1) RUS(1) USA(1)	X1
1255	□□■■■■■■■■■■■■■■■■■■□■□■■■■■■■■■□□□□■■■■■■■	2	NLD(1) RUS(1)	T5
1256	■■■■■■□□□□□■■■■■■■■■■■■■■■□□□□□□■□□□□■■■■■■■	2	RUS(1) USA (1)	H1
1257	■■■■■■■■■■■■□■■■■□□□□□□□□□□□□□□□□□□□□□□□□□□	2	AUT(1) RUS(1)	U
1258	■■■□□□■■■■■■■■■■■■□■■■□□■■■■■■■■□□□□■□□■■■■	42	CAN(41) DEU(1)	T1
1259	■■■■■■■■■■■■■□□□□□□□□□□□□□□□□■■■□□□□■■■■■■■	2	RUS(1) USA (1)	T1
1260	□□□□□□□□□□□□□□□□□□□□□□□□■■■■■□□■□□□□■■■■■■■	2	MEX(1) USA(1)	H3
1261	■■■■■■■■■■■■■■■■■■■□□□□□■□□■■■■■□□□□■■■□■■■	8	AUT(1) BEL(2) DNK(1) FXX(1) TUR(2) USA (1)	LAM7_TUR
1262	□□□□□□□□□□□□□□□□□□□□□□□□■■■□□□□□□□□□■■■■■■■	2	MEX(2)	U
1263	■□□■■■■■■■■■■■■■■■□■■■■■■■■■□□□□■□□□■■■■■■■	2	BEL(2)	CAS
1264	■■■□□□□■■■■■■■■■■■■■■■□□□□□□□□□□□□□□□□□□□□□	3	USA(3)	CAS
1265	■■■■■■■■□■■■■■■■■■■■■■■■■■■■■■■■□□□□■■■□■■■	3	ITA(1) USA(2)	T2
1266	■■■□□□□□□■■■■■■■■■■■■■□□□□□□□□□□□□■■■■■■■■■	2	USA(2)	CAS
1267	■■■■■■■□□□□□□■■■■■■■■■■■■■■■■■■■□□□□□■■□□□□	2	FXX(1) USA(1)	U
1268	■■■■■■■■■■■■■■□□□□□□□■□■■■■■■■■■□□□□■■■■■■■	6	IND(2) MYS(1) PAK(1) USA(2)	T5
1269	■□■■■■■□□■■■■■■■■■■■■■■■■■■■□□□■□□□□■■■■■■■	2	RUS(1) USA(1)	H4
1270	■■■■□■■■■■■■■■■■■□■■■■■■■■■■■■■■□□□□■■□□□□■	2	USA(2)	X2
1271	■■■■■■■■□□■■■■■■■■■■□■■■■■■■■■■■□□□□■■■■■■■	3	USA(2) VEN(1)	S
1272	■□■■■■■■■■■■□■■■■■■■■■■■■■■■■■■■□□□□■□■■■■■	2	HTI(1) USA(1)	T3
1273	■■■□□□□□□□□□■■■■■□■■■■■■■■■■■■■■□□□□■■■□■■■	4	NZL(2) USA(2)	X3
1274	■■■■■■■■■■■■■■■■■■■■■■■□□□□□□□□□□□□□□□□■■■■	2	FIN(1) USA(1)	U
1275	■■■■■■■■■■■■■■■■■■■■■■■■■□□□□□□□□□□□□■■■■■■	10	AUT(8) USA(2)	U
1276	■■■■■■■■■□□□□□■■■■■■■■■■■■■■□□□■□□□□■■■■■■■	5	LVA(1) RUS(2) USA(2)	H4
1277	■■■■■■■■■■■■■■■■■■□■□□□□■■■■■■■■□□□□■■■■■■■	6	ARG(2) BRA(2) USA(2)	LAM9
1278	□□□□■■■■■■■□■■■■■■■■■■■■■■■■■■■■□□□□■■■■■■■	6	AUT(1) CZE(1) ESP(1) ITA(1) POL(1) USA(1)	T1
1279	■■■□■■■■■■■■■■■■■■■■■■□■■■■■■■■■□□□□■■■■■■■	4	CZE(2) USA(2)	T5
1280	□□□□□□□□□□□□□□□□□□□□□□□□■■■■■■■■□□□□■■■■■■□	4	ARG(1) CZE(1) EGY(1) USA(1)	T1
1281	■■■■■■■■■■□■□■■■■■■■■■■■■■■■□□□■□□□□■■■■■■■	3	CZE(1) MNG(1) USA(1)	H4
1282	■■■■■■■■■■■■■■■■■■■□□□□□□□□□□□□■□□□□■■■■■■■	7	CZE(1) ITA(5) USA(1)	H3
1283	■■■■■■■■■■■■■■■■■■■■■■■■■■■□□□□■□□□□■■■■■■■	3	HTI(1) USA(2)	H3
1284	■■■■□□□□■■■■□□□□■■■■■■■■■■■■■■■■□□□□■■■■■■■	4	BEL(2) GUF(1) USA(1)	T1
1285	■□■■■■■■■■■■■■■■■□■■■■□■■□■■□□□□■□■□■■■□■■■	2	NOR(2)	EAI1_SOM-EAI6
1286	■□□■■■■□□□□■■■■■■■■■■■■■■■■■■□□□■□■■■■■■■■■	2	NOR(2)	MANU1
1287	■□□□□□□□□□□□□■■■■■■■■■■■■■■■■□□□■□■■■■■■■■■	4	NOR(4)	MANU1
1288	■■■■■■■■■■□□■■■■■■■■□□□□■■■■■■■■□□■■■■■■■■■	4	RUS(4)	MANU2
1289	■■■■■■■■■■■■■■■■■■■■■□□■■■■■■■■■□□■■■■■■■■■	2	RUS(1) SDN(1)	MANU2
1290	■■■■■■■■■■■■■■■■■■■■□□□□■■■■■■□■□□■■■■■■■■■	3	RUS(3)	MANU2
1291	■■■■■■■■■■■■□■■■■■■■■■■■■■■■□□□■□□■■■■■■■■■	3	RUS(2) USA (1)	MANU2
1292	■■□■■■■□□■■■■■■■■■□■■■■■■■■■□□□■□□□□■■■■■■■	2	AUT(1) RUS(1)	H4
1293	■■□■■■■■□□□■■■■■■■■■□□□□■■■■■■■■□□□□■■■■■■■	3	USA (1) ZAF(2)	LAM3
1294	■■■■■■■□□□□■■■■■■■■□□□□□■■■■■■■■□□□□■■■■■■■	2	USA (1) ZAF(1)	T1
1295	■■■■■■■■□□□■■■□□■■■■□□□□■■■■■■■■□□□□■■■■■■■	4	ARG(2) ZAF(2)	LAM3

1296	■■■■■■■■□□■■■■■□□■■■■■■■■■■■■■■□□□□■■■■■■■	2	NLD(1) ZAF(1)	S
1297	■■□■■□■□□□□□■■■□■■□□□■■■■■■■■■■■■■■■■■□□□□□	2	GBR(2)	BOVIS2
1298	■■□■□□□■□□■□□□■■■■■■■■■■■■■■■■■■■■■■■■□□□□□	2	MEX(2)	BOV
1299	■□□■□□□□□□□□□□□□■■■■■■■■■■■■■■■■■■■■■■□□□□□	4	MEX(4)	BOV
1300	□□□□□□□□■■■■■■■■■□■■■■■■■■■■■■■■■■□□□□■■□□□□■	4	USA(4)	X2
1301	■□■■■■■■■■■■■■■■■■■■■■■■■■■■■■■■□□□□■■□□□□■	3	IND(1) ZAF(2)	T1
1302	■□■■■■■■■■■■■■■■■■■■■■■■■■■■■■■■□□□□■■■□■■■	8	CMR(1) ITA(6) ZAF(1)	T2
1303	■■□□■□■□□□□□■■■□■■■■■■■■■■■■■■■■■■■■■■□□□□□	2	BEL(1) IRL(1)	BOV
1304	■■□■□□□□□□□□□□■□■■■■■□■■■■■■■■■■■■■■■■□□□□□	2	GBR(2)	BOVIS3
1305	□□□■■□■□□□□□■■□□■■■■■■■■■■■■■■■■■■■■■■□□□□□	7	BRA(3) MEX(4)	BOV
1306	□■□■■□■□□□□□■■□□■■■■■■■■■■■■■■■■■■■■■■□□□□□	2	GBR(1) MEX(1)	BOV
1307	□□□■■□■□□□□□■■□□□■■■■■■■■■■■■■■■■■■■■■□□□□□	3	MEX(3)	BOV
1308	■■□■■□■□□□□□■■□□■■■■■■■■■■■■■■□□□□□□■■□□□□□	2	MEX(2)	BOV
1309	□□□■■□■□□□■□■■□□■■■■■■■■■■■■■■■■■■■■■■□□□□□	3	MEX(3)	BOV
1310	■□□■■□■□□□■□□■□□■■■■■■■■■■■■■■■■■■□□■■□□□□□	2	MEX(2)	BOV
1311	□□□□□□□□□□□□□□□□□□□□□□□□□□□□□□□□□□■■■■■□□□□	4	AUS(1) IND(2) USA(1)	U
1312	■■■□□□□■■■■■■■■■■■■■■■□□□□□□□□□□□□■■□□■□■■■	2	IND(1) SWE(1)	CAS1_DELHI
1313	■■■■■■■■■■■■■■■■■■■■■■■■■□■■□□□□■□■■■■■□■■■	2	IND(1) NLD(1)	EAI1_SOM
1314	■■■□□□□■■□■■■■■■■■■■■■□□□□□□□□□□□□■■□□■■■■■	3	IND(3)	CAS1_DELHI
1315	■■■□□□□□□□□□■■□■■□■■■■■■■■■■■■■■□□□□■■□■■■■	2	FXX(1) IND(1)	X3
1316	■■■□□■■■■■■■■■■■■■■■■■■■■■■■□□□□■□■■■■■□■■■	2	IND(1) NLD(1)	EAI1_SOM
1317	■□■■■■■■■■■■■■□■■■■■■■■■■■■■■■■■□□□□■■□■■■■	2	IND(1) ROM(1)	T1
1318	■□■■■■■■■■■■■■□■■■■■■■■■■■■■■■■■□□□□■■■■■■■	6	EGY(1) GUF(1) IND(4)	T1
1319	■□■□□□□□□□■■■■□■■■■■■■□□□□□□□□□□□□■■■■■■■■■	2	IND(2)	CAS2
1320	■■■□□□□□□□□■■■■■■■■■■■□□□□□□□□□□□□■■■■■■■■■	2	IND(2)	CAS
1321	■■□■■■■■■■■■■■■■■■■■□□□□■■■■■■■■□□□□■■■□■■■	8	ARG(1) GUF(1) IND(1) PRT(3) USA(2)	LAM1-LAM4
1322	■■■■■■■■■■■■□□□□■■□□□■■■■■■■■■■■□□□□■■■■■■■	2	IND(1) USA (1)	T1
1323	■□■□□□□■■■■■■■□■■■■■■■□□□□□□□□□□□□■■■■□■■■■	2	IND(2)	CAS1_DELHI
1324	■■■■■■■■■■■■■■□□□□□□□■□□■■■■■■■■□□□□■■■■■■■	2	IND(1) USA (1)	T1
1325	■■■■■■■■■■■■■■□□□■□□□■□■□□□□□□□□□□□□■■■■■■■	2	IND(2)	U
1326	■■■■■■■■■□□□□■■■■■■■■■■■■■■■□□□□■□■■■■■□□□□	2	IND(1) PAK(1)	EAI5
1327	■■■□□□□■■■■□□■■■■■■■■■□□□□□□□□□□□□■■■■■■■■■	2	IND(1) PAK(1)	CAS1_DELHI
1328	■■■■■■■■■■■■■■■■■■□□□□■■■□□□□□□■□□□□■■■■■■■	4	HTI(2) USA(2)	H1
1329	■■■■■■■■■■■■■■■■■□■■■■■■■■■■□■■■□□□□■■■■■■■	32	USA (1) ZAF(31)	X1
1330	□■■■■■■■■■■■■■■■■■■■■■□■■■■■■■■■□□□□■■■■■■■	3	TUR(1) ZAF(2)	T5
1331	■■■■■■■■□□□■■■■■□■■□□□□□■■■■■■■■□□□□■■■■■■■	2	ZAF(2)	T1
1332	■■□■■■■■■■■■■■■■■■■■■■■■■■■■■■■■□□□□■■■□■■■	6	BRA(1) DEU(1) FIN(1) MDG(2) ZAF(1)	T2
1333	■■■■■■■■□□■■■■■□□□□□□□□□□□■■■■■■□□□□■■■■■■■	2	ZAF(2)	S
1334	■■■■■■■■□□■■■■■□□□□□□□□□□□■■■■■■□□□□■■■□■■■	3	USA(1) ZAF(2)	S
1335	■■■■■■■■□□□■■■■■■■■■□□□□■■■■■■■■□□□□■□□□□□□	2	ZAF(2)	LAM3
1336	■■□■■■■■□□■■■■■■■■■■■□□■■■■■■■■■□□□□■□□■■■■	2	ZAF(2)	S
1337	■■■■■■■■■■■■□□□□□□■■□□□□■■■■■■■■□□□□■■■■■■■	7	GUF(6) RUS(1)	LAM9
1338	■■■■■■■■■■■■■■■■■■■■■■■■■■■■■■■■■■■■■■□■■■■	4	DNK(1) JPN(1) MTQ(1) SLE(1)	U
1339	■■□■■■■■■■■■□■■■■■■■■■■■■■■■■■■■□□□□■■■□■■■	2	GUF(1) USA (1)	T2-T3
1340	■■■■■■■■■■■■■■■■□■■■■■□■■■■■□□□□■□■■□■■■■■■	4	GUF(4)	EAI6_BGD1

1341	■■■■■■■■■■■■■■■■■□■■■■□■■■■■■■■■■□□□□■■□□□□■	10	USA(10)	X2
1342	■□□■■■■■■■■■■■■■■■□□■■■■■■■■□□□□■□■■□□□■■■■	4	DNK(1) NLD(1) USA (2)	EAI3_IND
1343	■■■□□□□■■■■■□■■■■■■■■■□□□□□□□□□□□□■■■■■■■■■	4	BEL(1) NLD(1) USA(2)	CAS1_DELHI
1344	■■■□□□□■■■■■■■■■■■■□□■□□□□□□□□□□□□■■■■■■■■■	2	IND(1) NLD(1)	CAS1_DELHI
1345	■■■□□□□■■■■■■■■■■■■■■□□□□□□□□□□□□□□□□■■■■■■	6	BGD(1) NLD(1) SWE(1) USA (3)	CAS
1346	■■■□□□□■■■■■■■■■■■■■□■□□□□□□□□□□□□■■■■■■■■□	15	ITA(15)	CAS1_DELHI
1347	■■■■■■■■■■■■□□□□□□■■■■■■■■■■■■■■□□□□■■■■■■■	8	ITA(8)	T1
1348	■■■■■■■□□□□□□□■■■■■■■■■■■■■■■■■■■□□□□■■■□□□■	2	ITA(2)	T1
1349	□□□■□■■■■■■■■■■■■■■■■■■■■■■■■■■■□□□□■■■■■■■	8	ITA(8)	T1
1350	■■■■■■■■■□□□□□■■■■■■■■■■■■■■■■□□□□□□□□□□□□□	2	ITA(2)	U
1351	■■■■■■■■□□■■■■■■■■■■□■■■■■■■■■■□□□□□□■■■■■■■	3	ITA(3)	U
1352	■■■■■■■■■■■□■■■■■■■■■■■■□□□□□□□□□□□□□□□□□□□	2	ITA(2)	U
1353	■■■■■■■■■■■■■■■■■■■■■■■■■□□□□□□□□□□□□□□□□□□	4	ITA(4)	U
1354	■□■■■■■■□□□■■■■■■■■■□□□□■■■■■■■■□□□□■■■■■■■	2	ITA(2)	LAM3
1355	■■■■■■■■■■■■■■■■■■■□□□□□■■■■□■■■□□□□■■■□■■■	11	ARG(1) ITA(7) PER(1) USA (2)	T2
1356	■■■■■■■■□□■■■■■■■■■■■■■■■■■■■■■■□□□□■■■■□■■	7	BRA(1) ITA(5) MEX(1)	S
1357	■■■□□□□■■■■■■■■■■■■□□□□□□□□□□□□□□□■■□□□□■■■	2	ITA(2)	CAS
1358	■■■■■■■■■■□□□□■■■■■■■■■□□□□□□□■■□□□□■■■■■■■	3	ITA(3)	T1
1359	■□□■■■■■■■■■■■■■□□□■■■■■■□□□□□□■□□□□■■■■■■■	8	FXX(1) GBR(1) ITA(6)	H1
1360	■□■■■■■■■■■■■■■■■■■■■■■■■■■■■■□■□□□□■■□□■■■	3	ITA(3)	H3
1361	■■■■□■■■■■■■□■■■■■■■■■■■■■■■■■■■□□□□■■■□■■■	3	ITA(2) ITAS(1)	T2-T3
1362	■■□■■■■■■■■■■■■■■■■■■□□□□□□□□□□□□□□□□■■■■■■	2	ITA(2)	U
1363	■■■■■■■■■■■■■■■■■■■■■■■■■■■■□□□□□□□□□□■■■■■	3	AUT(1) ITA(2)	U
1364	□□□□□□□□□□□□□□□□□□□□□□□□□□□□□□□□□□■■■□□■■■■	4	IDN(1) KOR(1) MYS(2)	BEIJING
1365	□□□□□□□□□□□□□□□□□□□□□□□□■■■■□□□□■□■■■■■■■■■	4	MYS(2) PHL(2)	EAI5
1366	□□□□□□□□□□□□■■■■■□■■■■■■■■■■■■■■□□□□■■□■■■■	2	IDN(1) MYS(1)	X1
1367	□■■■■■■■■■■■□■■■■■■■□□□□■■■■■■■■□□□□■■■■■■■	2	IND(1) MYS(1)	LAM5
1368	■■■□□□□□■■■■■■■■■■■■■■□□■■■■■■■■■□□□□■■■■■■	2	MYS(2)	U
1369	■□□■■■■■■■■■■■■■■■■■■■■■■■■□□□□■□■■■■□■■■■	2	GBR(1) MYS(1)	EAI5
1370	■■■■□■■■■■■■■■■■■■■■■■□■■■■■■■■■□□□□■■■■■■■	2	MYS(2)	T5
1371	■■■■■□■■■■■■■■■■■■■■□□□□■■□■■■■■□□□□■■■■■■■	2	MYS(2)	LAM8
1372	■■■■■■■■■■□■■■■■■■■■■■■■■■■■□□□□■□■■■■■■■■■	3	MYS(1) USA (1) VNM(1)	EAI5
1373	■■■■■■■■■■■□■■■■■■■■■■■■■■■■□□□□■□■■■■■□■■■	2	MYS(1) USA (1)	EAI1_SOM
1374	■■■■■■■■■■■■■■■■■■■■■□□□□□□□□□□□□□■■■■■■■■■	3	MYS(3)	U
1375	■■■■■■■■■■■■■■■■■■■■■■□■□■■■□□□□■□■■■■■■■■■	3	AUS(1) MYS(2)	EAI6_BGD1
1376	■■■■■■■■■■■■■■■■■■■■■■■■■□■■□□□□■□■■□■■■■■■	2	MYS(2)	EAI5
1377	■■■■■■■■■■■■■■■■■■■■■■■■■■■■□□□□■□■□■■□■■■■	2	MYS(1) NLD(1)	EAI5
1378	■■■■■■■■■■■■■■■■■■■■■■■■■■■■■■■■■□□□■■■■■■■	2	ARM(1) IND(1)	CAS
1379	■■■■■■□■■■■■■■■■■■■■■■■■■■■■■■■■□□□■■■■■■■	3	ARM(3)	CAS
1380	■■■■■■■□□■■■■■■■■■■■■■■■■■■■□□□□■□□□■■■■■■■	2	ARM(2)	CAS
1381	■■■■■■■□□■■■■■■■■■■■■■■■■□■■□□□□■□□□■■■■■■■	6	ARM(6)	CAS
1382	■■■■■■■■■■■■■■■■■■■■■■■■■■□■■■□■□□□□■■□■■■■	2	ARM(2)	H3
1383	■■■■■■□■■■■■■■■■■■■■■■■■■■■■■■■■□□□□■■■□■■■	2	ARM(1) HTI(1)	T2
1384	□□□□□□□□□□□□□□□□□□□■□■■■■■■■□□□■□□□□■■■■■■■	2	ARM(2)	H4
1385	■□■□■■■■■■■■■■□■■■■■■■■■■■□■■■■■□□□□■■□□■■■	2	ARM(2)	T1

1386	■■■■■■■■■■■■■■■■■■■■■■■■■■■■□□□□□□■■■■■□□□■	3	BGD(2) USA (1)	U
1387	■□□□□□■■■■■■■■□□□□□□□□□□■■■■■■■■□□□□■■■■■■■	2	ARM(2)	T5_RUS1
1388	■■■■■■■■■■■■■■■■■■■■■■□□□■■■□□□□■□■■□■■□■■■	3	BGD(2) USA (1)	EAI1_SOM
1389	□□■■■■■■■■■■■■■■■■■■■■■■■■■■□□□□■□■■■■■□■■■	9	BGD(7) GBR(1) GEO(1)	EAI1_SOM
1390	■■■■■■■■■■■■■■■■■■■■■■□□■■■■□□□□■□■■□■■■■■■	3	BGD(1) DEU(1) USA(1)	EAI5
1391	■■■■■■■■■■■■■■■■■■■■■□□□□□□□□□□□□□■■□■■■■■■	14	BGD(10) A(IT) MYS(2) USA(1)	EAI
1392	■■■■■■■■■■■■■■■■■■■■■■■■■■■■□□□□■□■■■□□□■■■	6	BGD(4) MYS(1) USA (1)	EAI5
1393	■■■■■■■■■■■■■■■■■■■■■■■■■■■■□□□□□■□■■■■□□□□	2	BGD(2)	U
1394	■■■■■■■■■■■■■■■■■□■■■■■■■■■■■■■■□□□□■■□■■■■	4	BGD(3) FXX(1)	X1
1395	■□□■■■■□□□■■■■■■■■■■■■■□■■■■□□□□■□■■■■■■■■■	3	BGD(2) USA (1)	EAI5
1396	■■■■■■■■■■■■■■■■■■■■■■□■□□□□□□□□□□■□□■■■■■■	5	BGD(5)	U
1397	■■■■■■■■■■■■■■■■■■■■■■■□□□■■□□□□■□■■■■■□□□□	8	BGD(8)	EAI5
1398	■□■■■■■■■■■■■■■■■■■■■■□□□□□□□□□□□□■■■■■■■■■	2	BGD(2)	U
1399	□□□■■■■■■■■■■■■■■■■■■■■■■■■■□□□□■□■■■■■□□□□	4	BGD(4)	EAI5
1400	■■■□□□□□□□□□□□□□□□□□□□□□■■■■□□□□■□■■□■■■■■■	7	BGD(7)	EAI5
1401	■■■□□□□■■■■■■■■■■■■■■■□□□□□□□□□□□□■■■■□■■■■	4	BGD(3) GBR(1)	CAS1_DELHI
1402	■□■■■■■■■■■■■■■□□□□□□■□■□□□□□□□□□□□□□□□□□□□	2	BGD(2)	U
1403	■■■■■■■■■■■■■■■■■■■■■■□■■■■□□□□□□□■■□■■■■■■	3	BGD(3)	U
1404	■■■■■■■■■■■■■■■■■■■■■■■□■■■■□□□□■□■■■■■□■■■	3	BGD(3)	EAI1_SOM
1405	■■■□□□□■■■■■■□□□■■■■■■□□□□□□□□□□□□■■■■■■■■■	2	BGD(2)	CAS1_DELHI
1406	■■■■■■■■■■■■■■■■■■■■■■□■■■■■□□□□■□■■□□■■■■■	2	BGD(2)	EAI6_BGD1
1407	■■■■■■■□■□■■■■■■■■■■■■■■■■■■□□□□■□■■■■■■■■■	2	BGD(2)	EAI5
1408	□□□□□□□□□□□□□□□□□□□□□□□□■■■■□□□□■□■■□■■■■■■	2	BGD(1) NLD(1)	EAI5
1409	■■■■■■■■■■■■■■■■■■■■■■□■■■■■□□□□■□■■□■□■■■■	3	BGD(1) USA (2)	EAI6_BGD1
1410	■■■■■■■■■■■■■■■■■■■■■□□□□□□□□□□□□□□□□□□□□□□	9	AUT(1) BGD(1) BRA(3) POL(1) PRT(1) SAU(2)	U
1411	■■■■■■■■■■□□■■■■■■■■■■□■■■■■□□□□■□■■□■■■■■■	2	BGD(2)	EAI6_BGD1
1412	■■■■■■■■■□■■■■■■■■■■■■□■■■■■□□□□■□■■□■□■■■■	3	BGD(3)	EAI6_BGD1
1413	□□■■■■■■■■■■■□□□■■■■■■■■■■■■□□□□■□■■■■■□■■■	2	BGD(2)	EAI1_SOM
1414	■■■■■■■■■■■■■□■■■■■■■■□■■■■■□□□□■□■■□■■■■■■	2	BGD(1) USA (1)	EAI6_BGD1
1415	□□□■■■■■■■■■■□□□■■■■■■■■■■■■□□□□■□■■■■■□■■■	2	BGD(2)	EAI1_SOM
1416	■■■■■■■■■■■■■□□■■■■■■■□■■■■■□□□□■□■■■■■■■■■	2	BGD(1) VNM(1)	EAI6_BGD1
1417	■■■■■■■■■■■■■■■■■■■■■■□■■■■■□□□□■□■■□□□■■■■	4	BGD(4)	EAI6_BGD1
1418	■□■■■■■■■■■■■■■■■■■■■■□■□□□□□□□□□□■■■■■■■■■	4	BGD(4)	U
1419	■■■□□■■■■■■■■■■■■■■■■□□□□□□□□□□□□□■■□■■■■■■	3	BGD(3)	U
1420	■■■■■■■■■■■■■■■■■■■■■■□■■■■■□□□□■□□□□□□■■■■	5	BGD(5)	U
1421	■■■■■■■■■■■■■■■■■■■■■■□■■■■■□□□□□□■■□■■■■■■	2	GBR(1) GEO(1)	U
1422	■■■□□□□■■■■■■■■■■■□□□□□□□□□□□□□□□□□□■■■■■■■	2	BGD(2)	CAS
1423	■□□■■■■■■■■■■■■■□□■■■■■■■■■■□□□□■□■■■■■■■■■	2	BGD(2)	EAI5
1424	■■■■■■■■■■■■■■■■■■□□□□□■□□□□□□□□□□■■□■■■■■■	2	BGD(2)	EAI7_BGD2
1425	■■■■■■■■■■■■■■■■■■■■■■□■■■■■□□□□■□■■□■■□■■■	4	BGD(4)	EAI1_SOM-EAI6
1426	■■■■■■■■■■■■□■■■■■■■■■■■■■■■■■■■□□□□■■■□□■■	2	BGD(1) GBR(1)	T3
1427	■□□■□■■■■□□□□□□□□■■■■■■■■■■■□□□□■□■■■■■■■■■	4	BGD(2) GEO(2)	EAI5
1428	■■■■■■■□□■■■□□□■■■■■■■■■■■■■□□□□■□■■■■■■□■■■	3	BGD(3)	EAI1_SOM
1429	■■■□□□□■■■■■■■■■■■■■■■□■□□□□□□□□□□■■■■■■■■■	3	BGD(3)	U
1430	■■■■□■■■■■■■■■■■■■■■■□□□□□□□□□□□□□■■□■■■■■■	2	BGD(2)	U

1431	□□■■■■■□□□□□□□□□□□□□□□□□■■■■□□□□■□■■■■■□■■■	2	BGD(2)	EAI1_SOM
1432	■■■■■■■■■■■■■□□□■■□■■□□□□□□□□□□□□□□□□□□□□□□	2	BGD(2)	U
1433	■□■■■■■■■■■■□■■■■■■■■■□■■■■■□□□□■□■■■■■■■■■	2	BGD(2)	EAI6_BGD1
1434	■■■■■■□■■■■■■■■■■■■■■■□□□□□□□□□□□□□□□□■■■■■	3	BGD(3)	U
1435	■■■■■■■□□■■■■■■■■■■■■■■■■■■■□□□□■□■■■■■□■■■	4	BGD(4)	EAI1_SOM
1436	■□■■■■■■■■■■■■■■■■■■■■□■■■■■□□□□□□■■■■■■■■■	3	BGD(3)	U
1437	■■■□□□□■■■■■□■■■■■■■■■□□□□□□□□□□□□■■■■■□■■■	2	BGD(2)	CAS1_DELHI
1438	■■■■■■■■■■■■■■■■■□■■■■□■■■■■□□□□■□■■□■■■■■■	3	BGD(2) USA(1)	EAI6_BGD1
1439	■■□■■■■□□□■■■■■□■■■■□■■■■■■■■■■■■■■■■□□□□□	2	BEL(1) ESP(1)	BOV
1440	■■□■■■■■□■■■■■■■■■■■□■■■■■■■■■■■■■■■■□□□□□	8	BEL(8)	BOV
1441	□□□■■■■■□■■■■■■■■■■■■■■■■■■■■■■■■■■■■□□□□□	4	BEL(4)	BOV
1442	■■□□□■■□□□□□■■■■■■■■■■■■■■■■■■■■■■■■■□□□□□	6	BEL(6)	BOV
1443	■■□□□■■■■□■■■■■■■■■■■■■■■■■■■■■■■■■■■□□□□□	2	BEL(2)	BOV
1444	■■■■■■■■■■□□□□□□□□□□□□□□□□□□□□■■□□□□■■■■■■■	3	BEL(3)	T1
1445	■□□□□□■■■■■■■□■■■■■■□□□□■■■■■■■■□□□□■■■■■■■	4	GEO(4)	LAM9
1446	■■■■■■■■■■■■■□■■■■■■□■■■■■■■□□□■□□□□■■■■■■■	5	GEO(5)	H4
1447	■■■■■■■■■■■□□■■■■■■■■■■■■■■■□□□■□□□□■■■■■■■	9	GEO(8) USA (1)	H4
1448	■■■■■■■■■■■□□■■■■■■■■■■■■■■■□□□■□□□□■■■□■■■	3	GEO(3)	H4
1449	■■■■■■■■■■■■■■■■■■■■□■■■■■■■□□□■□□□■■■■■■■■	2	GEO(2)	U
1450	■■■■■■■■■■■■■■■■■■■■□■■■□□□□□□□□□□□□■■■■■■■	2	GEO(2)	U
1451	■■■■■■■■■■■■■■□□□□□□□□□□□□□□□□□□□□□□□□□□□□□	8	GEO(7) RUS(1)	U
1452	■■■■■■■□□■■■■■■■■■■■□■■■■■■■□□□■□□□□■■■■■■■	5	GEO(5)	H4
1453	■■■■■■■■□□□■■■■■■■■■□□■■■■■■■■■■□□■■■■■■■■■	3	GEO(3)	MANU2
1454	■■■■■■■□□□□□□□□□□□□■■□■■■■■■■■■■■□□□□□■■■■■■	2	GEO(2)	U
1455	■■■■■■■□□□■■■■■■■■■■□■■■■■■■■□□□■□□□□■■■■■■■	3	GEO(3)	H4
1456	■■■■■■■■■■■□□■■■■■■■□■■■■■■■□□□■□□□□■■■■■■■	5	GEO(5)	H4
1457	■■■■■■■■■■■■■□■■■■■■■■■■■■■■□□□■□□□□■■■■■■■	15	GEO(15)	H4
1458	■■■■■■□■■■■■□■■■■■■■■■■■■■■■□□□□□□□□■■■■■■■	2	GEO(2)	U
1459	■■■■■■■■■■■■■■■■■□■■■■■■■■■■■■■■■□□□□■■■□□□□	2	GEO(1) USA (1)	X1
1460	■■■■■■■■■■■■■■■■■■□■■■■■■■■■■■■■■□□■■■■■■■■■	3	GEO(2) RUS(1)	MANU2
1461	■■■■■■□■■■■■□■■■■■■■□■■■■■■■□□□■□□□□■■■■■■■	2	GEO(2)	H4
1462	■■■■■■■■■■■■■■■■■■■■■■■■□□□□□□□□□□■■■■■■■■■	2	GEO(1) VNM(1)	U
1463	■■□■■■□□□□□□□□□□□□■■■■■■■■■■■■■■□□□□■■■□■■■	3	GEO(3)	T1_RUS2
1464	■■■■■■■■■□■■■■■■■■■■■■■■□□□□□□□□□□□□■■■■■■■	2	GEO(2)	U
1465	■■■■■■■□□□□□■■■■■■■■■■■■□□□□□□□□□□□■□□□■■■■	4	BEL(2) COD(2)	AFRI_3
1466	■■■■□■■■■■■■■■■■■■■■□□□□■■□□□□■■□□□□■■■■■■■	6	MWI(5) ZWE(1)	LAM11_ZWE
1467	■□□■■■■■■■■■■■■■■■■■■■■■■■■■□□□□■□□□■■■■■■■	2	MWI(2)	CAS
1468	□□□■■■■■■■■■■■■■■■■■□□□□■■□□□□■■□□□□■■□■■■■	8	GBR(1) MWI(7)	LAM11_ZWE
1469	□□□□□■■■■■■■■■■■■□■■□□□□■■■■■■■■□□□□■■■■■■■	2	MWI(2)	X1-LAM9
1470	■■■■■■■■■■■■■■■■■□■■□□□□■■■■■■■■□□□□■■■■■■■	5	MWI(1) USA(2) ZAF(2)	X1-LAM9
1471	■■■■■■■■■■■■□■■■■■■■□□□□■■□□□□■■□□□□■■■■■■■	7	MWI(5) ZMB(2)	LAM11_ZWE
1472	■□□■■■■■■■■□■■■■■■■■■■■■■■■■□□□□■□□□■■■■■■■	3	MWI(3)	CAS
1473	■■■□□□□□□■■■■■■■■■■■■■□□■■□□□□□□■□■■■■■■■■■	4	FXX(1) MWI(3)	MANU1
1474	■■□■■■■■■■■■■■■■■■■■□□■□■■■■■■■■□□□□■■■■■■■	2	MWI(2)	T1
1475	■■■■■■■■■□■■■□■■■■■■■■■■■■■■■■■■□□□□■■■■■■■	4	ARG(1) MWI(2) VEN(1)	T1

1476	■■■□■■■■□□□■■■■■■■■■□□□□■■■■■■□□□□□□□□■■■■■	6	ARG(2) PER(2) USA(2)	U
1477	■■□■■■■■■■■■■■■■■■■□□■■□□■■■□□□□■□■■■■■□□□■	2	PHL(2)	EAI2_MANILLA
1478	■■□■■■■□■■■■■■■■■■■□□■■■■■■■□□□□■□■■■■■■■■■	2	PHL(2)	EAI2_MANILLA
1479	■■□■■■■□□■■■■■■■■■■□□■■■□□□□□□□□□□□■■■■■■■■	7	DEU(1) MYS(2) PHL(2) USA(2)	U
1480	■■■■■■■□□■■■■■■■■□□□□□□□□□□■□□□■□□□□■■■■■■■	4	RUS(4)	H4
1481	■■■■■■■□□■■■■■■■■■■■■■■■■■■■□□□■□□■■■■■■■■■	2	RUS(2)	MANU2
1482	■■■■■■■□■■■■■□□□□□□□□□□□□■■■■■■■□□■■■■■■■■■	3	LVA(1) RUS(2)	MANU2
1483	■■■■■■■■■■■■□□■■■■□■■■■■■■■■■■■■■□□□□■■■■■■■	3	RUS(2) USA (1)	T1
1484	■■■■■■■■■■■■■■■■■■■■■■■■■□□□□□□■□□■■■■■■■■■	2	CUB(1) RUS(1)	MANU2
1485	■■■■■■■■■■■■■□□□□□□□□□□□■■■■■■■■□□■■■■■■■■■	3	RUS(3)	MANU2
1486	□■■□□□□□□□□□□■■■■□■■■■■■■■■■■■■■■□□□□■■■■■■■	2	GUF(1) USA (1)	X3
1487	□□□□□□□□□□□□□□□□□□□□□□□□■■■■■■■■■■■■■■■□■■■	2	MYS(1) USA (1)	U
1488	■□□□□□□□■■■■■■■■■■■■■■■■■■■■□□□□■□□□□□□■■■■	2	MYS(1) USA (1)	U
1489	■■□□■■■■■■■■■■■■■■■□□■■■■■■■□□□□■□■■■■■■□■□	2	MYS(2)	EAI5
1490	■■□■■■■■■■■■■■□■■■■□□■■■■■■■□□□□■□■■■■■■■■■	2	MYS(2)	EAI2_MANILLA
1491	■■■■□□□□□□□□□□□□□□□□□□□□■■■■■■■■□□□□■■■□■■■	4	GBR(1) MYS(3)	T2
1492	■■■■■■■■■■■■□□□□□□□□□□□□□□□□□□□□□□□■□□■□■■■	2	MYS(2)	U
1493	■■■■■■■■■■■■■■■■■■■■□□□□□■■■□□□□■□■■■■■□□□□	2	GBR(1) MYS(1)	EAI5
1494	■■■■■■■■■■■■■■■■■■■■□■■■■■□■■■■■□□□□■■■■■■■	5	BEL(1) IDN(3) MYS(1)	T1
1495	■■■■■■■■■■■■■■■■■■■■■□■■■■■■□□□□■□■■■■■■■■■	2	MYS(1) THA(1)	EAI5
1496	■■■■■■■■■■■■■■■■■■■■■■□■■■□■□□□□■□■■■■■■■■■	2	MYS(2)	EAI6_BGD1
1497	■■■■■■■■■■■■■■■■■■■■■■■■■□■■□□□□■□■■■■□■■■■	2	MYS(1) THA(1)	EAI5
1498	■■■■■■■■■■■■■■■■■■■■■■■■■■□□□□□□□□□□□■■■■■■	6	MYS(2) USA (4)	U
1499	■■□■■■■■■□□□□□□□■■■□□■■■■■■■□□□□■□■■■■■■■■■	3	MYS(2) USA(1)	EAI2_MANILLA
1500	■■□■■■■■■■■■■■■■■■■□□■■■■■■■□□□□□□□□■■■■■■■	2	MYS(2)	U
1501	■■□■■■■■■■■■■■■■■■■□□■■■■■■■□□□□■□■■■■□■■■■	4	MYS(3) PHL(1)	EAI2_MANILLA
1502	■□□■■■■■■■■■■□■■■■■■■■■■■■■□□□□□■□■■□□□■■■■	2	MYS(2)	MANU1
1503	■■■■■■■■■■■■■□□■■■■■■■□■■■■■□□□□■□■■□■□■■■■	2	MYS(2)	EAI6_BGD1
1504	■■■■■■■■■■■■■□□■■■■■■■■■■■■■□□□□■□■■■■□■■■■	2	MYS(2)	EAI5
1505	■■■■■■■■■■■■■□■■■■■■□□□□■■■■■■■■□□□□■■□■■■■	2	MYS(2)	LAM9
1506	■■■■■■■■■■■■■□■■■■■■■■□■■■■■□□□□■□■■□■□■■■■	3	MYS(3)	EAI6_BGD1
1507	■■■■■■■■■■■■■□■■■■■■■■□■■■■■□□□□■□■■■■■■■■■	2	MYS(2)	EAI6_BGD1
1508	■■■■■■■■■■■■■□■■■■■■■■■■■■■■□□□□■□■■■■■■■■■	2	MYS(2)	EAI5
1509	■■□■■■■■■■■■■■■■■■■□□■■■□■□□□□□□□□□□□□□□□□□	3	DEU(1) PHL(2)	U
1510	■■□■■■■■■■■■■■■■■■■□□■■■■■□□□□□□□□■■■■■■■■■	2	PHL(2)	U
1511	■■□■■■■■■■□■■■■■■■■□□■■■■■■■□□□□■□■■■■■■■■■	2	PHL(2)	EAI2_MANILLA
1512	■■□■□■■■■■■■■■■■■■■□□■■■■■■■□□□□■□■■■■■□□□■	3	MYS(3)	EAI2_MANILLA
1513	■■■■■■■■■■□■■■■■■■■■■■□■■■■■□□□□■□■■■■■■■■■	2	MYS(2)	EAI6_BGD1
1514	■■■■□■■■■■■■■■■■■■■■□□■■■■■■■■■■□□□□■■■■■■■	6	MDG(4) USA(2)	U
1515	■□□■■■■■■■■■■■■■■■■■■■■■■■■■□□□□□□■■■■■■■■■	3	MDG(3)	U
1516	■■■■■■■■■□■■■■■■■■■■■■■■■■■■■■■■□□□■■■■■■■■	3	MDG(3)	U
1517	■□□■■■■■■■■■■■■■■■□■■■■■■■■■□□□□■■■■■■■■■■■	6	MDG(6)	U
1518	■□□■■■■■■■■■■■■■■■□■■■■■■■■■□□□□□□■■■■■■■■■	17	MDG(16) SAU(1)	U
1519	□□□□□□□□□□□□□■■■■■■■■■■■■■■■□□□□□□■■■■■■■■■	2	MDG(2)	U
1520	■□□■■■■■■■■■■■■■■■□■□■■■■■■■□□□□□□■■■■■■■■■	3	MDG(3)	U

1521	■■■□□□□□□■■■■■■■□□□□□□□□■■■■□□□□□□■■■■■■■■■	2	MDG(2)	U
1522	■■■■■■■■■■■■□■■■■■■■□□□□■■□□□□■■□□■■■■■■■■■	2	MDG(2)	MANU2
1523	■■■■■■■■■■■■■■■■■■■■□■■□■■■■■■■■□□■■■■■■■■■	2	MDG(2)	MANU2
1524	■■■□□□□□□■■■■■■■■■■■■■□□■■■■□□□□□□■■■■■■■■■	3	MDG(3)	U
1525	□■■■■■■■□□□■□■■■■■■■□□□□■■■■■■■■□□□□■■■■■■■	4	FXX(1) MDG(3)	LAM3
1526	■■■□□□□□□□□□□■■■■■□□■■■■■■■■■■■■□□□□■■■■■■■	2	GUF(2)	T1
1527	■■■■■■■■■■■■■■■■■□■■■■■■■□□□□□□■□□□□■■■■■■■	3	DEU(1) SWE(1) USA (1)	H1-X1
1528	■■■■■■■□□□■■■■■■■■■■□□□□■■■■■■■■□□□□■■■■■■■	2	BRA(1) SWE(1)	LAM9
1529	■■■■■■■■■■■■■■■■■■■■■■■■■■■■■■□■□□□□□□■■■■■	2	AUT(1) ESP(1)	U
1530	■■■■■■■■■■■■■■■■■■■■□□□□■■■■■■■■□□□□■■■□□■■	6	ESP(3) USA (3)	LAM9
1531	■■■■■■■■■■■■■■■■■□■■■■■■■■■■■■■■□□□□□□□■■■■	2	ESP(2)	U
1532	■■■■■■■■■■■■■■■■■□■■■■■■■□■■■■■■□□□□■■■■■■■	2	ESP(1) USA(1)	X1
1533	■■■■■■■■■■■■■■□□■■■■■■■■■■■■■■□■□□□□■■■■■■■	2	AUT(1) ESP(1)	H3
1534	■■■■■■■■■■■■□■□□□■■■□□□□■■■■□■■■□□□□■■■□■■■	3	ESP(1) USA (2)	LAM5-LAM6
1535	■■■■■■■■■■□■■■■■■■■■□□□□■■■■■■■■□□□□■■■■■■■	6	ARG(1) BEL(1) BRA(2) ESP(1) USA(1)	LAM9
1536	■■■■■■■■□□■■■■■■■■■■□□□□■■■■■■■■□□□□■■■■■■■	2	BRA(1) ESP(1)	S
1537	■■■■■■■■□□□■□■■■■■■■□□□□■■■■■■■■□□□□■■■□■■■	2	ESP(1) ITAS(1)	LAM3
1538	■■■■■■■□□□□■□■■■■■■■□□□□□□□□□□□■□□□□■■■■■■■	4	BEL(1) ESP(2) NLD(1)	H3
1539	■■■■■■□■■■■■■■■■■■■■■■■■■■■■■■□■□□□□■■■■■■■	4	ESP(3) MYS(1)	H3
1540	□■■■■■■■■□■■■■■■■■■■□□□□■■■■■■■■□□□□■■■■■■■	2	ESP(1) IDN(1)	LAM9
1541	□■■■■■■■□□■■■■■■■■■■□□□□■■■■■■■■□□□□■■■■■■■	2	ESP(2)	LAM9-S
1542	□□■■■■■■■■■■■■■■■□■■■■■■■■■■■■■■□□□□■■□□□□■	2	ESP(1) USA (1)	X2
1543	□□□■■■■■□□□■■■□□□□□□□□□□□□□□□□□□□□□□□□□■■■■	2	ESP(2)	U
1544	■■■■■■■■■■■■■■■■■■■■■■■■■■■■■■■■□□□□■□■□■■■	3	BEL(1) FXX(1) ITA(1)	T2
1545	■■□□□□□□■■■■■■■■□■■■□□□□■■■■■■■■□□□□■■■■■■■	6	BEL(5) BRA(1)	LAM9
1546	□□□■■■■■■■■■■■■■■■■■■■■■■■■■□□□□■□■■□□□□□■■	4	NZL(2) USA (2)	EAI5
1547	■■■■■■■■■■■■□■□■■■■■■■■■■■■■■■■□□□□■■■■■■■	4	DNK(1) EGY(1) IDN(1) POL(1)	T3
1548	■■■■■■■■■■■■□□□□■■■□□□□□□□□□□□□□□□□□□■■■■□□	2	BEL(1) FXX(1)	U
1549	■■■■■■■□■■■■■■■■■■■■□□□□■■□□□□■■□□□□■■■□■■■	4	BDI(1) BEL(3)	LAM11_ZWE
1550	□■■■■■■■■■■■■■■■■■■■■■■■■■■■■□■■□□□□■■■■■□□	2	AUT(2)	T1
1551	■■■□□□□□■■■■■■■■■■■■■■□□□□□□□□□□□□■■■■■■■■■	3	AUT(1) SWE(1) USA(1)	CAS
1552	■■■■■■■■■■■■■■■■■■■■■■■■■□□□□□□■□□□□■■□□■■■	4	AUT(4)	H1
1553	■■■■■■■□■■■■■■■■■■■□□□□□□□■■■■■■□□□□■■■■■■■	2	AUT(2)	T1
1554	■□□□□□□■□■■■■■■■■■■■□□□□■■■■■■■■□□□□■■■□■■■	7	AUT(6) DEU(1)	LAM4
1555	■■■□□□□□□□□□□□□□□■■□□□□□□■■■■■■■□□□□□■■■■■■	2	POL(2)	U
1556	■■■■■■■□■■■■■■■■■■■■■■■■■■■■■□■■□□□□■■■■■■■	2	AUT(1) POL(1)	T1
1557	■■■■■■■■■■■□■■■■■■■■■■■■■□□□□□□■□□□□■■■■■■■	8	POL(8)	H1
1558	■■■■■■■■■■■■■■■■■■■■■■■■■□■■■□■■□□□□■■■■■■■	11	AUT(1) DEU(1) POL(9)	T1
1559	■■■■■■■■■■■■■■■■■■■□□□□□□□□□□□□□□□□□□□■■■■■	5	MTQ(1) POL(3) USA (1)	U
1560	■■■□□□□■■■■■■■■■■■■■■■■■■■■■■■■□□□□■■■■■■■	2	FXX(1) POL(1)	T1
1561	■■■■■■■■■■■■■■■■■■■■■■■■■□□□□□□■□□□□□■■■■■■	2	POL(2)	U
1562	■■■■■■■■■■■■■■■■■■□■■■■■■■■■□□□□□□□□□□■■■■■	3	BEL(1) FIN(1) POL(1)	U
1563	■■■■■■■■■■■■■■■■■■■■□□■■■■□■■■■■□□□□■■■■■■■	2	NLD(1) POL(1)	T1
1564	■■■■■■■■■■■■■■■■■□■■■□■■■■■■■■■■□□□□■□■■■■■	4	POL(2) RUS(2)	X1
1565	■■■■■■■■■■■■■□□□□□□□□■■■■■■■■■■■□□□□■■■■■■■	5	AUT(3) POL(1) SWE(1)	T1

1566	■■■■□□■■■■■■■■■■■■■■■■■■■■■■■■■■□□□□■■■■■■■	2	CUB(1) POL(1)	T1
1567	■■■■■■■■■■■■■■■■■■■■■□□□■■■■□■■■□□□□■■■■■■■	4	AUT(1) BRA(2) FXX(1)	T1
1568	■■■■■■■□□■■■■■■■■■□■■■■■■■■■□□□■□□□□■■■■■■■	2	RUS(1) USA(1)	H4
1569	■■■□□□□□□□□□□■■■■■□□□□□□□□□□□□□■□□□□■■■□□□□	2	USA(2)	H3
1570	■■■■■■■■■■■■■□□□□□□□□□□□□□□□□□□□□□□□□□□□□□□	3	IDN(1) MYS(1) USA(1)	U
1571	■■■■■■■■■■■■■■■■□■■■■■■■■□□■□□□□■□■■■■■■■■■	2	NLD(1) USA(1)	EAI4_VNM
1572	■■□■■■■■■■■■■■■■■■■■■■■■■■■■■■■□□□□■■■□■■□	2	USA(2)	T2
1573	■■■■■■■■■■■■■■■■■■□□■■■■■■■■□□□□■□■■□■■■■■■	3	USA(2) VNM(1)	EAI5
1574	■■■■■□□□□□□□□□□□□□□□□□□□■■■■■■■■□□□□■■■■■■■	4	MEX(1) USA(3)	T1
1575	■■■■■■■■■■■■■■■■■■□■■■□□■■■■■■■■□□□□■□□□□□□	2	USA(2)	T4
1576	■■■■■■■■■■■■■■■■□□□□□■■□□■■■□■■■□□□□■■■■■■■	2	USA(2)	T1
1577	■■■■■■■■□□■■■■■■■□□□□□□□□■■■■■■■□□□□■■■■■■■	3	ARG(1) DEU(1) FXX(1)	S
1578	■■■■■□■■■■■■■■■■■■■■■■■■■■■■■■■■□□□□■■■□■■■	2	AUT(1) DEU(1)	T2
1579	■■□■■■■■■■■■■■■■■■■■■■■■■■■■■■□□□□□■■■■■■■	2	DEU(1) POL(1)	U
1580	■■■■■■■■■■■■■■■■■■■■■■□□■■■■■■■■□□□□■■■■■■■	3	BRA(1) DEU(1) GEO(1)	T1
1581	■□■■■■■■■■□□■■■■■■■■■■■■■■■■□□□■□□□□■■■■■■■	2	DEU(1) USA (1)	H4
1582	■■■■■■■■■■■■■■■■■■□■■■■■■■■■■■■■□□□□■■■□□□□	2	DEU(1) GLP(1)	T1
1583	■■■■■■■■■■■■■■■■■■■■■■■■■■■■■■■■□□□□■■■■□□□	3	BRA(1) DEU(1) FXX(1)	T4
1584	■■■■□■■■■■■■■■■■■■■■□□■■□□□■■■■■□□□□■■■■■■■	2	DEU(1) USA(1)	T1
1585	■■■■■■■■■■■■■■■■■■■■■■■□■□□□□■□■□□□□■■■■■■■	2	DEU(1) USA (1)	H3
1586	□□■■■■■■■■■■■■■■■■■■■■■□■□□□□□□■□□□□■■■■■■■	12	AUT(1) CZE(1) DEU(9) USA (1)	H1
1587	■■□□■■■■□□■■■■■■■■■■■■■■□□□■■■■■□□□□■■■■■■■	2	AUT(1) DEU(1)	S
1588	□□□□■■■■■■■□□■■■■■■■□□□□■■■■■■■■□□□□■■■■■■■	2	DEU(2)	LAM9
1589	■■■■■■■■□□□□□□■■■■■□□□□□■□□■■■■■□□□□■■■■■■■	2	DEU(1) NLD(1)	LAM7_TUR
1590	■■■□□□□■■■■■■■■■■■□■■■□□□□□□□□□□□□■■■■■■■■■	2	DEU(1) PAK(1)	CAS1_DELHI
1591	■■■□□□□□□□■■■■■■■■■■■■□□□□□□□□□□□□■■□■■■■■■	2	DEU(1) IND(1)	CAS2
1592	■■■■■■■□□□□□■■■■■■■■□□□□□■□□□□□□□□□□□□□□■■■	2	DEU(1) FXX(1)	U
1593	■■■■□□□□□□□□□□□□□□□□□□□□□□□□□□□■□□□□■■■■■■■	2	DEU(1) POL(1)	H3
1594	■■■■■■■■■■□□□□□□□□□□□□□□■■■■■■■■□□□□■■■■■■■	2	POL(2)	T1
1595	■■□■■■■□□□■■■■□□■■■■□■■■■■■■■■■■■■■■■■□□□□□	2	DEU(2)	BOV
1596	□■□□□□□□□□□□□□□□■■■■■■■■■■■□■■■■■■■■□■□□□□□	2	DEU(2)	CAP
1597	■■■□□□■■■■■■■■■■■■■■■■■■■■■■■■■■□□□□■■■■■■■	5	IDN(1) RUS(2) USA (2)	T1
1598	□■□■■□□■□■■■■■■□■■■■■■■■■■■■■■■■■□■□■■□□□□□	2	DEU(2)	BOVIS1
1599	□□□□□□□□□□□□□□□□■■■■■■■■■■■□■■■■■■■■■■□□□□□	3	DEU(1)	BOV
1600	□■□□□□□□□□□□□□□□■■■■■■■■□□□□□□□□□□■■■■□□□□□	3	DEU(3)	CAP
1601	■■□■■■■■□■■■■■■□■■■■□■■■■■■■■■■■■■■■■□□□□□□	3	DEU(2)	BOV
1602	■■□■■■■□□□□□□■□□■■■■■■■■■■■■■■■■■■■■■■□□□□□	2	DEU(2)	BOV
1603	■■□■■□■■□■■■■■■□■■■■■■■■■■■■■□■■■■■■■■□□□□□	2	DEU(1) NLD(1)	BOVIS1
1604	□■□□□□□□□□□□□□□□■■□■■■■■■■■□■■■■■■■■■■□□□□□	2	AUT(1) DEU(1)	CAP
1605	■□□□□■■■□■■■■■■□■■■■□■■■■■■■■■■■■■■■■■□□□□□	2	DEU(2)	BOV
1606	■■□□□□□■■■■■■■■■■■■■■■□□□□□□□□□□□□□□■■■■■■■	2	AUS(1) USA(1)	CAS
1607	■■□□□□□□■■■■■■■■■■■■□□□□■■□□□□■■□□□□■■■■■■■	3	USA(2) ZWE(1)	LAM11_ZWE
1608	■■■■■■■■■■□□□□■■■■■■□□□□■■■■■■■■□□□□■■■□□□□	2	USA(2)	LAM9
1609	■■■■■■■■■■■□□■■■■■■■■■■■■■■■□□□□■□■■■■■■■■■	3	USA(2) VNM(1)	EAI5
1610	■■■■■□□■■■■■■■■■■■■■□□□□■■■■□■■■□□□□■■■■■■■	2	USA(2)	LAM6

1611	■■■■■■■■□■■■■■■■■■■■■■■■■■■■■■□■□□□□■■■■■■■	3	FXX(1) USA(2)	H3
1612	□□□■■■■■■■□■■■■■■■■■■■□■■■■□□□□□□□■■□■■■■■■	3	BGD(2) USA(1)	U
1613	□□■■■■■■■■■■■■■■■■■■■■■■■■■■■■■■□□□□■■■□■■■	2	AUT(1) USA(1)	T2
1614	■■■■■■■■■■■■■■■■■□■■□□□□■■■■■■■■□□□□■■■□□□□	2	USA(2)	X1-LAM9
1615	■■■■■■■■■■□□□□■■■■■■■■■■■■■■■■■■□□□□■■■□■■■	3	USA(3)	T2
1616	□□□□□□□■■■■■■■■■■■■■■■□□□□□□□□□□□□■■■■■■■■■	2	NLD(1) USA(1)	CAS
1617	■■■□□□□□□□□□□■■■■□■■■■■■■■□■■■■■□□□□■■■■■■■	2	ESP(1) USA(1)	X3
1618	■□■■■■■■■■■■■■■■■■□□□□□□□□□□□□□□□□□□□□□□□■■	3	USA(2) VNM(1)	ZERO
1619	□□□□□□□□□□□□□□□□■□■■■■■■■■■■■■■■□□□□■■■□■■■	2	USA(2)	X1
1620	■■■■■■■■■■■■■■■■■■■■□□□□■□□□■■■■□□□□■■■■■■■	4	BEL(1) ESP(1) USA(2)	LAM9
1621	■■■□■■■■■■■■□■■■■■■■■■■■■■■■■■■■□□□□■■■□■■■	2	ESP(1) USA(1)	T2-T3
1622	■■■■■□□□■■■■■■■■■■■■■■■■■■■■■■■■□□□□■■■□■■■	2	AUT(1) USA(1)	T2
1623	■■■■■■■■■■■■■■■■■■■■□■■■■■■■■■■■□□□□■■■□□□□	2	CHN(1) USA(1)	T1
1624	■■■■■■■■□□□■■■■■■■■■■□□□□■■■■□■■■□□□□■■■■■■■	2	USA(2)	LAM3-LAM6
1625	■■□■■□■□□□□□■■■□■■■■■■■■■■■■■■□□□□□□■■□□□□□	2	USA(2)	BOVIS2
1626	■■■■■■■■■■■■■■■■■■■■■■■■■■□■■■■■□□□□■■■■■■■	3	USA(3)	T1
1627	■■■■■■■■■□□■□■■■■■■■■■■■■■■■■■■■□□□□■■■□■■■	2	USA(1) ZAF(1)	T2-T3
1628	□□□■■■■■■■■■□■■■■■■■■■■■■■■■□□□□■□■■■■■■■■■	4	USA(4)	EAI5
1629	■■■■■■■■■■■■■■■■■■■■■■■■■□□□□□□■□□□□■■■■□■■	3	MUS(1) USA(2)	H1
1630	■■■■■■■■■■■■■■■■■■■■□□□□□□■■■■■■□□□□■■■■■■■	2	NLD(1) USA(1)	T1
1631	■■■■□■■■■■■■■■■■■■■■■■■■■■■■■■■□□□□■□■■■■■	2	USA(2)	T1
1632	■□■■■■■■■■■■■■■■■■■■■■■■■■□□□□□□□□□□□■■■■■■	2	AUT(1) USA(1)	U
1633	■■□□■■■■■■□□□□□□■■■■□□□□■■■■■■■■□□□□■■□■■■■	2	USA(2)	LAM9
1634	■■■■■■■■■■■■■■■■■■■■■■■■■■■■■■□■□□■■■■■■■■■	3	IDN(1) USA(1) USA (1)	MANU2
1635	□■□□□□□□□□□□□□□□■■■■■■■■■■■□■■■■□□■■■■□□□□□	2	DEU(2)	MANU2
1636	□■□□□□□□□□□□□□□□■■■■■■■■■□□□□□■■■■□■■■□□□□□	2	DEU(2)	CAP
1637	■■□■■■■■□■■■■■■□■■■■■■■■■■■■□■■■■■■■■■□□□□□	2	DEU(1) NLD(1)	BOVIS1
1638	■□■■■■■■■■■■■■□■■■■■■■■■■■■■■■■■□□■■■■■■■■■	2	CHN(2)	MANU2
1639	■■■■□□□■■■■■■■■■■■■■□□□□■■■■■■■■□□□□■■■■■■■	2	CIV(1) FXX(1)	LAM9
1640	■■■■■■■■■■■■■■■■■■■■■■■■■■■■■■□■□□□□■■■■□□□	2	FXX(1) NLD(1)	H3
1641	■■■■■■■■■■■■□■■■■■□□■■■■■■■■■■■■□□□□■■■■■■■	2	FXX(1) PAK(1)	T3
1642	■■■□□□□□□□□□□□■■■■■■■■■■■■■■■■■■□□□□■■■■■■■	2	FXX(2)	T1
1643	■■■■■■■■■■■■■■■■■■■□□□■□□■■■□■■■□□□□■■■■■■■	3	FXX(1) USA(2)	T1
1644	■■■■■■■■■■■■■■■■■■■□□□□□■□□□□□□■□□□□■■■■■■■	3	CZE(1) FXX(1) USA (1	H1
1645	□■■■■■■■■■■■■■□■■■■■■■■■■■■■■■■■□□□□■■■■■■■	2	FXX(1) GLP(1)	T1
1646	■■■■■■■■■■■■□■■■■■■■□□□□■■■□□□□■□□□□■■■■■■■	4	FXX(4)	H3-LAM5
1647	■■■■■■■■■■■■■■■■■■■□□■■■■■■■■■■■□□□□■■■□■■■	3	AUT(1) DEU(1) FXX(1)	H37Rv/T2
1648	■□□□□□□□□□□□□□■■■■■■■■■■■■■■□□□□■□■■■■■■■■■	3	COM(2) NLD(1)	EAI5
1649	■■■■□■■■■■■□■■■■■■■■■■■■■■■■□□□□■□■■■■■□■■■	2	MOZ(1) ZAF(1)	EAI1_SOM
1650	■■■□■□□□□□□□□□□□□□■■□□□□□□□□□□□□□□□□□■■■■■■■	2	MOZ(2)	U
1651	□□□□□□□□□□□□□□□□□□□□□□□□□□□□□□□□□□■■■■□■■□■	2	IDN(1) IND(1)	BEIJING
1652	■□□■■■■■■■■■■■■■■■■■■■■■■□□□□□□■□□□□■■■■■■■	2	IDN(1) VNM(1)	H1
1653	■■■□■■□□□□□□□□□□□□□□□□□□□□□□□□□□□□□□□□■■■■■	2	IDN(2)	U
1654	■■■■■■■■■■■□■■■■□□□□□■■■■■■■■□□□□■□■■■■□■■■■	2	IDN(2)	EAI5
1655	■■■■■■■■■■■■□■□□■■■■■■■■■■■■■■■■□□□□■■■■■■■	14	IDN(14)	T3

1656	■■■■■■■■■■■■■■□■■■■■■■■■□□□□□□□□□□□□□□□□□□□	3	EGY(1) IDN(2)	U
1657	■■■■■■■■■■■■■■■■■■□■■■■■■■■■□□□□□□□□□□■■■■■■	2	IDN(1) POL(1)	U
1658	■■■■■■■■■■■■■■■■■■■■■□□■■□□□□□□■□□□□■■■■■■■	2	AUT(1) IDN(1)	H1
1659	■■■■■■■■■■■■□□□□■■■■■□□□□□□□□□□□□□□□□□□□□□	4	BRA(4)	U
1660	■■■■■■■■■■■■■■■■■■■■□□□□□□□□□□■■□□□□■■■□■■■	3	ARG(1) BRA(2)	T2
1661	■■□■■■■■■■■■□■■■■■■■□□□□■■■■■■■■□□□□■■□■■■■	4	BRA(2) VEN(2)	LAM2
1662	■■□□□□□□□□□□□□□□□□□□□□□□□□□□□□■■□□□□■■■□■■■	2	BRA(2)	T2
1663	■■■■■■■■■■■■■■■■■■■■□□□□■■■■□■□■□□□□■■■■■■■	2	BRA(2)	H3-LAM6
1664	■■■■■■■■■■■■■■■■■■■■□□□□□□□■□■■■□□□□■■■□■■■	3	BRA(3)	T2
1665	□■■■■■■■■■□□□□□□□□□□□□□□□□□□□□□□□□□□■□■■■	2	BRA(2)	U
1666	■■□■■□■□□□□□■■□□■■■■■■■■■■■■■■■■■■■□■■□□□□□	3	BRA(3)	BOV
1667	■■□□□■■■□■■■■■■□■■■■■■■■■■■■■■■■■■■■□■□□□□□	6	ARG(2) BRA(3) MEX(1)	BOVIS1
1668	■■□■■■■■□■■■■■□□■■■■□■■■■■■■■■■■■■■□□■□□□□□	6	BRA(6)	BOV
1669	■■□■■■■■□■■■■■□□■■■■□■■■■■■■■■■■■■■□■■□□□□□	4	BRA(4)	BOV
1670	□□□■■□■□□□□□■■■□■■■■■■■■■■■■■■■■■■■■■■□□□□□	16	ARG(6) BRA(9) MEX(1)	BOV
1671	□■■■■■■■■■■■■■□■■■■■□□□□■■■■■■■■□□□□■■■■■■■	2	BRA(1) FXX(1)	LAM9
1672	■■■■■■■■□□□■■■■■■■■■■■■■■□■■■■■■□□□□■■■■■■■	2	MDG(2)	T1
1673	■■□□□■■□□□■■■■■□■■■■■■■■■■■■■■■■■■■■■■□□□□□	8	MDG(7) NLD(1)	BOV
1674	□□□□□□□□□□□□□□□□□□□□□□□□□□□□□□□□□□■■■■■■■□■	3	MDG(1) REU(1) USA(1)	BEIJING
1675	■■■□□□□■■□■■■■□■■■■□□□□□□□□□□□□□□□□■■■■■■■■	3	FXX(1) MDG(2)	CAS1_KILI
1676	■□□■■■■■■■■■■■■■■■□■■■■■■■■■■□□□■□■■□□□■■■■	2	MDG(2)	MANU1
1677	■■■□□□□■■□■■■■■■■■■□□□□□□□□□■□□□□□■■■■■■■■■	2	MDG(2)	U
1678	■■■■■■■■■■■■■■■■□□□■■■■■■■■■■■□■□□□□■■■■■■■	2	MDG(2)	H3
1679	■■■■■■■■■■■■■■□■■■■■■□■■■■■■■■■■□□□□■■■■■■■	4	DEU(1) MDG(3)	T1
1680	■□□■■■■□■■■■■■■■■■■■■■■■■■■■□□□□■□■■□□□■■■■	2	GBR(1) MDG(1)	EAI3_IND
1681	□■■■■■■■■■■■■■■■■■■■■■■■■□□□□□□■□□□□■■■■■■■	2	AUT(1) BRA(1)	H1
1682	■■□■■□□■□■■■■■■□■■■■□■■■■■■■■■■■■■■■■■□□□□□	2	BRA(1) NLD(1)	BOVIS1
1683	■■□■■■■■■■■■■■■■■■■■■■■■■■■■■□■■□□□□■■■■■■■	3	BRA(1) RUS(1) USA (1)	T1
1684	■■■■■■■■□□□□□□□□□□□□□□□□□□□□□□□□□□□□□□□□□□□	2	BRA(1) FXX(1)	U
1685	■■■■■■■■□□□□■■■■■■■■□□□□■■■■■■■■□□□□■■■■■■■	2	BRA(1) ZAF(1)	LAM9
1686	■■■■■■■■■■■■■□□□□□□□□□□□■□□□□□□■□□□□■■■■■■■	2	AUT(1) BRA(1)	H1
1687	■■■■■■■■■■■■■■□■■■■■■■■■■■□■■■■■■□□□□■■■■■■■	2	BRA(1) USA (1)	T1
1688	■■■■■■■■■■■■■■■■■■■□□□□□□■■■■■■■□□□□■■■■■■■	4	BRA(1) CZE(1) ITA(1) USA(1)	T1
1689	■■■■■■■■■■■■■■■■■■■■□□□□■■■■■□□□□□□□□□□□■■■	2	BRA(1) MTQ(1)	U
1690	■■■■■■■■■■■■■■■■■■■■■■■■■■■■■■■■□□■□■■■■■■■	2	BRA(1) USA (1)	MANU2
1691	■■□■■■■■■■■■□■□■■■■■□□□□■■■■■■■■□□□□■■■■■■■	17	MEX(1) PRT(2) VEN(14)	LAM2
1692	■□□□□■■■■□□■□■■■■□■■■■■■■■■■■■■■□□□□■■■□■■■	4	VEN(4)	X1
1693	■■■□■■■■■■■■□■■■■■■■□□□□■■■■■■■■□□□□■■■■■■■	3	FXX(1) VEN(2)	LAM5
1694	□■□■■■■■■■■■□■■■■■■■□□□□■■■■■■■■□□□□■■■■■■■	2	USA(1) VEN(1)	LAM5
1695	■■□■■■■■■■■■□■■■■■■■□□□□■■■■■■■■□□□□■■■■■■□	2	BRA(1) VEN(1)	LAM2
1696	■■■□□□■■■■■■□■■■■■■■□□□□■■■■■■■■□□□□■■■■■■■	5	VEN(5)	LAM5
1697	■■■■■■■□■■■■■■□■■■■■□□□□■■■■■■■■□□□□■■□■■■■	2	FXX(1) VEN(1)	LAM9
1698	■■■■■■■■■■■■□■□■■■■■□□□□■■■■■□□□□□□□□□□□■■■	3	VEN(3)	U
1699	■■■■■■■■■□■■■□□■■■■■□□□□■■■■■■■■□□□□■■□■■■■	2	VEN(2)	LAM9
1700	■■■■□■□■■■■■■■■■■■■■■■■■■■□■■■■■□□□□■■■■■■■	3	VEN(3)	T1

1701	■■■■■■■■■□■■□□■■■■■■□□□□■■■■■■■■□□□□■■■■■■■	3	VEN(3)	LAM9
1702	□□□□■■■■■■■■□■■■■■■■□□□□■■■■■■■■□□□□■■■■■■■	3	VEN(3)	LAM5
1703	■■□■■■■■■■■■□■■■■■■■□□□□■■■■□■■■□□□□■■■■■■■	2	VEN(2)	LAM2
1704	■□□■■■■■■■■■□■□■■■■■□□□□■■■■■■■■□□□□■■■■■■■	2	VEN(2)	LAM5
1705	■■■■■■■■■■■■□□□□■■■■■■■■■■■■□□■■□□□□■■■■■■■	2	VEN(2)	T1
1706	■■■□□■■■■■■■■■■■■■■■□□□□■■■■■■■■□□□□■■■■■■■	3	CZE(1) USA (1) VEN(1)	LAM9
1707	■■□■■■■■■■■■■■■■■■■□□□□□□□□■■■■■□□□□■■■■■■■	2	VEN(2)	T1
1708	■■■■■■■■□■■■■■■■■■■■□□□□■■■■■■■■□□□□■■■■■■■	2	LVA(1) VEN(1)	LAM9
1709	■■■■■■■■■■■■□■■■■■■■□□□□■■□■■■■■□□□□■■■■■■■	2	USA (1) VEN(1)	LAM5-LAM8
1710	■■■■■■■■■■■■□■■■■■■■□□□□■■■■■■■■□□□□■■■□■■■	3	BRA(1) NLD(1) VEN(1)	LAM4
1711	■■□■■■■■■□■■□■■■■■■■□□□□■■■■■■■■□□□□■■■■■■■	2	VEN(2)	LAM2
1712	■■□■■■■■■□■■□□■■■■■■□□□□■■■■■■■■□□□□■■■■■■■	2	USA(1) VEN(1)	LAM1
1713	■■□■■□□■■■■■■■■■■■■■□□□□■■■■■■■■□□□□■■■■■■■	3	VEN(3)	LAM1
1714	■■■■■■■■■■■■■□□□□□■■□□□□■■■■■■■■□□□□■■■■■■■	2	VEN(2)	LAM9
1715	■□□□■■■■■■■■■■■■■■■■□□□□■■■■■■■■□□□□■■■□■■■	3	ITA(1) VEN(2)	LAM4
1716	■■■■■□■■■■■■□■■■■■■■□■■■■□■■□■■■□□□□■■■■■■■	2	VEN(2)	T3
1717	■■■■■■■■■■■■■■■■■□■■■■■■■■■■□□■■□□□□■■■■■■■	2	AUS(1) VEN(1)	X1
1718	■■□■■■■■■■■■■■■■■□■■□□□□■■■■■■■■□□□□■■■■■■■	3	VEN(3)	X1-LAM9
1719	■■□■■■■■■□■■□■■■■■■■□□□□■■■■■■■■□□□□■■□■■■■	2	VEN(2)	LAM2
1720	■■■■■■■■■■■■■■□■■■■■■■■■■□□■□□□□■□■■■■■■■■■	2	DNK(2)	EAI4_VNM
1721	■■■■■■■■■■■■■□□■■□■■■■■■■■■■■■■■□□□□■■■■■■■	3	DNK(3)	X1
1722	■■□■■□■□□□□□■■■□■■■■□■■■■■■■■■■■■■■□■□□□□□	2	GBR(1) USA (1)	BOVIS2
1723	■■□■■□■□□□□□■■■□■■■■■■■■□□□□□□□■■■■■■■□□□□□	2	GBR(2)	BOVIS2
1724	■□□□■■■□■■■■■■■□■■□□□□■■■■■■■□□□□■□□■■■■■■■	2	GBR(2)	U
1725	■■■□□■■■■■■■■■■■■■■■■■□■■■■■□□□□■□■■■■■■■■■	2	MYS(2)	EAI6_BGD1
1726	■■■■■■■■■■□■■■■■■■■■■■■■■■■■□□□□■□■■□■■■■■■	2	DNK(1) USA (1)	EAI5
1727	■■■■■■■■□□■■■■■■■■■■■□□□□■■■■■■■□□□□■■■■■■■	2	DNK(1) GRL(1)	S
1728	■■■■■■■■□□■■■■■■■■■■□□□□□■■■■■■■□□□□■■■■■■■	2	DNK(2)	S
1729	■■■□□□□□□□□□□□□□□□□□□□□□■□□□□■■■■■■■■■■■■■■	2	DNK(1) ETH(1)	U
1730	■■□■■■■■□■■■■■■□■■■■■■■■■■■■■■■■□□■■■■□□□□□	3	DEU(1) DNK(2)	BOVIS1
1731	■□■■■■■■■■■■■■■■■■■■■■■■■□□■□□□□■□■■■■■■■■■	2	DNK(2)	EAI4_VNM
1732	■□■■■■■■■■■■■■□■■■■■■■■■■■■■□□□□■□■■■■■□■■■	4	DNK(4)	EAI1_SOM
1733	□■■■■■■■□■■■■■■■□□■■■□■■■■■■■■■■□□□□■■■■■■■	2	DNK(1) NLD(1)	T1
1734	□□□■■■■■■■■■■■□■■■■■■■■■■■■■□□□□■□■■■■■□■■■	2	DNK(2)	EAI1_SOM
1735	■■■■■■■■■■■■■■■■■■■■■■■■■□□□■■□■□□□□■■■■■■■	2	ITA(2)	H3
1736	■■■■■■■■■■■■■■■■■■■■■□□□□□□□□□□□□□□□□■■■■■■	2	ITA(1) USA (1)	U
1737	■■■■■■■■■■■■■■□□□□□□□□□■■■■■■■■■□□□□■■■■■■■	11	ITA(9) USA(1) ZAF(1)	T1
1738	■■■■■■■■■■■■■■□□□□□□□■■□□■■■■■■■□□□□■■■■■■■	5	ITA(5)	T1
1739	■□■■■■■■■■■■■■■■■■□■■■■■■■■■■■■■□□□□■■■□□□□	2	GUF(1) ITA(1)	T1
1740	■□□■■□■■■■■■■■■■■■■■■■■■■□□□□□□□□□□□□□□■■■■	2	ITA(1) USA(1)	U
1741	■■□□□□□□□□□□□□□□□□□□□■■■■■■■□□□□■□■■■■■■■■■	2	ITA(1) USA(1)	EAI5
1742	■■■■■■■□■■■■■■■■■■■■■■■■■□□□□□□■□□□□■■■■■■■	2	FXX(1) ITA(1)	H1
1743	■■■■■□■■■■■■■■■■■■■■■■■■■■■■■■□■□□□□■■□□■■■	4	BEL(2) ITA(1) NLD(1)	H3
1744	■□■■■■■■■■■■■■■■■□■■■■■■□□□□□□□□□□□□□□□□□□□	2	ITA(2)	U
1745	■■■■■■□■■■■■□■■■■■■■■■■■■■■■■■■■□□□□■■■■■■■	3	CZE(1) ITA(1) USA(1)	T3

1746	■□□■■■■■□□■■■■■■■■■■■□■■■■■■■■■■□□□□■■■■■■■	3	BEL(1) ITA(1) POL(1)	S
1747	■■■□□■■■■■■■■■■■■■■■■■□■■■■■■■■■□□□□■■■■■■■	2	DEU(1) USA(1)	T5
1748	■■■■■■■■■■■■■□■■■■■■■■■■■■■■■□□■□□□□■■■■■■■	5	DEU(5)	H3
1749	■■■■■■■■■■■■■■■■■■■■■□■■■□□■■■□■□□□□■■■■■■■	2	DEU(2)	H3
1750	■■■■■■■■■■■■■■□■■■■■□□□□■■■■■■■■□□□□■■■□■■■	3	ITA(1) PRT(2)	LAM4
1751	■■■□□□□□□□□□■■□■■□■■■■■■■■■■■■■■□□□□■■■■■■■	3	AUS(1) PRT(2)	X3
1752	■■□■■■■■■■■■■■■■■■■■□□□□■■■■■■■□□□□□■■■■■■■	3	PRT(2) USA (1)	U
1753	■■■□□□□□□□□□□■■■■■■■■■■■■■■■■■■■□□□□■■■■■■■	2	FXX(1) PRT(1)	T1
1754	■■■■■■■■■■■■■■□■■■■■■■■■■■■■■■■■□□□□■■□□□□■	5	PRT(5)	T1
1755	■■□■■■■■■■■■■■■■■■■■□□□□■■■■□■■■□□□□■■■■■■■	4	PRT(2) USA (2)	LAM1
1756	■■■□□□□□□□□□□■□■■□■■■■■■■■■■■■■■□□□□■■■■■■■	2	PRT(1) USA (1)	X3
1757	■■■■□■■■■■■■■■■■■■■■□□□□□□□□□□□□□□□□□□□□□□□	2	PRT(2)	U
1758	■■■■■■■■■■■■■■■■■■■■□□□□■■■■□■■□□□□□■■■■■■■	2	BRA(1) PRT(1)	U
1759	■■■■■■□■□□□■■■■■■■■■□□□□■■□■■■■■□□□□■■■■■■■	3	PRT(3)	LAM8
1760	■□□■■■■■□□□■■■■■■■■■□□□□■■■■■■■■□□□□■■■■■■■	2	DEU(1) PRT(1)	LAM3
1761	■■□■■■■■■■■■■■□■■■■■■■■■■■■■■■■■□□□□■■■■■■■	3	BEL(1) BRA(1) GUF(1)	T1
1762	■□□■■■■■■■■■■■■■■■■■□□■■■■■■□□□□■□■■□□□■■■■	2	GUF(1) MTQ(1)	EAI3_IND
1763	■□□□■■■■■■■■■■■■■■■■■■■■■□□□■■□■□□□□■■■□■■■	2	ESP(1) GUF(1)	H3
1764	■□□□□□□□□□□□□■■■■■■■■■■■■■■■□□□□□■□■■■■■■■■	2	KEN(2)	U
1765	■■■□□□□■■□■■□■■■■■■□□□□□□□□□□□□□□□□■■■■■■■■	5	KEN(1) UGA(1) USA(2) ZMB(1)	CAS1_KILI
1766	■■■■■□□□□■■■■■■■■■■■□□□□■■■■■■■■□□□□■■■■■■■	2	KEN(1) USA (1)	LAM9
1767	■■■■■■■■■■■■■■■□□□□□□□□□■■■■■■■■□□□□■■■■■■■	2	KEN(1) RUS(1)	T1
1768	■■■■■■■■■■■■■■■■■■□■□□□□■■■■□■■■□□□□■■■■■■■	2	CUB(1) MYS(1)	LAM6
1769	■■□■■■■■■■■■■■□□□■■■□□□□■■■■■■■■□□□□■■■■■■■	2	CUB(1) VEN(1)	LAM1
1770	□□□□□□□□□□□□□□□□□□□□□□□□■□□□□□□■□□□□■□■■■■■	2	FXX(2)	H2
1771	■■■■□■■■■■■■■■■■■■■■■■□□■□□□□□□■□□□□■■■■■■■	3	HUN(3)	H1
1772	■■■■■■■■□□■■■■■■■■■■■■■■■□□□□□□■□□□□□□□■■■■	2	FXX(1) HUN(1)	U
1773	■■■■■■■■■■■■■■■■■■■□■■□□■■■■■■■■□□□□■□□■■■■	10	HUN(10)	T1
1774	■■■■■■■■■■■■■■■■■■□■■■■■■■□■■■■■□□□□■■■■■■■	2	BEL(2)	T4
1775	□■□■■■■■■■■■■■■■■■■□□■■■■■■■□□□□■□■■■■■■■■■	2	BEL(1) GBR(1)	EAI2_MANILLA
1776	■■■■■■■■□□■■■■■■■■■■■■■□□□□□□□□□□□□□□□■■■■■	2	BEL(2)	U
1777	■■■□□□□■■■■■■■■■■□□□□□□□□□□□□□□□□□□□□□□□□■■	2	BEL(2)	CAS1_DELHI
1778	■■■■■■■■■■■■■■■□□□□□□□□□□□□□□□□□□□□□□■■■■■■	2	BEL(1) KEN(1)	U
1779	■■■■■■■■□■□□■■■■■■■■■■■■■■■■■■■■□□□□■■■■■■■	11	BEL(11)	T1
1780	□□□□□□□□□□□□□□□□□□□□□□□□□□□□□□□□□■□□■■■■■■■	14	BEL(14)	U
1781	■■□■■■■■■■■■■■■■■■■□□■■■■■■■□□□□■□■■■■■■■■□	2	BEL(1) USA(1)	EAI2_MANILLA
1782	■■■■■■□□□■■■■■■■■■■■□□□□■■■■■■■■■■■■■■□■■■■	2	BEL(1) GBR(1)	AFRI_1
1783	■■■■■■■■□□■■■■■■■■■■■■□□□■■■■■■■□□□□■■■■■■■	3	BEL(2) NLD(1)	LAM10_CAM-S
1784	■■□■■■■■□■■■■■□□□□■■■■■■■■■■■■■■■■■■■■□□□□□	2	BEL(1) DNK(1)	BOV
1785	■■■■■■□□□□□□□□□□□□□□□□□□■□□■■■■■□□□□■■■■■■■	7	BEL(7)	T1
1786	□□□□■■■■■■■□■□□■■■■■■■■■■■■■■■■■□□□□■■■■■■■	3	BEL(3)	T1
1787	■■■□□□□■■■■■■■■■■■■■■■□□□□□□□□□□□□■□□□■■■■■	2	BEL(1) SDN(1)	CAS1_DELHI
1788	■■■■■■■■■■■■□□□□■■■□□□□□□□□□□□□□□□□□□■■■■□■	3	BEL(3)	U
1789	■■■□□□□■■■■■■■■■■■■■■■□□□□□□□□□□□□□□□□■■■■■	4	BEL(1) PAK(1) SAU(1) USA(1)	CAS1_DELHI
1790	■■■■■■■■■■■■■■■■■■□□□□□□□□□□■■■■□□□□■■■■■■■	3	AUT(1) BEL(1) EGY(1)	T1

1791	■■□■■■■■■■■■■■■■■■■■□□□□■■■□□■■■□□□□■■■■■■■	2	BEL(2)	LAM1
1792	■□■■■■■■■■■■■■■■■■■■□□□□■■■■■■□■□□□□■■■■■■■	2	BEL(2)	H3-LAM9
1793	■■■■■■■■■■■■■■■■■■■■■■■■■■■■■■■■□□□□□□□□□□□	2	AUT(1) BEL(1)	U
1794	□■□■■■■■■■■■■■■■■■■■■□■■■■■■■■■■□□□□■■■□■■■	2	BEL(2)	T2
1795	■■■■■■■■■■■■■■■■■■■■■■□□□□□□□□□□□□□□□■■■■■■	2	AUT(1) BEL(1)	U
1796	□□□□□□□□□□□□■■■■■■■■■■■■■■■■■■■■□□□□■■■■■■■	2	BEL(2)	T1
1797	■■■■■■■■■■■■■■■■■■■■■■■■■■■■■□■■□□□□■■■□■■■	3	BEL(1) FXX(1) ITA(1)	T2
1798	■□■■■■■■■■■■■■■■■■■■■■□□■□□□□□□■□□□□■■■■■■■	2	BEL(2)	H1
1799	■■■■■■■■■■■■■■■■■■□■■■□□■■■■■■■■□□□□■□□□■■■	3	AUT(1) BEL(1) HUN(1)	T4-CEU1
1800	■■■■■■■■■■■■■■■■■■■□□□□□■■■■■■■■□□□□■■■■■■■	3	CHN(1) MWI(1) USA (1)	T1
1801	■■■■■■■■■■■■■■■■■■■■■■■■■■■■□□□□■□■■■■■□■■□	2	FXX(1) USA (1)	EAI1_SOM
1802	■■■■■■■■■■■■■■□■■■■■■■■■■■■■■■□■□□□□■■□□■■■	3	FXX(2) GLP(1)	H3
1803	■■□□□■■■■■■■■■■■■■■■□□□□■■■■■■■■□□□□■■■■■■■	3	ARG(1) FXX(1) SEN(1)	LAM9
1804	■■■■■■■■■■■■■■■■■■■□■■■■■■■■■■□■□□□□■■■■■■■	3	AUT(1) ITA(1) USA(1)	H3
1805	■■■■■■■■■■■■■■■■■■■■■■■■■■■■■■■□□□□□■■□■■■■	3	AUT(2) BRA(1)	U
1806	■■□■■■■■■■■■■□□■■■■■■■□■■■■■■■■■□□□□■■■■■■■	2	AUT(2)	T5
1807	■■■■■■■■■■■■■■■■■■■■■■■■■□□□□□□■□□□□■□■■■■■	2	AUT(2)	H1
1808	■■□■■■■■■■■■■■■■■■■■■□■■■■■■■■■□□□□■■■■■■■	2	AUT(1) BEL(1)	T5
1809	■■■□□□■■■■■■■■■■■■□■■■□□■■■■■■■□□□□□■□□■■■■	2	AUT(2)	U
1810	□■□□□□□□□□□□□□□□□■□■■■■■■■■□■■■■■■■□■■□□□□□	3	AUT(3)	CAP
1811	■■□■■■■■■□■■□□□□□□□□□□□□■□□■■■■■□□□□■■■□■■■	2	AUT(2)	T2
1812	■■■■■■■■■■■■■■■■■■■■□□■■■■■■■■□■□□□□■■■■■■■	3	AUT(2) VEN(1)	H3
1813	■■■■□■■■■■■■■■□■■■■■■■■■■□□□□□□■□□□□■■■■■■■	2	AUT(1) FXX(1)	H1
1814	■■■■■■■■■■■■■■■■■■■■□□□■■■■■□■■■□□□□■□■■■■■	2	AUT(2)	T1
1815	■■□■■■■■■■■■■■■■■■■■□□□□■■□■■■■■□□□□■■■■■■■	2	BRA(2)	LAM1-LAM8
1816	■■□■■■■■□■■■■■■□■■■■□■■■■■■■■□□□□□■■■■□□□□□	3	BRA(2) MEX(1)	BOVIS1
1817	■□□■■■■□□■□■■■□□■■■■■■■■■■■■■■■■■■■■■■□□□□□	2	BRA(2)	BOV
1818	■■□■□□□□□□□□□□■□■■■■■■■■■■■■■■■■□□■■■■□□□□□	2	BRA(1) MEX(1)	BOVIS3
1819	■□□■■■■■□■■■■■□□■■■■□■■■■■■■■■■■■■■■□■□□□□□	4	MEX(4)	BOV
1820	■■□■■■■■□■■■■■■□■■□■□■■■■■■■■■■■■■■■□■□□□□□	4	BRA(1) MEX(3)	BOVIS1
1821	■■■■■■■■■□■■■□□■■■■■■■■■■■■■■■■■□□□□■■■■■■■	2	ARG(1) NOR(1)	T1
1822	■■■■■■■■■■■■■■■■■■■■■■■■■□□□□□□■□□□□■■■■■■□	13	ARG(8) USA(5)	H1
1823	■■■■■■■■■■■■■■■■■□■■■■■■■■■■■■■■□□□□■■■■■■□	2	ARG(1) DEU(1)	X1
1824	■■■■■□□□□□■■■■■■■■■■■■■■■■■■■■■■□□□□■■■□□□■	4	ARG(2) BRA(1) PYF(1)	T1
1825	□□□□□□□□□□□□□□□□□□□□□□□□■□□□■□□■□□□□■■■■■■■	6	ARG(6)	H3
1826	■■■■■■■■■■■■□□■■■□■■■■■■■■■■■■■■□□□□■■■■■■■	2	ARG(1) GBR(1)	X1
1827	■■■■■■■■■■■■□□□□■■■■□□□□□□□□□□□■□□□□■■■■■■■	3	ARG(3)	H3
1828	■■■■■■■■■■■■■■■■■□□■□□□□■■■■■■■■□□□□■■■■■■■	3	ARG(1) NZL(2)	LAM9
1829	■■■■■■■□■■■■■■□■■■■■■■■■■■■■■■■■□□□□■■□■■■■	2	ARG(1) FXX(1)	T1
1830	■■■■■■■■□□□■■■■■■■□■□□□□■■■■■■■■□□□□■■■■■■■	3	ARG(1) CUB(1) ITA(1)	LAM3
1831	■■■■■■■■■■■■■■■■■■■■□■■□□■■■■■■□□□□□■■■■■■■	2	ARG(2)	U
1832	■■■■■■■■■■■■■■■□■■■■□□□□■■■■■■■■□□□□■■■■■■■	7	ARG(5) NZL(2)	LAM9
1833	■■■■■■■■■■■■□□□■□■□□□■■■■■■■□■■■□□□□■□■■■■■	2	ARG(2)	T1
1834	□■■■■■■■■■■■□■■■■■■■■■■■■■■■■■■■□□□□■■■■■■■	2	ARG(1) USA(1)	T3
1835	■■■■■■■■■■■■□□□□■■□■■■■■■■■■■■■■□□□□■■■□■■■	4	ARG(4)	T2

1836	■■■■■■■■■■■■■■■■■■□□□□□□■■■■■□■■□□□□■■■■□■■	3	ARG(2) FXX(1)	T1
1837	□□□□□□□□□□□□□□□□□□□□□□□□□□□□■□□□□□■■■■■■■■■	3	ARG(2) BRA(1)	U
1838	■■■■■■■■■□■■■■□■■■■■□□□□■■■■■■■■□□□□■■■■■■■	3	ARG(2) BRA(1)	LAM9
1839	■■■■■■■■■□■■□■□■■■■■□□□□■■■■■■■■□□□□■■■■■■□	2	ARG(2)	LAM5
1840	■■■■■■■■□□■■□■□■■■■■■■■■■■■■■■■■□□□□■■■■■■■	3	ARG(2) BEL(1)	S
1841	■■■■■■■■□□□■■■■■■■■■□□□□■■■■■■■□□□□□■■■■■■■	2	ARG(1) ZAF(1)	U
1842	■□□■■■■■□■■■■■□□■■■■■■■■■■■■■■■■■■■■■■□□□□□	2	ARG(1) BRA(1)	BOV
1843	■■■■□□□□□■■■■■■■■■■■■■■■■■■■■■■■□□□□■■■□■■■	2	ARG(2)	T2
1844	■■■■■■■■■■■■■■□□■■■■□□□□■■■■■■■■□□□□■■■■■■■	3	ARG(3)	LAM9
1845	□□□□□□□□□□□■■■■□■■■■■■■■■■■■■■■■■■■■■■□□□□□	4	ARG(3) BRA(1)	BOV
1846	■■□■□□□□□□□□□□■□■■■■■■■■■■□■■■■■■■■■■■□□□□□	2	ARG(2)	BOVIS3
1847	■■□■■■■■□■■■■■■□■■■■□■■■■■■□■■■■■■■■■■□□□□□	2	ARG(2)	BOVIS1
1848	■■□■■■■■□■■■■□□□□□■■□■■■■■■■■■■■■■■■■■□□□□□	3	ARG(2) MEX(1)	BOV
1849	■■□■■■■□□■□■■■■□■■■■■■■■■■■■■■■■■■■■■■□□□□□	6	ARG(5) BRA(1)	BOV
1850	■■□■■□■□□□□□■■■□■■■■■■■■■■□□□■■■■■■■■■□□□□□	4	ARG(4)	BOVIS2
1851	■□□■■□■□□□□□■■■□■■■■■■■■■■■■■■■■■■■■■■□□□□□	2	ARG(1) GBR(1)	BOV
1852	■■□■■■■■□■■■■■■□■■■■■■■■□□□□□□□□□□■■■■□□□□□	2	ARG(2)	BOVIS1
1853	■■□■■■■□□■■■■■■□■■■■□■■■■□□□■■■■■■■■□■□□□□□	2	ARG(2)	BOV
1854	■■□■■■■■□■■■■■■□■■■■■■■■□□□□□□□□□□□□■■□□□□□	2	ARG(1) FXX(1)	BOVIS1
1855	■■□■■□■□□□□□■■■□■■■■■■■■■■■■■■■■■■□■■■□□□□□	4	ARG(3) BRA(1)	BOVIS2
1856	■■□■■□■□□□□□■■■□■□■■■■■■■■■■■■■■■■■■■■□□□□□	6	ARG(6)	BOVIS2
1857	■■□■■■■■□■□■■■■□■■■■■■■■■■■□□■■■■■■■■■□□□□□	2	ARG(2)	BOVIS1
1858	□■□■■□■□□□□□■■■□□■■■■■■■■■■■■■■■■■■■■■□□□□□	4	ARG(4)	BOVIS2
1859	■■□□■□■□□□□□■■■□■■■■■■■■■■■■■■□□□□□□■■□□□□□	3	ARG(2) BRA(1)	BOV
1860	■■□■■□■□□□□□■■■□■■■■■■■■■■■■■■■□□□□■■■□□□□□	3	GBR(1) IRL(2)	BOVIS2
1861	■■□■■□■□□□□□■■■□■■■■■■■■■■■■■■□□□■■■■■□□□□□	5	GBR(1) IRL(4)	BOVIS2
1862	■■□■□□□□□□□□□□□□□□□□□□■■■■■■■■■■■■■■■■□□□□□	4	IRL(4)	BOV
1863	■■□■■□■□□□□□■■■□■■■■■■■■■■■■□■■■■■■■■■□□□□□	4	IRL(3) MEX(1)	BOVIS2
1864	■□□□□□□□□□□□□■□■■■■■■■■■■■■■□□□□■□■■■■□■■■■	2	USA(2)	EAI5
1865	■■■■□■■■■■■■■■□■■■■■■■■■■■■■□□□□■□■■■■□□■■■	3	USA(3)	EAI5
1866	■□□■■■■■■■■■■■■■■□□■■■■■■■■■□□□□■□■■■■■■■■■	3	NLD(1) USA(2)	EAI5
1867	■■■■■■□□□■■■■■■■■■■■■□□□■■■■■■■■■■■■■■□■□■■	2	NLD(1) USA(1)	AFRI_1
1868	■■□■■■■■■■■■■■■■■■■□□■■■■■■■□□□□■□■■■■■■■□■	2	PHL(1) USA(1)	EAI2_MANILLA
1869	■■■■■■■■■■■■□□□□□□□□□□□□□□□□□□□□□□□□□□□□□□□	3	NOR(1) USA(2)	U
1870	□□□■■■■■■■■■■■■■■■■■□□□□■■■■■■■■□□□□■■■□■■□	2	SWE(1) USA(1)	LAM4
1871	■■■□□□□□□□□□□■■■■■■■■■□■□□□□□□■■□□□□■■■■■■■	7	USA(7)	T5
1872	■■■■■■■■■■■■■■■■■■■■■■■■■□□□□□■■□□□□■■■■■■■	2	USA(1) ZWE(1)	T1
1873	■■■■■■■■■■■■■■■■□□■■□□□□■■□□□□■■□□□□■■■□■■■	2	USA(1) ZWE(1)	LAM11_ZWE
1874	■■■■■■■■□□■■■■■□□□□□□□□□□□■■■■■■□□□□□■■□■■■	2	USA(1) ZAF(1)	U
1875	■□□■■■■■■□□□□□□□□■■■■■■■■■■■□□□□■□■■■■■■■■■	2	DEU(1) USA(1)	EAI5
1876	■■■■□□□□□□□□■■■■■■■■■■■■■■■■■■■■■■■■□■■■■	2	USA(2)	AFRI
1877	■■■□■■■■■□■■■■■■■■■■■■■■■■■■■■■■□□□□■■■■■■■	2	USA(2)	T1
1878	■■■□□□□■■■■■■■■■■■■■■■□□□□□□□□□□□□■■■■□□□□□	2	USA(2)	CAS1_DELHI
1879	■■■■■■■■■■■■□■■■■■■□□□□□□□□■□□□□■□■■■■■□■■■	2	IND(2)	EAI1_SOM
1880	□□□□■■■■■■■□□□□□■■■■□□□□■■■■□□□□□□□□□□□□□■■	3	USA (3	U

1881	□□□■■■■■■■■■■■■■■■□■■■■■■■■■□□□□■□■■■■■■■■■	3	FXX(1) SAU(2)	EAI5
1882	■■■□□□□■■■■■■■■■■□■■□□□□□□□□□□□□□□■■■■■■■■■	2	SAU(1) USA(1)	CAS1_DELHI
1883	■■■□□□□■■■■■■■■■■■■■■■□□□□□□□□□□□□■■■■■■■□□	2	SAU(2)	CAS1_DELHI
1884	■■■■■■■■■■■■■■■■□□■■■■■■■■■□□□□□□□□□□□■■■■■	2	AUT(1) SAU(1)	U
1885	□□□□■■■■■■■□□■■■■■□■■■■■■■■■■■■■□□□□■■■■■■■	3	SAU(3)	T1
1886	■■□□■■■■■■■■■■■■■■■□□■■■■■■■□□□□■□■■■■■■■■■	3	PHL(1) SAU(2)	EAI5
1887	■■■□□□□■■■■■■■■■■■■■■□□□■■■■■■■■■□□□□■■■■■■	4	SAU(4)	U
1888	■■□■■■■■■■■■■■■■■■■■■□□□■■■■■■■■□□□□■■■■■■■	3	BRA(2) VEN(1)	T1
1889	□□□□□□■■■■■■■■■■■■■■■□□□■■■■■■■■□□□□■■■■■■■	2	BRA(2)	T1
1890	■■■■■■■■■■■■■■■■■■■■■□□□■■■■■■■■□□□□■■■□■■■	2	BRA(2)	T2
1891	■■■■■■■■■■■■■■■■■■■■■■■■□■■■■■□■□□□□■■■■■■■	3	BRA(3)	H3
1892	■■■■■■■■■■■■■■■■■■■■□■■□□□□□□□□□□□□□□□■■■■■	2	BRA(2)	U
1893	■■■■■■■■■■■■■■■■■■■□■■■■■■■■■□□■□□□□■■■■■■■	2	BRA(2)	H3
1894	■■■■□□■■■■■■■■■■■■■■□□□□■■■■■■■■□□□□■■■■■■■	2	BRA(2)	LAM9
1895	■■■■■■■■■■■■■■■■■■■■□□□□■■■■□□■■□□□□■■■□■■■	2	BRA(2)	LAM4
1896	■■■■■■■■■■■□□■■■■■■■■■■■■■■■■■■■■□□□■■■□□■■	2	BGD(2)	MANU3
1897	■■■■■■■■■■■■■■■■■■■■■■■■□□□□■□□□□□■■□■■■■■■	10	BGD(10)	U
1898	■■■■■■■■■■■■■■■■■■■■■■■■□□□□□□□□□□■■□■■■■■■	24	BGD(24)	EAI7_BGD2
1899	■■□■■■■■■■■□■□□□□□□□□□□□■■■■■■■■□□□□■■■■■■■	2	VNM(2)	T1
1900	■□■■■■■■■■■■■■■■■■■■■■■■■■■■□□□□■□■■■■■■■■□	2	VNM(2)	EAI5
1901	■■■■■■■■■■■■■■■■■■■■■□□■■□□■□□□□■□■■■■■■■■■	2	AUS(1) VNM(1)	EAI4_VNM
1902	■■■□□□■■■■■■■■■■■■■■■■■■■□□■□□□□■□■■■■■□■■■	4	VNM(4)	EAI1_SOM-EAI4
1903	■■■□■■■■■■■■■■■■■■■■■■■■■□□■□□□□■□■■■■■■■■■	2	VNM(2)	EAI4_VNM
1904	□□□□■■■■■■■□■■■■■□□□□□□□□□□□□□□□□□□□□■■■■■■	3	AUT(1) ITA(1) VNM(1)	U
1905	■■■■■■■■■■■■■■■■■■■■■■■■■■■■□□■■□□□□■■■■■■■	2	ARG(1) BRA(1)	T1
1906	■■■■■■■■■■■■■■■■■■■■□□□□■□■■□■■■□□□□■■■■■■■	3	BRA(3)	LAM6
1907	■■■■■■■■■■■■■■■■■■■■■■■■■■■■■■■■■■□□■■■■■■■	2	BRA(2)	U
1908	■■■■■■■■■■■■■■■■■■■■■■■■■■□□□□□■□□□□■■■■■■■	2	BRA(1) USA(1)	H3
1909	■■■■■■■□■■■■■■■■■■■■■■■■■■■■■■■■□□□□■■■□□□□	3	BRA(2) VEN(1)	T1
1910	■■□■■■■■■■■■□■■■■■■■■□□□■■■■■■■■□□□□■■■■■■■	2	BRA(2)	T3
1911	■■□■■■■■■■■■□■■■■■■■■□□□■■■■■■■■□□□□■■■■■■□	2	BRA(2)	T3
1912	■■■■■■■□■■■■■■□■■■■■■■□■■■■■■■■■□□□□■■■■■■■	2	ARG(1) MTQ(1)	T5
1913	■■■■■■■■■■■■■■□■■■■■■■□■■■■■■■■■□□□□■■■■■■■	2	FXX(1) GLP(1)	T5
1914	□■■■■■■■■■■■■■■■■■■■□□□□■■■■□■■■□□□□■■■■■■■	2	ITA(1) VEN(1)	LAM6
1915	■■■■■■■■□□■■■■■■■■■■■□■■■■■■■■■■□□□□■■■■■■■	2	ITA(1) MTQ(1)	S
1916	■■■■■■■■■■■■□□□□□■■■■■■■■■■■■■■■□□□□■■■■■■■	5	ITA(1) SEN(1) TUR(3)	T1
1917	■□■■■■■■■■■■■■■■■■■■■■■■■■■■■■■■□□□□■■■■■■□	2	ITA(2)	T1
1918	■■■■■■■■□□■■■■■■■■■■■■■■■■■■■■□□□□□□■■■■■■■	3	ITA(3)	U
1919	■■■■■■■■■■■■■■■■■■□■■■■■■■■■■■□■□□□□■■■□■■■	2	ITA(2)	H3
1920	■■■□□□□■■■■■■■■■■■■■■■■■□□□□□□□□□□□■■■■■■■■	15	ITA(15)	U
1921	■□■■■■■■■■■■■■■■■■■□□□□□■■■■□■■■□□□□■■■□■■■	2	ITA(2)	T2
1922	■■■■■■■■■■■■□□□□■□■■■■■■■■■■■■■■□□□□■■■■■■■	2	ITA(1) USA (1)	X1
1923	■■■■□■■■■■■■■■■■■■■■■■■■■■■■■■□□□□□□■■■■■■■	2	ITA(1) USA (1)	U
1924	■□■■■■■■■■■■■□□■■■■□□□□□■□□■■■■■□□□□■■□■■■■	3	EGY(1) ITA(2)	LAM7_TUR
1925	■■■■□■■□□□□■□□□□□□■■■■■■■■■■■□■■■■■■■■■■■□■	2	ITA(2)	U

1926	■■■■■■■■■■■■■■■■■■■■■■■■■■■■■■■■□□□□■■■□□□■	2	ARG(1) ITA(1)	T1
1927	■■■■■■■■■■■■□■■■■■■■■■■□■□□□□□□■□□□□■■■■■■■	2	AUT(1) ITA(1)	H1
1928	■■■■□■■■■■■■■■■■■■■■■■■■■■□□□□□□□□□□■■■■■■■	3	ITA(2) POL(1)	U
1929	■■■■■■■■■■■■■■■■■■■■■■■■■■■■■■■■□□□□□■■□□□□	2	ITA(1) USA (1)	U
1930	■□□■■■■■■■■■■■■■■■■■■□■■■■■■■■■■□□□□■■■■■■■	2	ITA(2)	T1
1931	■■■■■□■■■□□□□□□□□□□□□□□□■■■■■■□■□□□□■■■□■■■	2	ITA(2)	H3
1932	■■□■■■■■□■■■□■■□■■■■■■■■■■■■■■■■■■■■■□□□□□	2	FXX(1) ITA(1)	BOVIS1
1933	■■■■■■■■■■■■■■■■■■■■□□□□■■■■■■■■□□□□■■■■■■□	2	ITA(1) USA (1)	LAM9
1934	■■■■■■■■■■■■■■■■■■■■■□■□■□□□□□□■□□□□■■■■■■■	2	ITA(1) NLD(1)	H1
1935	■■□■■■■■■■■■■■□■■■■■□□□□■■■■■■■■□□□□■■□■■■■	2	GUF(1) PRT(1)	LAM1
1936	■□■■■■■■■■■□■■■■■■□□□■■■■■■■■■■■□□□□■■■■■■■	2	SWE(1) TUR(1)	T1
1937	■■■■■■■■■■■■■■■■■■■□□□□□■□□■■■■■□□□□■■■■■□□	2	TUR(2)	LAM7_TUR
1938	■■■■■■■■□□■■■■■■■■■■■■■■■■■■■■■■□□□□■■■■■■□	3	IDN(1) TUR(2)	S
1939	■■■■■■■■■■■■■■■■■■■■■■■■■□■■■■□■□□□□■■■□■■■	2	USA(2)	H3
		35925		
